# Chemical diversity, medicinal potentialities, biosynthesis, and pharmacokinetics of anthraquinones and their congeners derived from marine fungi: a comprehensive update†

**DOI:** 10.1039/d2ra03610j

**Published:** 2022-09-01

**Authors:** Mohamed Sebak, Fatma Molham, Claudio Greco, Mohamed A. Tammam, Mansour Sobeh, Amr El-Demerdash

**Affiliations:** Microbiology and Immunology Department, Faculty of Pharmacy, Beni-Suef University Beni-Suef 62514 Egypt; Molecular Microbiology Department, The John Innes Center Norwich Research Park Norwich NR4 7UH UK; Department of Biochemistry, Faculty of Agriculture, Fayoum University Fayoum 63514 Egypt; AgroBioSciences Department, Mohammed VI Polytechnic University (UM6P) Ben Guerir Morocco; Organic Chemistry Division, Department of Chemistry, Faculty of Science, Mansoura University Mansoura 35516 Egypt a_eldemerdash83@mans.edu.eg Amr.El-Demerdash@jic.ac.uk +00447834240424; Department of Metabolic Biology and Biological Chemistry, The John Innes Center Norwich Research Park Norwich NR4 7UH UK

## Abstract

Marine fungi receive excessive attention as prolific producers of structurally unique secondary metabolites, offering promising potential as substitutes or conjugates for current therapeutics, whereas existing research has only scratched the surface in terms of secondary metabolite diversity and potential industrial applications as only a small share of bioactive natural products have been identified from marine-derived fungi thus far. Anthraquinones derived from filamentous fungi are a distinct large group of polyketides containing compounds which feature a common 9,10-dioxoanthracene core, while their derivatives are generated through enzymatic reactions such as methylation, oxidation, or dimerization to produce a large variety of anthraquinone derivatives. A considerable number of reported anthraquinones and their derivatives have shown significant biological activities as well as highly economical, commercial, and biomedical potentialities such as anticancer, antimicrobial, antioxidant, and anti-inflammatory activities. Accordingly, and in this context, this review comprehensively covers the state-of-art over 20 years of about 208 structurally diverse anthraquinones and their derivatives isolated from different species of marine-derived fungal genera along with their reported bioactivity wherever applicable. Also, in this manuscript, we will present in brief recent insights centred on their experimentally proved biosynthetic routes. Moreover, all reported compounds were extensively investigated for their *in-silico* drug-likeness and pharmacokinetics properties which intriguingly highlighted a list of 20 anthraquinone-containing compounds that could be considered as potential drug lead scaffolds.

## Abstract 2

Marine-derived fungi receive excessive attention as prolific producers of structurally unique secondary metabolites. Whilst they are promising substitutes or conjugates for current therapeutics, so far research has only touched on their secondary metabolite diversity.

## Graphical abstract

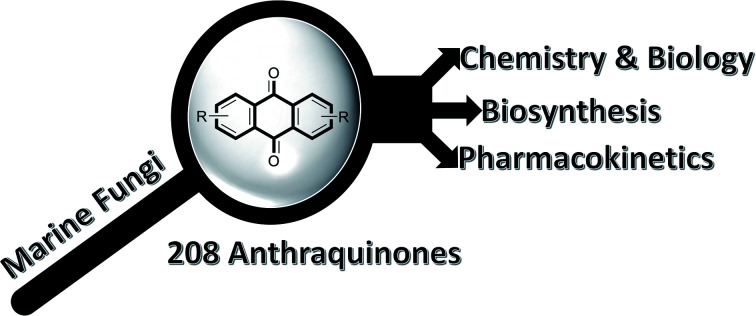

## 1 Introduction

Throughout history, different natural sources have been used for treatment of diseases, and more recently as sources and valuable suppliers of biologically active compounds with diverse bioactivities that can be developed to be used in new drugs.^1–6^ Intriguingly, marine organisms and microorganisms were among the valuable sources of new natural products.^2^ Microbial secondary metabolites have been known for their chemical diversity and a broad range of bioactivities.^6,7^ Marine microorganisms are considered highly productive sources of physiologically active compounds including peptides, polyketides, terpenes, and alkaloids.^8–10^ Some marine-based compounds have been approved as drugs with different pharmacological uses,^11,12^ while several others are under different clinical trials before their approval as new drugs.^11^

During the last few decades, numerous drug discovery programs focused on marine-derived microbial natural products due to their great potential for the production of structurally diverse biologically active secondary metabolites.^13,14^ Among the hot microbes responsible for the production of interesting compounds, fungi, served as the primary source for mining the first reported antibiotic, penicillin, whereas they are still one of the main sources for discovering novel bioactive compounds from different niches including the marine fungi which have high biological diversifications.^15,16^ Therefore, the bioactive secondary metabolites recovered from the marine-derived fungi have gained great interest as promising sources of therapeutics. Interestingly, more than a thousand compounds have been isolated from marine fungi with a wide range of bioactivities including antiviral, anticancer, and antibacterial activities.^17^ Even though only one bioactive compound, cyclosporine A, has been approved for clinical use in the market. This might be attributed to problems in the optimization methods or the screening approaches of natural product discovery.^18^

Studying the marine-derived fungi has been started around two centuries ago when the first fungal species, *Sphaeria posidoniae* (*Halotthia posidoniae*) was reported on a rhizome of the marine grass *Posidonia oceanica* in 1846.^19^ Marine fungi have been isolated from different habitats including algae, mobile, and sessile invertebrates, sediments, marine mammals, and driftwood from different marine locations.^20^ Despite the importance of marine fungi as a promising source for novel bioactive secondary metabolites, marine fungi are still less investigated sources for natural product discovery programmes compared to other niches of fungi.^18,21^ Although the estimated number of fungal species on the earth is ranging from 1.5 to 5 million species, only around 1100 species have been exclusively isolated from the marine niche.^18,20^

Marine-derived fungi produce various classes of different compounds with both chemical and biological diversities.^22,23^ For instance, they produce varieties of bioactive compounds such as terpenes, alkaloids, peptides, and polyketides.^18^ Polyketides have been reported in many previous studies as dominant natural products from marine filamentous fungi.^24,25^ They are a large group of complex chemical architectures such as anthraquinones, hydroxyanthraquinones, naphthoquinones, macrolides, flavonoids, polyenes, and tetracyclines. Around 700 anthraquinones and their derivatives have been reported from different natural sources, while anthraquinones are widely produced by marine filamentous fungi.^16,26^ Chemically, anthraquinones are a group of polyketides of the quinone family with a basic cyclic scaffold of three fused benzene rings including two ketone groups on the central 9, 10-carbons with a chemical formula of C_14_H_8_O_2_, while their derivatives are generated by the decoration of the around free protons with different functional groups^16^ or by enzymatic reaction of the rings or the keto groups such as reduction, oxidation, dehydration or dimerization to result in a wide range of derivatives.^27^ Interestingly, many reported anthraquinones and their derivatives exhibited potent biological activities including antitumor, antibacterial, antifungal, antioxidant, and immunomodulatory bioactivities.^16^

Drug-likeness and pharmacokinetics properties using SWISSADME online platform, which intriguingly highlighted a list of 20 anthraquinone containing compounds (ESI†) that could be considered as potential drug leads scaffolds. Such a massive connection between chemical spaces and bioactivities highlights the huge capacity of marine-derived fungi as an attractive biological source that is worth further exploitations with distinguished anticipations for the global pharmaceuticals industries.

Several interesting review articles have focused recently on the marine anthraquinones and their derivatives such as Fouillaud *et al.* who reported the chemical diversity, specific bioactivities, biosynthetic pathways, biological sources, and the producing fungal genera of tens of marine-derived anthraquinones and their derivatives discovered before 2016.^16^ Also, another review by Masi and Evidente presented a comprehensive update of the bioactive fungal anthraquinones and analogues including the marine-derived anthraquinones produced *via* the acetate route over the period 1966–2020 with their sources, biosynthesis, biological activities, and industrial applications.^28^ Whereas Greco *et al.* in their recent review critically described the marine-derived anthraquinones which showed anti-tumor activity as well as their mutagenic and genotoxic potentialities.^29^ Herein and as a part of our continuous program on pharmacologically active fungal natural products,^4,30,31^ we are presenting an extensive coverage over the period 2000–2020 for 208 anthraquinones and their derivatives, extensively reported from different marine-derived fungal genera such as *Nigrospora*, *Aspergillus*, *Penicillium*, *Stemphylium*, *Alternaria*, *Eurotium*, *Trichoderma*, *Halorosellinia*, and *Fusarium*. In addition, we reported here their different biological activities, drug-likeliness and pharmacokinetics properties wherever applicable, in addition to a general overview of their proposed biogenesis pathway. The investigation of *in-silico* drug-likeness and pharmacokinetics properties of the marine-derived anthraquinones and their derivatives in this review could be advantageous in predicting the possibility of anthraquinones as drug candidates.

## 2 General biosynthetic pathway of anthraquinones

**Fig. 1:**
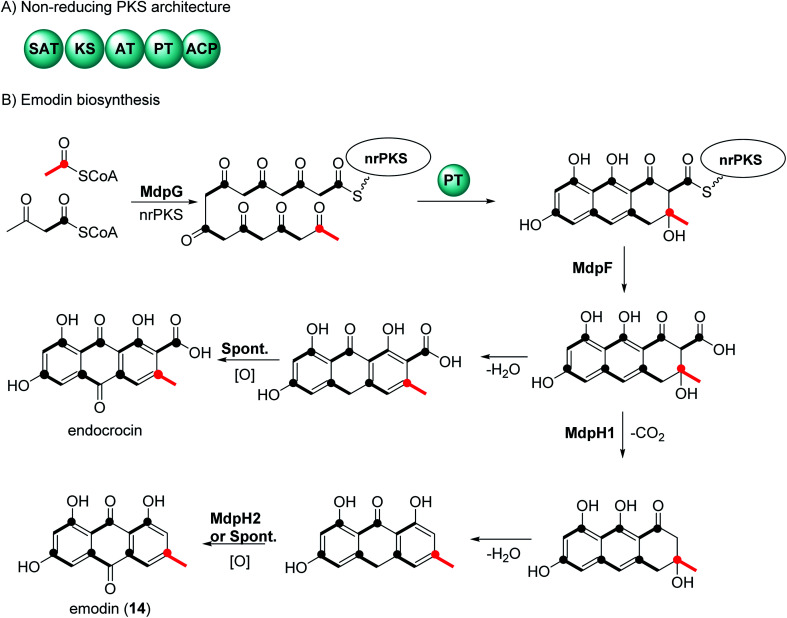
General biosynthetic pathway of anthraquinones in fungi. (A) domain architecture of the non-reducing polyketide synthase. (B) Biosynthetic pathways of the anthraquinones emodin (14) and endocrocin. The isotope labelling pattern is shown black bold lines and the polyketide starter unit is indicated in red.

There have been extensive studies since the 1950s to determine the biosynthetic pathway of anthraquinones and the related natural products called xanthones.^32,33^ Feeding experiments using labelled acetates in fungi, first reported by Birch *et al.*, showed that anthraquinone and xanthones are biosynthesized by polyketides.^32,33^ Genome sequencing and genetic transformation experiments have confirmed that the core structure of anthraquinones is synthesized in fungi by non-reducing polyketide synthase (nrPKS).^34,35^ This class of PKS share a common domain architecture which consists of an SAT (starter unit-ACP transacylase), KS (ketosynthase), AT (acyl transferase), PT (product template), and ACP (acyl carrier protein) (Fig. 1A). The biosynthesis of anthraquinones can be generalized using emodin (14) and endocrocin as examples (Fig. 1B).^27,36^ The nrPKS (MdpG) synthesize the polyketide, which is then cyclized with the loss of two water molecules by the PT domain. The polyketide is released by a metallo-hydrolase protein (MdpF) to obtain atrochrysone carboxylic acid, which can in most cases, undergo decarboxylation by a decarboxylase (MdpH1). This is followed by spontaneous dehydration and oxidation by an anthroneoxidase (MdpH2) to afford emodin (14).^27,36^ Some reports also have described that the final oxidation step could occur spontaneously.^36^ Further modification by tailoring proteins give rise to a huge diversity, these include methylation, dehydration, and dimerization.^27^

## 3 Chemistry and medicinal potentialities of anthraquinones and their congeners derived from marine-derived fungi

In this manuscript, we provide extensive insights about chemical and biological investigations centered on anthraquinones and their derivatives exclusively derived from marine fungi. For the handling of this documentation, all isolated anthraquinones are classified and tabulated according to the marine fungal genera where they have been recovered along with their recorded biological potentialities whenever applicable.

### 3.1. Anthraquinones isolated from *Nigrospora* sp.

Ten anthraquinones or their derivatives 1–10 were reported from the marine-derived fungus *Nigrospora* sp. Nigrodiquinone A (1) was isolated for the first time as a new hydroanthraquinone dimer from the zoanthid-derived fungus *Nigrospora* sp.^37^ Another four anthraquinone derivatives namely 4a-*epi*-9α-methoxydihydrodeoxybostrycin (2), 10-deoxybostrycin (3), 3,5,8-trihydroxy-7-methoxy-2-methyl-anthracene-9,10-dione (4), and austrocortirubin (5) were reported from both sea anemone-derived^38^ and zoanthid-derived fungus *Nigrospora* sp.,^37^ while austrocortirubin (5) was also recorded from the sea fan-derived fungus *Fusarium* sp.,^39^ and the mangrove endophytic fungi *Guignardia* sp.^40^ and *Halorosellinia* sp.^40,41^ Although nigrodiquinone A (1) showed no antiviral or antibacterial activities,^37^ compounds 4 and 5 displayed mild antiviral activity with IC_50_ value of 93.7 μM against coxsackievirus and 74.0 μM against the respiratory syncytial virus (RSV), respectively.

Notably, compounds 2 and 3 showed potent antibacterial activity against both the Gram-positive bacteria, *Staphylococcus aureus* and *Micrococcus tetragenus* and the Gram-negative bacteria, *Escherichia coli* (*E. coli*), *Vibrio anguillarum* (*V*. *anguillarum*), and *V. parahemolyticus*. Compound 3 displayed MIC of equal to or less than 2.5 μM against all tested bacteria, whereas compound 2 exhibited MIC of equal to or less than 2.5 μM against all tested bacteria except *V. anguillarum* and *V. parahemolyticus* against which it showed MIC value of 25.0 μM.^38^ In addition, compound 3 showed potent cytotoxic activity against the human lung cancer cell line, A-549 with an IC_50_ value of 4.56 μM,^38^ while austrocortirubin (5) displayed an IC_50_ value of 6.3 μM against the human breast adenocarcinoma cells, MCF-7 ^39^.

Further anthraquinone derivatives 6–10 were previously isolated from the sea anemone-derived fungus *Nigrospora* sp.^38^ Also, some of these anthraquinone derivatives have been isolated from other marine fungal species such as *Fusarium* sp. PSU-F14 from which compounds 6–8 and 10 were recovered,^39^ while compounds 7, 8 and 10 were also isolated from the marine-derived fungus *Aspergillus* sp.^42^

Compounds 6–10 exhibited different interesting biological activities. For instance, nigrosporin B (6) displayed modest anti-mycobacterial activity,^43^ phytotoxic activity,^44^ and potent antibacterial and cytotoxic activity.^38^ Also, 4-deoxybostrycin (9) showed modest anti-mycobacterial activity,^43^ potent antibacterial activity,^38^ and moderate antitumor activity.^45^ Nigrosporin B (6) and 4-deoxybostrycin (9) displayed potent antibacterial activity against both the Gram-positive bacteria, *Bacillus subtilis* (*B. subtilis*), *B. cereus*, *Staphylococcus albus* (*S. albus*), *S. aureus*, and *Micrococcus tetragenus* and the Gram-negative bacteria *E*. *coli*, *V. anguillarum*, and *V. parahemolyticus* with MIC values equal to or less than 2.5 and 3.12 μM, respectively.^38^ Moreover, both compounds exhibited modest anti-mycobacterial activity against several mycobacterial species including two multidrug-resistant *Mycobacterium tuberculosis* (*M*. *tuberculosis*) with MIC values of less than 30.0 μg mL^−1^.^43^

**Fig. 2:**
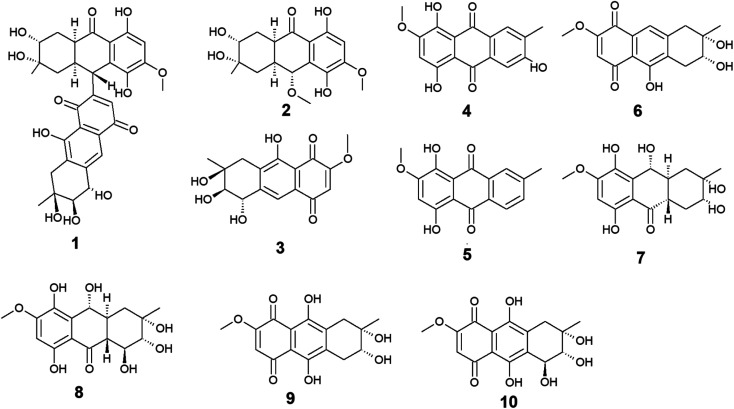
Chemical structures of compounds 1–10.

An additional example of anthraquinones isolated from *Nigrospora* sp. with multiple bioactivities is tetrahydrobostrycin (8) which exhibited moderate to high antibacterial activity against the Gram-positive bacteria, *B. subtilis* and *B. cereus* (MIC values of 2.5 μM), *S*. *aureus* and *Micrococcus luteus* (MIC values of 2.5 μM), and *Micrococcus tetragenus* (MIC value of 1.25 μM).^38^ Compound 8 also displayed good antibacterial activity against the Gram-negative bacteria *E. coli* (MIC value of 6.25 μM), *V. anguillarum* (MIC value of 1.56 μM), and *V. parahemolyticus* (MIC value of 12.5 μM).^38^ Additionally, it exhibited potent activity against *M. tuberculosis* with a MIC value of 12.50 μg mL^−1^ and was also active as antimalarial agent against *Plasmodium falciparum* with an IC_50_ value of 7.94 μg mL^−1^^46^ (Fig. 2).

### 3.2. Anthraquinones isolated from *Aspergillus* sp.

*Aspergillus* was the richest source of marine anthraquinones and their derivatives among all marine-derived fungi with 73 reported compounds including the previously mentioned 7, 8, and 10 as well as other seventy anthraquinones 11–80. For instance, thirteen compounds 11–23 were isolated from the marine-derived fungus *Aspergillus glaucus* (*A*. *glaucus*).^47^ Aspergiolide A (11), which features a naphtho[1,2,3-de]chromene-2,7-dione skeleton was isolated as a novel anthraquinone derivative from the marine-derived fungus *A. glaucus*.^48^ Aspergiolide B (12) was isolated from *A*. *glaucus* as a new analogue for aspergiolide A (11).^47^ Aspergiolides A and B (11 and 12) exhibited potent cytotoxic activities against both adenocarcinoma human alveolar basal epithelial cell line, A-549 with IC_50_ values of 0.13 and 0.24 μM and human leukemia cell line, HL-60 with IC_50_ values of 0.28 and 0.51 μM, respectively^47,48^ indicating that methylation of one hydroxyl group in aspergiolide A (11) to be a methoxy group in aspergiolide B (12) slightly affected the cytotoxicity of aspergiolide A.

Physcion (13) was also isolated from other species of *Aspergillus* such as *A. glaucus*,^47^*A. wentii* ,^49^ and the halotolerant *A. variecolor*^50^ besides the marine-derived fungus *Microsporum* sp.^51^ Physcion (13) displayed a wide array of biological activities including cytotoxic activity against human cervical carcinoma HeLa cells,^51^ moderate antifungal activity against *Trichophyton mentagrophytes* with a MIC value of 25.0 μg mL^−1^ and weak antifungal activity against both *C. albicans* and *Cryptococcus neoformans* with MIC value of 50.0 μg mL^−1^.^52^ It also exhibited weak free radical scavenging activity against 1,1-diphenyl-2-picrylhydrazyl (DPPH) with an IC_50_ value of 99.4 μg mL^−1^.^49^

Furthermore, emodin (14) which was reported from the marine-derived fungus *A. glaucus*, was also recovered from many other marine fungal species such as *Penicillium citrinum* (*P. citrinum*),^53^*Trichoderma aureoviride* (*T*. *aureoviride*),^54^*Monodictys* sp.,^55^*Gliocladium* sp.,^56^*Paecilomyces* sp.,^57^*Eurotium rubrum* (*Eu. rubrum*)^58^ and *A. versicolor*.^59^ Emodin (14) showed moderate antibacterial against *Pseudomonas putida* with a MIC value of 25.0 μM^60^ and significant anti-mycobacterial activity against *M. tuberculosis* with a MIC value of 12.5 μg mL^−1^ and modest antifungal activity against *Candida albicans* (*C*. *albicans*) with an IC_50_ value of 11.0 μg mL^−1^.^61^ Noteworthy, it showed potent cytotoxic activity against both oral human epidermoid carcinoma cell line, KB and human breast cancer cell line, MCF7 with IC_50_ values of 0.88 and 2.8 μg mL^−1^, respectively.^61^

Further anthraquinones 17 and 18, and 20 which were isolated from both *A. glaucus*^47^ and the halotolerant *A. variecolor*,^50^ showed variable bioactivities. Questin (17) and catenarin (18) exhibited DPPH radical scavenging activity^62^ and potent antibacterial activity against *Brevibacillus brevis* with a MIC value of 1.0 μg mL^−1^,^63^ respectively, while (+)-variecolorquinone A (20) displayed positive cytotoxicity against the human hepatocellular carcinoma cell line, BEL-7402, mouse lymphoma cell line, P388, human leukemia cell line, HL-60, and adenocarcinoma human alveolar basal epithelial cells, A-549 with IC_50_ values of 114.0, 266.0, 309.0, and 3.0 μM, respectively.^50^

Notably, the known anthraquinone dimer 21, as well as two new isomers of anthraquinone dimer 22 and 23, were also isolated from *A. glaucus*.^47^ However, compound 21 was not evaluated for any relevant bioactivity, the *trans* isomer of emodin-physcion bianthrone (22) showed good cytotoxicity against the cell lines; A-549 and HL-60 with IC_50_ values of 9.2 and 7.8 μM, respectively. On the other hand, its *cis* isomer 23 was less active as its IC_50_ values were 14.2 and 44.0 μM, respectively,^47^ suggesting that isomerization has affected the cytotoxicity of compound 22.

Additional thirty anthraquinones 24–54 have been isolated from the marine-derived fungus *A. versicolor.* Two new anthraquinone dimers 24 and 25 besides three other known closely related anthraquinone derivatives 26–28 were isolated from the marine-derived fungus *A. versicolor*.^64^ Averantin (26) and its derivative 1′-*O*-methyl-averantin (27) were also isolated earlier from the marine-derived fungus *P. purpurogenum* G59 ref. 65 and *Aspergillus* sp. SCSIO F063 ,^66^ while averythrin (28) was formerly reported from the marine-derived fungus *Aspergillus* sp. SCSIO F063.^66^

Compounds 24 and 25 showed selective antibacterial activity against the Gram-positive bacterium, *S*. *aureus* using the disk diffusion method at a concentration of 30.0 μg per well,^64^ whereas the same study revealed that compound 24 had a selective cytotoxic activity against human CNS cancer cells, XF-498 with an IC_50_ value of 22.39 μg mL^−1^. In addition, averantin (26) and its derivative 1′-*O*-methyl-averantin (27) displayed a weak antitumor activity against the bone marrow cancer cell line, K562 at a concentration of 100.0 μg mL^−1^.^65^ Another study mentioned that compound 27 exhibited modest cytotoxic activity against the human glioblastoma SF-268, human breast adenocarcinoma MCF-7 and human large-cell lung carcinoma NCI–H460 cell lines with IC_50_ values ranging from 33.59 to 44.22 μM, whilst compounds 26 and 28 displayed weak to moderate cytotoxic activity against MCF-7 with IC_50_ values of 45.47 and 29.69 μM, respectively.^66^ Also, compounds 26 and 27 displayed potent antioxidant activity, whereas compound 28 exhibited weak antioxidant activity in terms of antioxidant capacity compared to Trolox^67^ suggesting that the presence of oxygen in the side chain of the anthraquinones may play role in their antioxidant activity.

Additionally, compound 26 displayed promising antibacterial activity against different strains of the Gram-positive bacteria, *Streptococcus pyogenes* (*Str. pyogenes*) and *S*. *aureus* with MIC values of equal to or less than 3.13 μg mL^−1^, while its 1′-*O*-methylated derivative, 27 showed weaker antibacterial activity as it was only active against one strain of *Str*. *pyogenes* with a MIC value of 6.25 μg mL^−1^ with no activity against the other strain of *Str*. *pyogenes* or any strain of *S*. *aureus* up to a concentration of 12.5 μg mL^−1^,^68^ indicating that *O*-methylation at position 1 greatly affected the antibacterial activity of averantin (26).

Compound 29 which is another derivative of averantin (26) was isolated from another marine-derived fungus *A. versicolor* EN-7.^69^ Compound 29 showed weak antibacterial activity against only *E. coli* at a concentration of 20.0 μg per disk with no activity against *S*. *aureus*,^69^ suggesting that the *O*,*O*′-dimethylation of averantin (26) decreased its antibacterial activity against the Gram-positive bacteria.

The aflatoxin, averufin (30) and its O-methylated derivatives 6-*O*-methyl-averufin (31) and 6,8-*O*,*O*′-dimethyl-averufin (32) were also isolated from different strains of the marine-derived fungus *A*. *versicolor*,^68,69^ whereas averufin (30) was also isolated from other species of *Aspergillus* such as *A. niger*^70^ and *A. nidulans*.^71^ Averufin (30) exhibited different bioactivities including potent antioxidant activity in terms of Trolox equivalent antioxidant capacity,^67^ weak cytotoxic activity,^68^ and moderate inhibitory activity against the multiplication of *Tobacco Mosaic* virus,^70^ in addition to weak antibacterial activity against the Gram-positive *Str*. *pyogenes* and *S*. *aureus* with MIC values equal to or less than 12.5 μg mL^−1^.^68^ On the other hand, neither 6-*O*-methyl-averufin (31) nor 6,8-*O*,*O*′-dimethyl-averufin (32) showed any antimicrobial activity^69^ or anti-neuroinflammatory effect,^72^ respectively.

Moreover, further eight bioactive compounds 33–40 were also isolated from the marine-derived fungus *A. versicolor*^67–69,73^ including versicolorin B (33), averufanin (35) nidurufin (37), and versiconol (39) as well as their derivatives 1′-hydroxyversicolorin B (34), noraverufanin (36), 6,8-*O*,*O*′-dimethyl-nidurufin (38) and 6,8-*O*,*O*′-dimethyl-versiconol (40), respectively. Both versicolorin B (33) and its hydroxyl derivative, 1′-hydroxyversicolorin B (34) showed potent antioxidant activity as they displayed antioxidant capacity approximately equivalent to Trolox,^67^ while an old study revealed that 1′-hydroxyversicolorin B (34) (UCT1072M1) had potent cytotoxicity against the human cervical cell adenocarcinoma, HeLa S3 and the human lung giant cell carcinoma, Lu-65 with IC_50_ values of 2.1 and 2.2 μM, respectively.^74^

Indeed, averufanin (35) displayed a good antioxidant activity in terms of antioxidant capacity to Trolox,^67^ and weak activity against both acyl-CoA: cholesterol acyltransferase type 1 and 2 in the cell-based assay with IC_50_ values of 28.0 and 12.0 μM, respectively,^75^ whereas noraverufanin (36) exhibited a weak HIV latency–reversal activity with reactivation of 43.3% at concentration of 10.0 μM.^73^ Nidurufin (37) which has been also isolated from the marine fungi *A. niger*^70^ as well as *P. purpurogenum* G59,^65^ showed weak antitumor activity against the bone marrow cancer cell line, K562 with an inhibition rate percentage of 25.5% at a concentration of 100.0 μg mL^−1^^65^ and moderate antioxidant capacity with 0.62 as Trolox equivalent as antioxidant.^67^

Another previous study showed that nidurufin (37) had exhibited strong anticancer activity against the A-549 cells, the human ovarian cancer cells, SK-OV-3, the human skin cancer cells, SK-MEL-2, the human CNS cancer cells, XF-498, and the human colon cancer HCT-15 with IC_50_ values of 1.83, 3.39, 3.16, 1.78, and 2.2 μg mL^−1^ beside good antibacterial activity against different strains of the Gram-positive bacteria *Str*. *pyogenes* and *S*. *aureus* with MIC values of equal to or less than 3.13 μg mL^−1^.^68^

Compound 38 (6,8-*O*,*O*′-dimethyl-nidurufin), showed weak antibacterial activity against the Gram-positive *S*. *aureus* as well as the Gram-negative *E. coli* with inhibition zones of 7 and 6.5 mm, respectively using the disk diffusion method at a concentration of 20.0 μg per disk,^69^ suggesting that the new derivatization by *O*,*O*′-dimethylation in position 6 and 8 in this compound had affected the antibacterial activity of the parent metabolite, nidurufin (37) which showed better antibacterial activity when tested against the Gram-positive bacteria as discussed above.

Versiconol (39) exhibited weak anticancer activity against the A-549 cells, the SK-OV-3 cells, the SK-MEL-2 cells, the XF-498 cells, and the HCT-15 cells with IC_50_ values of 20.45, 15.29, 15.86, 23.73, and 19.2 μg mL^−1^, respectively,^68^ whilst its *O*,*O*′-dimethylated derivative, 6,8-*O*,*O*′-dimethyl-versiconol (40) showed selective weak antibacterial activity against *S*. *aureus* with inhibition zones of 6.5 mm using disk diffusion method at a concentration of 20.0 μg per disk when tested against both *S*. *aureus* and *E. coli*.^69^

Other bioactive compounds isolated from the marine fungus *A. versicolor* were compounds 41 and 42, 47 and 48, and 50–54.^59,69,76^ 1-methyl-emodin (41) which is an *O*-methylated derivative of emodin (14) and both were isolated from *A. versicolor*,^59^ exhibited better cytotoxic activity than emodin (14) itself against human epidermoid carcinoma cell line, KBv200 with an IC_50_ value of 190.81 μM,^40^ although 41 did not show any cytotoxicity against the human leukemia cell line, CCRF-CEM and some other solid tumors including the human lung H-125, human colon HCT-116, and human liver Hep-G2 cells.^76^ On the other hand, compound 41 showed less inhibitory activity against Hepatitis C virus (HCV) protease than its parent 14 with IC_50_ values of 40.2 and 22.5 μg mL^−1^, respectively.^76^ The same study showed that the new metabolite from *A. versicolor*; isorhodoptilometrin-1-methyl-ether (42) displayed moderate antibacterial activity against *B. cereus*, *B. subtilis*, and *S*. *aureus* at a concentration of 50.0 μg per disk and mild selective cytotoxicity against the Hep-G2 cell line.^76^

Additionally, 1-hydroxy-2-methyl-anthraquinone (47) and its novel dimethoxy derivative; 2-(dimethoxy methyl)-1-hydroxy-9,10-anthraquinone (48) were evaluated for their antibacterial activity against two strains of methicillin-resistant *S*. *aureus* (MRSA) (CGMCC 1.12409 and ATCC 43300) and three strains of *Vibrio* (*V. rotiferianus*, *V. vulnificus*, and *V. campbellii*). Noteworthy, the dimethoxy derivative 48 was highly active against the MRSA strains showing MIC values of 7.8 and 3.9 μg mL^−1^, respectively, and was moderately active against the *Vibrio* strains with MIC values ranging from 15.6 to 62.5 μg mL^−1^.^59^ The same study mentioned that a molecular docking study was conducted to explain the cause behind this antimicrobial activity revealing the least binding energy of compound 48 with both AmpC β-lactamase and topoisomerase IV (Topo IV).^59^ On the other hand, its parent compound 47 displayed potent larvicidal activity against the larvae of *Aedes aegypti* with an IC_50_ value of 1.8 μg mL^−1^.^77^

Moreover, another anthraquinone derivative, damnacanthal (50) which was reported from *A. versicolor*^59^ exhibited strong larvicidal activity against the larvae of *Aedes aegypti* with an IC_50_ value of 7.4 μg mL^−1^^77^ and weak antibacterial activity against some strains of MRSA and *Vibrio* with MIC values ranging from 31.3 to 125.0 μg mL^−1^.^59^ Similarly, xanthopurpurin (51) showed weak antibacterial properties against some strains of MRSA and *Vibrio* with the same MIC range of damnacanthal (50).^59^ Also, compound 51 previously showed strong antiplatelet aggregation activity *via* inhibition of collagen-induced aggregation.^78^ In addition, a chemically related rubiadin (52) showed a strong inhibitory activity on the formation of advanced glycation end products with an IC_50_ value of 179.31 μM.^79^ Notably, its hydroxylated derivative; 6-hydroxyrubiadin (53) displayed potent inhibitory activity on phosphatase of regenerating liver-3 with an IC_50_ value of 1.3 μg mL^−1^ causing inhibition of migration of phosphatase of regenerating liver-3 expressed tumor cells with no cytotoxicity.^80^

Additional four derivatives 55–58 were isolated from the marine-derived fungus *A. wentii*.^49,81^ Wentiquinone C (55) showed no free radical scavenging activity up to a concentration of 1000.0 μg mL^−1^,^49^ whereas compounds 56–58 were not tested for any relevant bioactivity.^81^

**Fig. 3:**
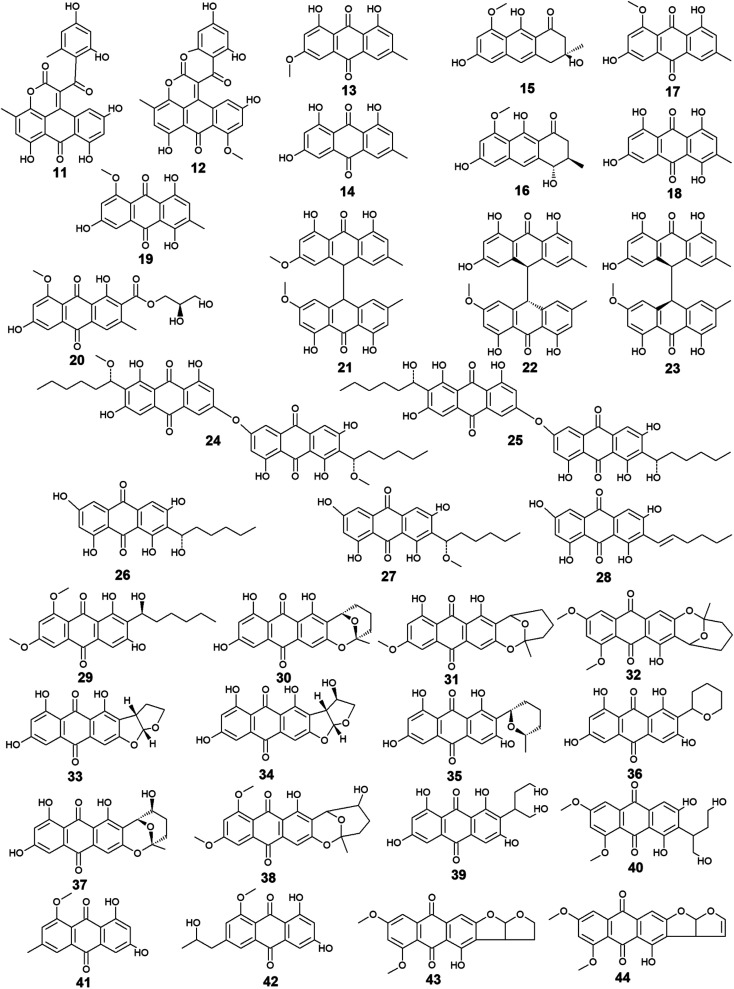
Chemical structures of compounds 11–44.

**Fig. 4:**
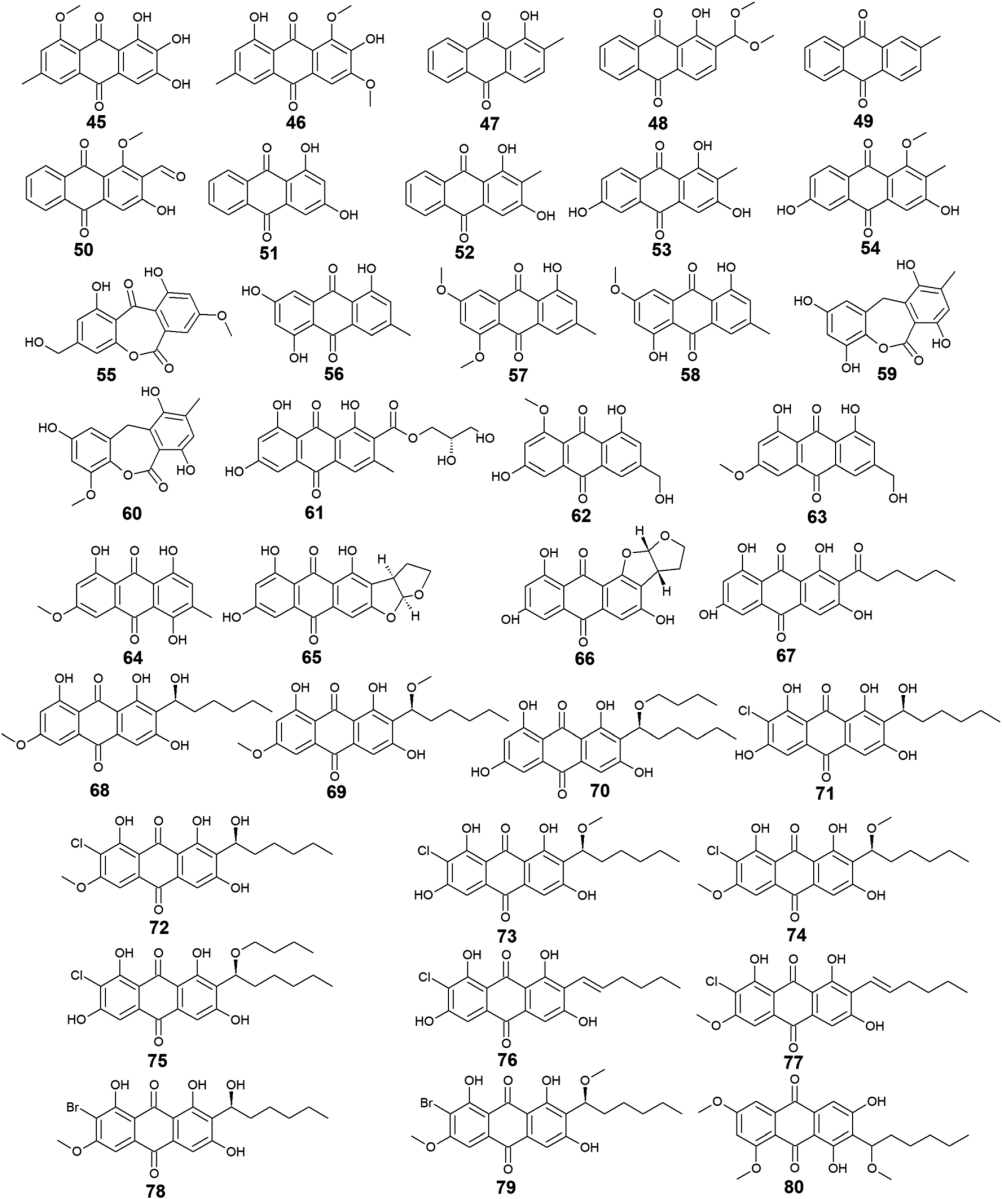
Chemical structures of compounds 45–80.

Further derivatives including compounds 59–64 were isolated from the halotolerant fungus *A. variecolor*,^50^ while compounds 65–67 were reported from *A. nidulans*.^71^ Compounds 59 and 60 exhibited potent DPPH radical scavenging activity (antioxidant activity) with IC_50_ values of 6.0 and 11.0 μM, respectively^50^ suggesting that the *O*-methylation of eurotinone (59), slightly affected its antioxidant activity. Interestingly, Questinol (62) which was also isolated from the marine-derived fungi *Talaromyces stipitatus* KUFA 0207 ^82^ and *Eu*. *amstelodami*,^83^ displayed significant anti-inflammatory activity *via* different mechanisms including inhibition of both nitric oxide and prostaglandin E2 production and, inhibition of the production of some inflammatory cytokines such as interleukin-1β (IL-1β), IL-6, and tumor necrosis factor-α. Compound 62 also showed slight inhibitory activity against cyclooxygenase-2 (COX-2) expression at a concentration of 200.0 μM.^83^ In addition, compound 62 exhibited potent anti-obesity activity with a 60% reduction in the stained lipids with an IC_50_ value of 0.95 μM, while the chemically related compound, fallacinol (63) showed no significant anti-obesity activity.^82^ Interestingly, versicolorin C (65) displayed selective potent antibacterial activity against both *E. coli* and *V. parahaemolyticus* with a MIC value of 1.0 μg mL^−1^ and, against *V. anguillarum* and *Edwardsiella ictaluri* with MIC values of 4.0 and 8.0 μg mL^−1^, respectively, whilst the closely related congener isoversicolorin C (66) displayed selective potent antibacterial activity against both *V. alginolyticus* and *Edwardsiella ictaluri* with MIC values of 1.0 and 4.0 μg mL^−1^, respectively.^71^ Further, twelve anthraquinones including three non-halogenated ones 68–70, seven new chlorinated anthraquinone derivatives 71–77, and two new brominated anthraquinone derivatives 78 and 79 were isolated from the marine-derived fungus *Aspergillus* sp. SCSIO F063,^66^ in addition to compound 80 which was reported from another marine-derived fungus *Aspergillus* sp. SF-6796.^72^ Compounds 68–70 are chemically related to each other and are derivatives of averantin (26) which was isolated in the same study as a metabolite from *Aspergillus* sp. SCSIO F063,^66^ while it was isolated earlier from the marine-derived fungi *A. versicolor*.^64^ Averantin-1′-butyl-ether (70) exhibited weak cytotoxicity against SF-268 and MCF-7 cell lines with IC_50_ values of 47.19 and 40.47 μM, respectively, revealing slightly better cytotoxicity than its parent; averantin (26) which only showed activity against the MCF-7 cell line with an IC_50_ value of 45.47 μM,^66^ suggesting that the structural modification in 70 has improved its bioactivity. By contrast, neither compound 68 nor 69 displayed any cytotoxicity against all tested human cell lines including NCI–H460, SF-268, and MCF-7 ref. 66 indicating that O-methylation of averantin (26) in compounds 68 and 69 may negatively influence their cytotoxicity. It is noteworthy that the chlorinated anthraquinone derivative, 72 exhibited potent cytotoxicity against NCI–H460, SF-268, and MCF-7 cells with IC_50_ values of 7.42, 7.11, and 6.64 μM, respectively. While 71 showed weak cytotoxicity against only the MCF-7 cell line with an IC_50_ value of 36.41 μM, 73 displayed better cytotoxic activity against the three cell lines; NCI–H460, SF-268, and MCF-7 with IC_50_ values of 37.19, 34.06 and 26.09 μM, respectively.^66^ The other chlorinated anthraquinones, 75 and 77 demonstrated weak to modest cytotoxic activity against only the MCF-7 cell line with IC_50_ values of 49.53 and 24.38 μM, respectively. The same study revealed that from the two isolated brominated anthraquinones, only 78 displayed modest cytotoxicity against NCI–H460, SF-268, and MCF-7 cell lines with IC_50_ values of 18.91, 24.69, and 25.62 μM, respectively.^66^ Furthermore, another bioactive derivative of averantin (26) isolated from *Aspergillus* sp. is 6,8,1′-*O*,*O*′,*O*′′-trimethyl-averantin (80) which showed an anti-neuroinflammatory effect *via* different mechanisms including suppression of the overproduction of many pro-inflammatory mediators including COX-2, prostaglandin E2, and nitric oxide in lipopolysaccharide-activated BV2 microglial cells^72^ (Fig. 3 and 4).

### 3.3. Anthraquinones from *Penicillium* sp.

Furthermore, eighteen compounds 81–98 besides the previously reported compounds 14, 17, 26, 27, and 37 were isolated from different species of the marine-derived fungus *Penicillium*. Indeed, penicillanthranin A (81) and B (82) which are anthraquinone-citrinin derivatives, as well as chrysophanol (83) and ω-hydroxyemodin (84), were isolated from the marine fungus *P. citrinum* PSU-F51.^53^ Penicillanthranin A (81) and chrysophanol (83) exhibited selective antibacterial activity against the Gram-positive *S. aureus* ATCC25923 with MIC value of 16.0 μg mL^−1^ for both compounds and MRSA SK1 with MIC values of 16.0 and 64.0 μg mL^−1^, respectively, while compounds 82 and 84 were not screened for their antimicrobial activity in the same study.^53^ Interestingly, some earlier studies revealed that ω-hydroxyemodin (84) showed moderate activity against MRSA SK1 and mild activity against *S*. *aureus* ATCC 25923 with MIC values of 32.0 and 200.0 μg mL^−1^, respectively,^54^ in addition to good anti-mycobacterial activity against *M. tuberculosis* H37Ra with a MIC value of 12.5 μg mL^−1^.^61^ It also showed potent cytotoxicity against the human oral epidermoid carcinoma KB cells with an IC_50_ value of 4.5 μg mL^−1^, and weak cytotoxic activity against both the human breast cancer cells, MCF7 and the human lung carcinoma cells, NCI–H187 with IC_50_ values of 22.0 and 39.0 μg mL^−1^, respectively.^61^ In contrast, penicillanthranin A (81) showed selective cytotoxicity to the KB cell lines with an IC_50_ value of 30.0 μg mL^−1^.^53^

Another bioactive metabolite, 2′-acetoxy-7-chlorocitreorosein (85) which was first recovered from a mangrove-derived fungus *P. citrinum* HL-5126 ref. 84 demonstrated moderate antibacterial activity against *S*. *aureus* and significant activity against *V. parahaemolyticus* with MIC values of 22.8 and 10.0 μg mL^−1^, respectively,^84^ suggesting that such modification in its structure by acetylation, chlorination, and *O*-methylation of ω-hydroxyemodin (84) resulted in significant improvement in its antibacterial activity. Further anthraquinone derivatives discovered from the marine fungus *P. oxalicum*, including citreorosein-3-*O*-sulphate (86), emodin-3-*O*-sulphate (87), and aloe-emodin (88) were not tested for any relevant activity.^85^ However, other previous studies revealed that aloe-emodin (88) displayed modest antimalarial activity against *Plasmodium falciparum* (MRC-2) with an EC_50_ value of 22.0 μg mL^−1^^86^ and weak antimicrobial activity against the Gram-positive bacteria, *S*. *aureus*, *S*. *epidermidis*, *B. cereus*, *B*. *subtilis*, and *Micrococcus kristinae*, and the Gram-negative bacteria, *E*. *coli*, *Enterobacter aerogenes*, *Proteus vulgaris*, and *Shigella sonnei* with MIC values ranging from 62.5 to 250.0 μg mL^−1^.^87^

Additional ten bioactive compounds including eight newly isolated anthraquinone–amino acid conjugates, namely emodacidamide A–H (89–96) along with the previously isolated anthraquinone derivatives; emodic acid (97) and 2-chloro-1,3,8-trihydroxy-6 (hydroxymethyl)-anthracene-9,10 dione (98), were isolated from the marine fungus *Penicillium* sp. SCSIO sof101.^88^ Emodacidamides A–H (89–96) displayed immunomodulatory activity with inhibitory activity against IL-2 production from Jurkat cells.^88^ Intriguingly, emodacidamides A (89), C (91), and E (93) showed potent IL-2 inhibitory activity with IC_50_ values of 4.1, 5.1, and 5.4 μM, respectively.^88^ Meanwhile, emodic acid (97) showed no remarkable inhibition of IL-2 secretion at a concentration of 20.0 μM, indicating that amino acid conjugation with the anthraquinone derivatives enhanced their inhibitory effect on IL-2 secretion.^88^

**Fig. 5:**
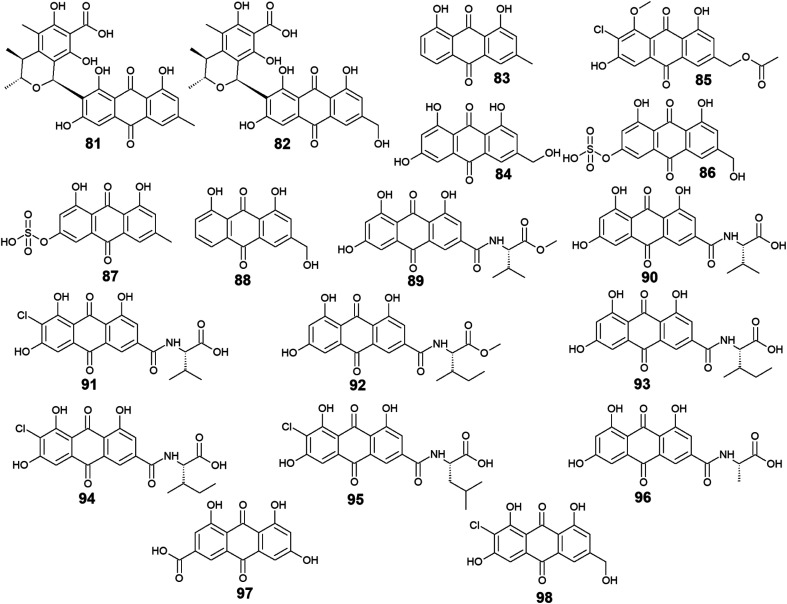
Chemical structures of compounds 81–98.

On the other side, emodic acid (97) which was previously isolated from the marine endophytic fungus *Eu*. *rubrum*,^58^ evoked potent inhibition of p56^lck^ tyrosine kinase with an IC_50_ value of 1.07 μg mL^−1^.^89^ In addition, compound 97 demonstrated a potent inhibitory effect on both the tyrosine kinase domain of the epidermal growth factor receptor and protein tyrosine kinase p59^fyn^ with IC_50_ values of 0.078 and 0.080 μg mL^−1^, respectively without any noted cytotoxicity on human foreskin fibroblast^89^ (Fig. 5).

### 3.4. Anthraquinones from *Stemphylium* sp.

The marine-derived fungus *Stemphylium* is another good source of the bioactive anthraquinones with thirty-two recovered compounds 99–130. A group of twenty-five anthraquinones derivatives 99–123 were reported from a mangrove-derived fungus *Stemphylium* sp. 33 231 ref. 90 including the bioactive altersolanol A, B, C (99, 101, 104) and L (105) as well as their derivatives dihydroaltersolanol A (100), tetrahydroaltersolanol B (102), 2-*O*-acetylaltersolanol B (103).

Altersolanol A (99) showed selective antimicrobial activity against *S*. *aureus*, *E. coli*, *B. subtilis*, and *Micrococcus tetragenus* with MIC values of 2.07, 4.1, 4.1, and 8.2 μM, respectively, whereas altersolanol B (101) displayed similar antibacterial activity against *S*. *aureus*, *E. coli* and *B. subtilis* as well as the Gram-positive bacterium, *Kocuria rhizophila* with MIC values of 7.8 μM for all strains.^90^ The same study revealed that altersolanol C (104) had a narrow spectrum of activity against only *B. subtilis* with a MIC value of 8.8 μM, while altersolanol L (105) had no antibacterial activity against the tested strains.^90^ In the contrast, another recent study demonstrated that altersolanol L(105), had a modest antifungal activity against *P. italicum* and *Rhizoctonia solani* with MIC values of 35.0 and 50.0 μg mL^−1^, respectively.^91^

Additionally, a recent study showed that both altersolanol A (99) and B (101) had strong cytotoxicity against MCF-7 and HCT-116 cell lines with IC_50_ values of [7.21, 1.3 μM] for altersolanol A (99) and, [9.0, 3.5 μM] for altersolanol B (101), respectively.^92^ By contrast, dihydroaltersolanol A (100) did not show any antibacterial activity or cytotoxicity when tested against various microbes and cell lines,^90,93^ suggesting that the derivatization of its parent altersolanol A (99) into dihydroaltersolanol A (100) lead to a significant change in its biological activities.

Furthermore, ampelanol (107), macrosporin (108) and its sulphate derivative, macrosporin-7-*O*-sulphate (109), in addition to its glycosidic derivative, macrosporin 2-*O*-(6′-acetyl)-*α*-d-glucopyranoside (110), as well as auxarthrol C (111), were also recovered from the marine fungus *Stemphylium* sp. 33 231.^90^ Ampelanol (107) displayed moderate cytotoxicity against the murine lymphoma cell line, L5178Y,^94^ whereas macrosporin (108) exhibited significant antibacterial activity against *Micrococcus tetragenus*, *E. coli*, and *S*. *aureus* with MIC values of 4.6, 4.6, and 9.2 μM, respectively.^90^ On the other hand, both derivatives of macrosporin (108), macrosporin-7-*O*-sulphate (109) and macrosporin 2-*O*-(6′-acetyl)-α-d-glucopyranoside (110) displayed no antibacterial activity against the same indicator strains up to a concentration of 10.0 μM,^90^ indicating that these modifications in the chemical structure of macrosporin (108) have greatly affected its antibacterial activity. Additionally, macrosporin (108) was shown to have potent antifungal activity against *Fusarium oxysporum* (*F. oxysporum*) with a MIC value of 3.75 μg mL^−1^ and modest antifungal activity against *Colletotrichum musae*, *F. graminearum*, *P. italicum*, and *Colletotrichum gloeosporioides* with MIC values ranging from 30.0 to 60.0 μg mL^−1^.^91^ Noteworthy, compound 110 demonstrated a remarkable brine shrimp lethality using *Artemia salina* with an LD_50_ value of 10.0 μM,^90^ while the parent compound 108, and its derivative 109 showed no lethality in the same study^90^ suggesting that brine shrimp lethality might be dependent on acetylation and/or glycosylation of this compound. Also, the same study revealed that auxarthrol C (111) displayed selective antibacterial activity against only the Gram-negative organism, *E. coli* with a MIC value of 9.8 μM with no notable cytotoxicity or brine shrimp lethal effect.^90^

Moreover, other bioactive anthraquinone dimers including alterporriols B–E (113–116), N (117), Q (118), U (121), and V (122) were also isolated from the same fungus *Stemphylium* sp. 33 231.^90^ The anthraquinone dimers, alterporriols B–E (113–116) displayed positive antibacterial activity, whereas alterporriol A (112) did not show either antibacterial or cytotoxic activity.^90^ Alterporriol B (113) showed a narrow spectrum of antimicrobial activity against *B. cereus* with a MIC value of 7.9 μM, whereas alterporriol C (114) showed selective antibacterial activity against *S*. *albus* with a MIC value of 8.9 μM. Interestingly, alterporriol D (115) exhibited notable antibacterial activity against both *S*. *aureus* and *E. coli* and with MIC values of 5.0 and 7.5 μM, respectively, while alterporriol E (116) displayed potent antimicrobial activity against both *B. cereus* and *E. coli* with MIC values of 2.5 and 5.0 μM, respectively.^90^ The same study demonstrated that alterporriol Q (118) and R (119) showed no antimicrobial activity against various tested microbes up to a concentration of 10.0 μM.^90^ This finding was confirmed in another study which showed that both compounds did not display any antibacterial activity against different Gram-positive bacteria as well as *E. coli* from the Gram-negative bacteria up to a concentration of 20.0 μM.^93^ However, alterporriol Q (118) exhibited strong antiviral activity against the porcine reproductive and respiratory syndrome virus with a MIC value of 22.0 μM, whereas alterporriol R (119) showed no antiviral activity.^93^ Also, the same study revealed that alterporriol C (114) had a modest antiviral activity with a MIC value of 39.0 μM.^93^ In addition, the other anthraquinone dimers, alterporriol U (121) and V (122) exhibited a narrow spectrum of antibacterial bioactivity against the Gram-positive bacterium, *B. cereus* with MIC values of 8.3 and 8.1 μM, respectively.^90^

**Fig. 6:**
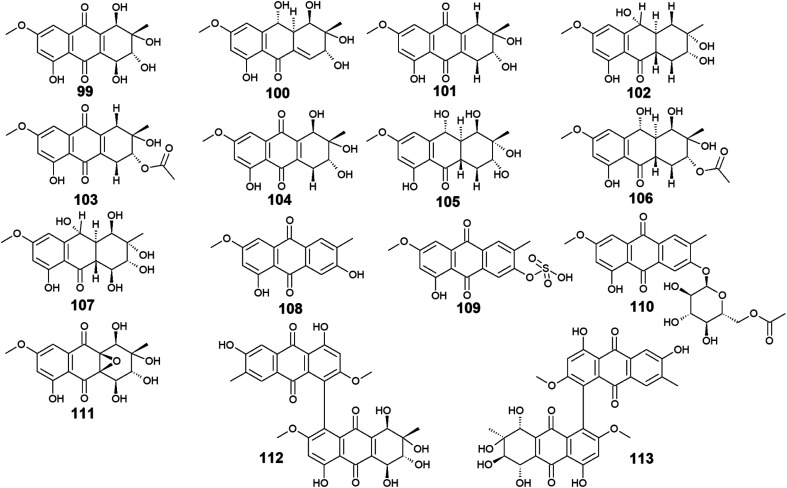
Chemical structures of compounds 99–113.

**Fig. 7:**
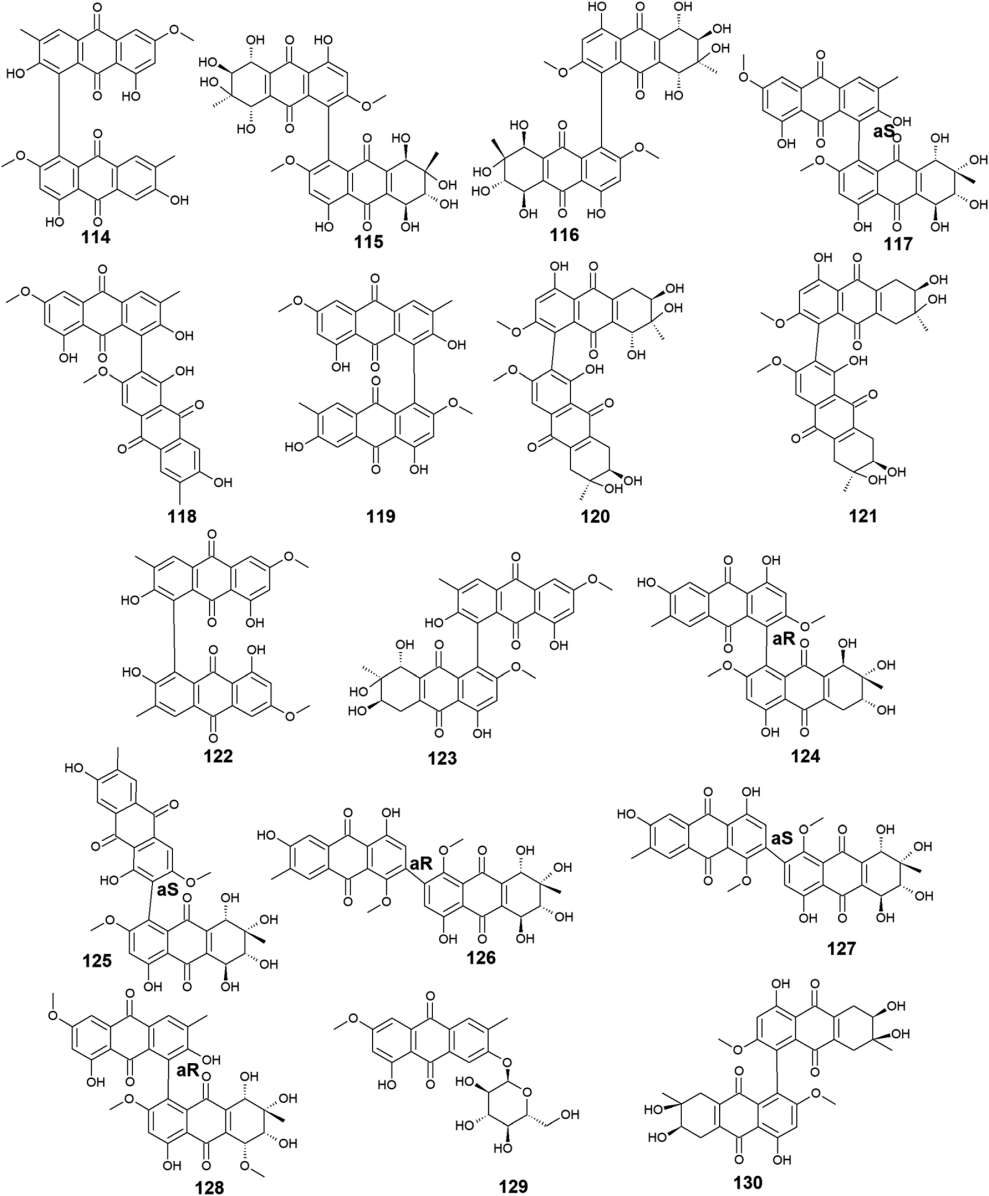
Chemical structures of compounds 114–130.

Further anthraquinone dimers including alterporriol N (117), F (124), G (125), Z1 (126), Z2 (127), and Z3 (128) were also isolated recently from another marine fungus *Stemphylium* sp. FJJ006.^95^ They showed neither antimicrobial activity against the Gram-positive and Gram-negative bacterial strains up to a concentration of 128.0 μg mL^−1^ nor antitumor activity against a panel of cancer cell lines with an IC_50_ value higher than 20.0 μM. Also, they did not show bioactivity against the microbial enzymes, isocitrate lyase, and sortase A with an IC_50_ value of more than 145.0 μM. However, the same study revealed that alterporriols N (117), F, G, and Z_1_–Z_2_ (124–127) had anti-inflammatory activity through their capability of suppressing the lipopolysaccharide-induced nitric oxide production in the murine macrophages RAW 264.7 cells with IC_50_ values of 8.4, 9.6, 10.7, 11.6, and 16.1 μM, respectively, whereas alterporriol Z_3_ (128) did not display any anti-inflammatory activity.^95^ On the other hand, another previous study demonstrated the potent cytotoxicity of alterporriol F (124) against the HeLa and KB human cell lines with IC_50_ values of 6.5 and 7.0 μg mL^−1^, respectively.^96^ In addition, alterporriol N (117) was presented in another study as a weak antimicrobial agent with a narrow spectrum of activity against only the Gram-positive bacteria, *Enterococcus faecalis*, MRSA, and *Str*. *pneumoniae* with MIC values of 15.63, 62.5, and 125.0 μg mL^−1^, respectively, while the same study revealed that alterporriol G (125) had a moderate cytotoxicity against the mouse cancer cell line, L5178Y^97^ (Fig. 6 and 7).

### 3.5. Anthraquinones from *Alternaria* sp.

A list of twenty anthraquinones was isolated earlier from different species of *Alternaria* including the previously mentioned compounds, 100–102, 104, 105, 107, 108, and 114 as well as twelve anthraquinone derivatives, 131–142. Two bioactive bi-anthraquinones, named alterporriol K (131) and L (132) were isolated from the marine endophytic fungus *Alternaria* sp. ZJ9-6B ref. 98 and displayed moderate cytotoxic activity against the human breast cancer cells, MCF-7 and MDA-MB-435 with IC_50_ values of [29.11 and 26.97 μM] for alterporriol K (131) and [20.04 and 13.11 μM] for alterporriol L (132), respectively, while alterporriol M (133) was not evaluated for any biological activity in this study.^98^

Further compounds including alterporriol O (134) and P (135) were isolated from the marine-derived *Aspergillus* sp. ZJ-2008003. Only alterporriol P (135) exhibited significant cytotoxicity against the human prostate cancer cell line, PC3, colon cancer cell line, HCT-116, liver hepatoma cell lines, Hep-G2 and Hep-3B in addition to the breast cancer cell line, MCF-7/ADR with IC_50_ values of 6.4, 8.6, 20.0, 21.0, and 23.0 μM, respectively. Unlikely, alterporriol O (134) did not demonstrate any bioactivity when it was evaluated for its cytotoxicity, antibacterial activity, and antiviral activities.^93^

Additional anthraquinones, tetrahydroaltersolanols C–F (136-139) were also isolated from the marine-derived *Alternaria* sp. ZJ-2008003.^93^ Only tetrahydroaltersolanol C (136) displayed moderate antiviral activity against the porcine reproductive and respiratory syndrome virus with an IC_50_ value of 65.0 μM.^93^

**Fig. 8:**
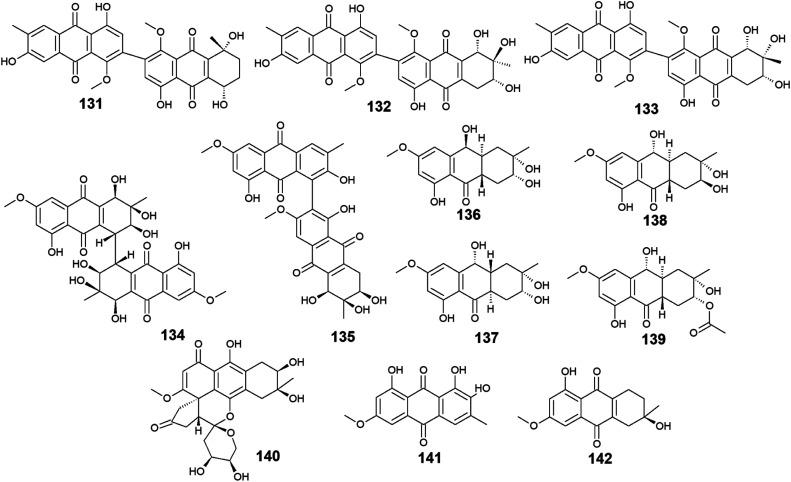
Chemical structures of compounds 131–142.

More anthraquinone derivatives 140–142 were reported recently from the marine fungus *Alternaria tenuissima* DFFSCS013.^99^ Anthrininone A (140) demonstrated selective protein tyrosine phosphatase inhibitory effect on indoleamine 2,3 dioxygenase 1 enzyme with an IC_50_ value of 32.3 μM as well as the stimulatory effect on the intracellular levels of calcium in HEK293 cells at a concentration of 10.0 μM.^99^ It is noteworthy that 6-*O*-methyl-alaternin (141) displayed a wide range of anti-protein tyrosine phosphatases activity including activity against TCPTP, SHP1, SHP2, and PTP-MEG2 enzymes with potent bioactivity against both indoleamine 2,3 dioxygenase 1 enzyme and PTP1B with IC_50_ values of 1.7 and 2.1 μM, respectively. On the other hand, compound 141 did not show a noticeable stimulatory effect on the intracellular levels of calcium in HEK293 cells at a concentration of 10.0 μM ref. 99 (Fig. 8).

### 3.6. Anthraquinones from *Trichoderma* sp.

*Trichoderma* sp. is another prolific anthraquinones producer from which the previously discussed compounds 14, 83, and 84 were isolated as well as the anthraquinone derivatives, 143–155. Harzianumnones A and B (143 and 144) were reported earlier as new hydroxyanthraquinones from the marine fungus *T*. *harzianum* XS-20090075.^100^ They showed neither DNA Topo I inhibitory activity nor anti-acetylcholinesterase activity.^100^ The same study revealed that phomarin (145), ω-hydroxydigitoemodin (146), pachybasin (147), and (+)-2′*S*-isorhodoptilometrin (148) isolated also from *T*. *harzianum* XS-20090075, displayed a weak anti-acetylcholinesterase activity at a concentration of 100.0 μM.^100^

Interestingly, pachybasin (147) also demonstrated potent cytotoxic activity against the human cancer cell lines, KB and KBv200 with IC_50_ values of 3.17 and 3.21 μM, respectively.^40^ In addition, its derivative, ω-hydroxypachybasin (149) as well as (+)-2′*S*-isorhodoptilometrin (148) exhibited moderate or good cytotoxicity against Hep-G2 and HeLa cancer cell lines showing IC_50_ values of [9.39 and 22.6 μM] for ω-hydroxypachybasin (149) and [2.10 and 8.59 μM] for (+)-2′*S*-isorhodoptilometrin (148), respectively, whilst only ω-hydroxypachybasin (149) exhibited cytotoxicity against the colon cancer cells, HCT-116 with an IC_50_ value of 29.8 μM.^100^ Also, compounds 148 and 149 revealed moderate DNA Topo I inhibitory activity with IC_50_ values of 100.0 and 50.0 μM, respectively, in addition to moderate selective antibacterial activity against the Gram-positive bacterium, *S*. *aureus* showing MIC value of 25.0 μM for both compounds.^100^

Moreover, another study demonstrated that compound 148 isolated from the marine-derived fungus *T*. *aureoviride* PSU-F95 showed good antibacterial activity against MRSA with a MIC value of 16.0 μg mL.^54^ Similarly, coniothranthraquinone 1 (150) displayed significant antibacterial activity against MRSA and *S*. *aureus* with MIC values of 8.0 and 16 μg mL^−1^, respectively.^54^ In the contrast, trichodermaquinone (151) which was also isolated from the marine fungus *T. aureoviride* PSU-F95 demonstrated very weak antibacterial activity against MRSA with a MIC value of 200.0 μg mL^−1^.^54^ However, compounds 152 and 153 which were recovered also from the marine fungus *T. aureoviride* PSU-F95, both were not evaluated for any bioactivity in this study.^54^

**Fig. 9:**
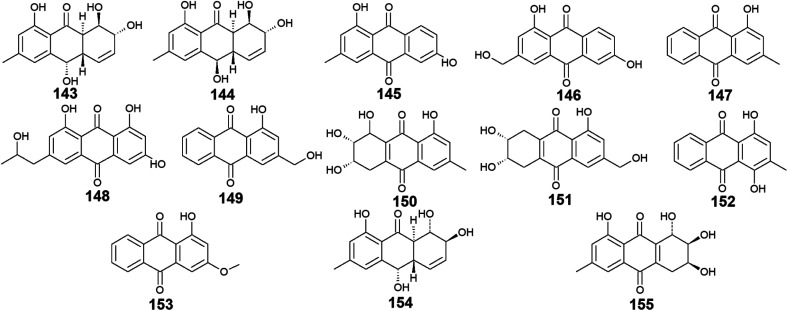
Chemical structures of compounds 143–155.

Additionally, coniothyrinone A (154) and lentisone (155) were previously isolated from another marine fungus, *Trichoderma* sp., and exhibited potent antibacterial activity against the Gram-negative bacteria, *V. parahaemolyticus*, *V. anguillarum*, and *Pseudomonas putida* with MIC values of [6.25, 1.56, 3.13 μM] for coniothyrinone A (154) and [12.5, 1.56, 6.25 μM] for lentisone (155), respectively^60^ (Fig. 9).

### 3.7. Anthraquinones from *Eurotium* sp.

Seventeen anthraquinones and their derivatives were reported from species of the marine fungus *Eurotium*, including the previously mentioned compounds 14, 15, 18–20, 60, 62, 97, and 154 in addition to other eight congeners, 156–163. Compound 9-dehydroxyeurotinone (156) and its *O*-methyl derivative, 2-*O*-methyl-9-dehydroxyeurotinone (157) as well as its glycosidic derivative, 2-*O*-methyl-4-*O*-(α-d-ribofuranosyl)-9-dehydroxyeurotinone (158) were isolated from the marine-derived fungus *Eu*. *rubrum*.^58,62^ The parent compound, 9-dehydroxyeurotinone (156) exhibited weak antibacterial activity against the Gram-negative bacterium, *E. coli* showing a 7 mm zone of inhibition using 100.0 μg per disk. Also, it displayed selective cytotoxic activity against the human cholangiocarcinoma cells, SW1990 with an IC_50_ value of 25.0 μg mL^−1^.^58^ Another study revealed that compounds 157–159 had positive antioxidant activity through free radical scavenging activity against DPPH.^62^

Furthermore, the same study showed that eurorubrin (160) demonstrated a potent free radical scavenging activity with an IC_50_ value of 44.0 μM with better antioxidant activity than the standard antioxidant, butylated hydroxytoluene which had an IC_50_ value of 82.6 μM.^62^ Interestingly, 3-*O*-(α-d-ribofuranosyl)-questin (159) and eurorubrin (160) were re-isolated also from the marine endophytic fungus *Eu*. *cristatum* EN 220. They displayed modest antibacterial activity against the Gram-negative bacterium, *E. coli* with MIC values of 32.0 and 64.0 μg mL^−1^, respectively.^101^ Notably, 3-*O*-(α-d-ribofuranosyl)questinol (161) which is an alcoholic derivative of the bioactive compound, 3-*O*-(α-d-ribofuranosyl)questin (159) showed no antibacterial activity against *E. coli* suggesting that this hydroxylation leads to loss of the antimicrobial activity.^101^

**Fig. 10:**
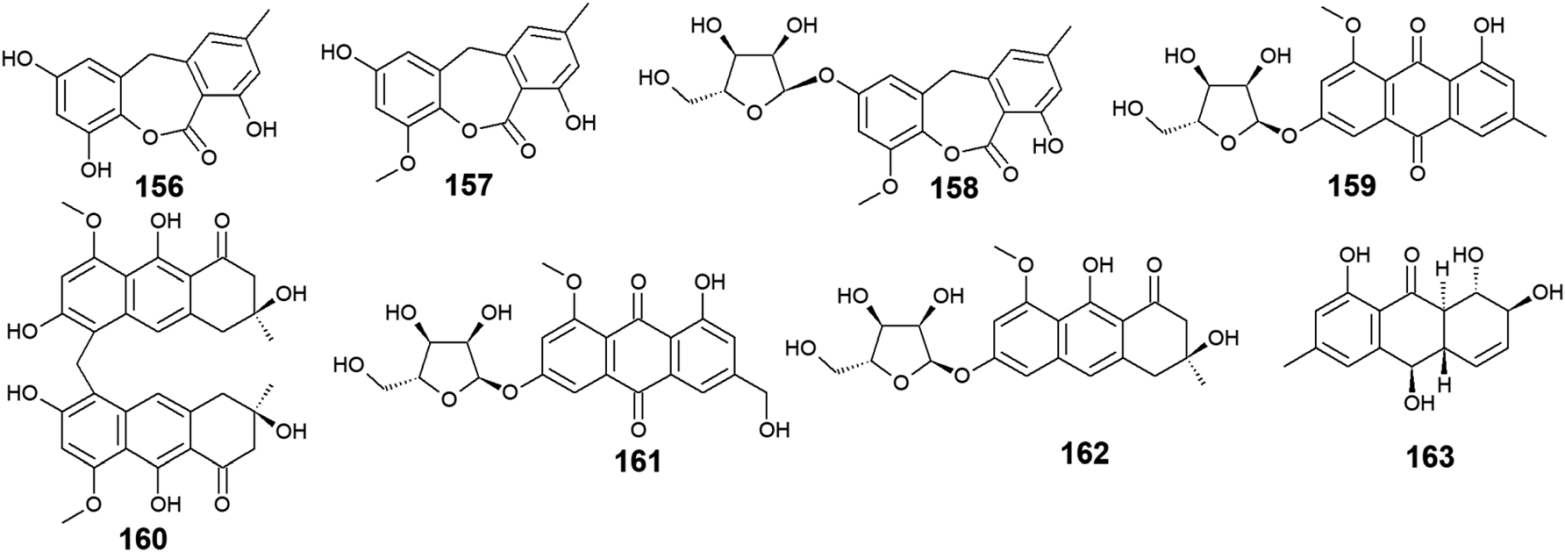
Chemical structures of compounds 156–163.

Furthermore, asperflavin ribofuranoside (162) which was isolated earlier from the marine fungus *Eu*. *cristatum* EN 220 ref. 101 and the marine-derived fungus *Microsporum* sp.,^102^ was reported as a potent free radical scavenging agent with an IC_50_ value of 14.2 μM with better antioxidant activity than the standard antioxidant, ascorbic acid which had an IC_50_ value of 20.0 μM.^102^ Also, it exhibited modest antibacterial activity against both MRSA and the multidrug-resistant *S*. *aureus* with MIC values of 50.0 and 50.0 μg mL^−1^, respectively.^102^ Moreover, rubrumol (163) was reported as a new anthraquinone derivative from the saline-alkali endophytic fungus *Eu*. *rubrum* with relaxation activity on Topo I with an IC_50_ value of 23.0 μM^103^ (Fig. 10).

### 3.8. Anthraquinones from *Fusarium* sp.

Twelve anthraquinone derivatives were isolated earlier from different species of the marine-derived fungus *Fusarium* sp. including the previously discussed compounds 5–8 and 10 along with other structurally related compounds 164–170. Although both nigrosporin A (164) and fusaranthraquinone (165) were recovered from the marine-derived fungus *Fusarium* sp. PSU-F14 ,^39^ only nigrosporin A (164) displayed promising inhibitory activity against photosynthesis and weak antibacterial activity against *B. subtilis* showing an inhibition zone of 14 mm at 200 ppm,^44^ whereas fusaranthraquinone (165) did not demonstrate any antibacterial activity when it was tested against both *S*. *aureus* and MRSA.^39^ Interestingly, additional bioactive fusaquinons A–C (166–168) were reported from the marine fungus *Fusarium* sp. ZH-210 and displayed weak cytotoxic activity against MCF-7, KB, and KBv200 cell lines with IC_50_ values of more than 50.0 μM.^104^

**Fig. 11:**
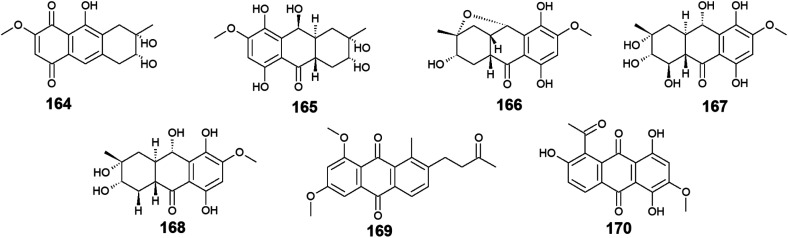
Chemical structures of compounds 164–170.

It is noteworthy that nigrosporin A (164) and fusaquinon A (166) were also evaluated in another study for their antimalarial, anti-mycobacterial, antibacterial, and cytotoxic activity. Both compounds showed no antimalarial, antibacterial, or anti-mycobacterial activity, whereas they showed selective cytotoxicity.^105^ Nigrosporin A (164) displayed weak cytotoxic activity against the MCF-7 cell line with an IC_50_ value of 110.36 μM and good cytotoxicity against the NCI–H187 cell line with an IC_50_ value of 13.69 μM, while fusaquinon A (166) exhibited weak cytotoxicity against both human cancer cells, MCF-7, and monkey kidney cells, Vero cells with IC_50_ values of 84.38 and 44.46 μM, respectively. Also, fusaquinon A (166) displayed potent cytotoxicity against the NCI–H187 cell line with an IC_50_ value of 7.32 μM.^105^ Another bioactive anthraquinone derivative isolated from the mangrove-derived fungus *Fusarium* sp. ZZF60 was 6,8-dimethoxy-1-methyl-2-(3-oxobutyl)anthracene-9,10-dione (169).^106^ Notably, it demonstrated moderate cytotoxicity against Hep2 and Hep-G2 cells with IC_50_ values of 16.00 and 23.00, respectively (Fig. 11).

### 3.9. Anthraquinones from *Engyodontium album*

Six compounds 171–176 out of seven anthraquinone derivatives 171–177 isolated from the marine-derived fungus *Engyodontium album* LF069 were bioactive, while the anthraquinone derivative, Engyodontochone D (177) was not tested for any relevant biological activity.^23^ It is noteworthy that compounds 171–173 exhibited diverse bioactivities including antibacterial, antifungal, and cytotoxic activity. They demonstrated better antibacterial activity against *S*. *epidermidis* and MRSA than chloramphenicol with an IC_50_ values of [0.19 and 0.17 μM] for engyodontochone A (171), [0.21 and 0.25 μM] for JBIR-99 (172), and [0.22 and 0.24 μM] for engyodontochone B (173), respectively.^23^ On the other hand, they displayed weak to modest antifungal activity against the fungi, *C. albicans*, and *T. rubrum* with IC_50_ values ranging from 4.3 to 13.5 μM. Additionally, compounds 171–173 exhibited moderate cytotoxicity against the mouse fibroblasts cell line, NIH-3T3 with IC_50_ values of 11.0, 13.2, and 14.4 μM, respectively.^23^

**Fig. 12:**
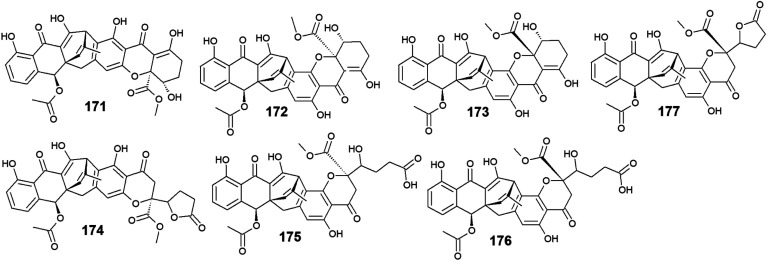
Chemical structures of compounds 171–177.

In addition, engyodontochone C (174) in the same study showed a good selective bioactivity against *S*. *epidermidis* and MRSA with IC_50_ values of 1.80 and 2.39 μM, respectively. In addition, it displayed weak cytotoxic activity against the cell line, NIH-3T3 with an IC_50_ value of 34.3 μM, whereas it did not show any antifungal activity against either, *C. albicans* or *T. rubrum* up to a concentration of 100.0 μM.^23^ Similarly, engyodontochone F (175) demonstrated promising selective antibacterial activity against both *S*. *epidermidis* and MRSA with IC_50_ values of 3.41 and 3.13 μM, respectively although it exhibited weak selective antifungal activity against *T. rubrum* with an IC_50_ value of 73.4 μM. In the contrast, engyodontochone E (176) has only showed potent antibacterial activity against *S. epidermidis* and MRSA with IC_50_ values of 6.77 and 6.74 μM, respectively with no antifungal or cytotoxic activity up to a concentration of 100.0 and 50.0 μM, respectively^23^ (Fig. 12).

### 3.10. Anthraquinones from *Sporendonema casei*

Seven bioactive anthraquinones named 4-dehydroxyaltersolanol A (178) and auxarthrols D–H (179–183) along with the previously discussed altersolanol B (101) were recovered from the marine fungus, *Sporendonema casei* HDN16-802.^107^ This group of anthraquinone derivatives 178–183 were evaluated for their antibacterial activity against *M. phlei*, *B*. *subtilis*, *V*. *parahaemolyticus*, *E*. *coli*, *Pseudomonas aeruginosa*, and *Proteus* sp. and for their antifungal activity against *C. albicans*. Interestingly, 4-dehydroxyaltersolanol A (178) exhibited the best antibacterial activity among this group of anthraquinones against *M. phlei*, *B*. *subtilis*, *Pseudomonas aeruginosa*, *V*. *parahaemolyticus*, and *Proteus* sp. with MIC values ranging from 25.0 to 50.0 μM.^107^ However, its parent altersolanol A (99) demonstrated potent antibacterial activity against *S*. *aureus*, *E. coli*, *B. subtilis*, and *Micrococcus tetragenus* with MIC values of 2.07, 4.1, 4.1, and 8.2 μM,^90^ suggesting that its dehydroxylation might lead to a decrease in its antimicrobial activity.

In the contrast, auxarthrol E (180) and H (183) showed no antimicrobial activity against different indicator strains. However, auxarthrol F (181) only displayed very weak activity against *M. phlei*, *B*. *subtilis*, *Pseudomonas aeruginosa*, and *Proteus* sp. with a MIC value of 200.0 μM. Both auxarthrol D (179) and G (182) demonstrated a broad spectrum of antibacterial activity against *M. phlei*, *B*. *subtilis*, *Pseudomonas aeruginosa*, *V*. *parahaemolyticus*, and *Proteus* sp. with MIC values ranging from 25.0 to 100.0 μM, whereas compound 182 displayed very weak antifungal activity against *C. albicans* with a MIC value of 200.0 μM.^107^

**Fig. 13:**
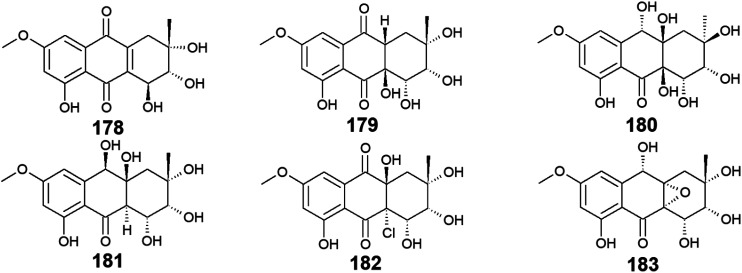
Chemical structures of compounds 178–183.

Moreover, only compounds 179 and 181 were evaluated for their cytotoxicity against different cancer cell lines in the same study revealing modest cytotoxic activity against several cell lines. Compound 179 exhibited a selective cytotoxic effect on seven cell lines including HL-60, HCT-116, MGC-803, MDA-MB-231, SH-SY5Y, PC-3, and BEL-7402 with IC_50_ values ranging from 7.5 to 22.9 μM. In the contrast, compound 181 displayed a broad spectrum of cytotoxicity against the eleven tested cancer cell lines in this study with IC_50_ values ranging from 4.5 to 22.2 μM.^107^ In addition, all compounds 178–183 showed significant anticoagulant activity, meanwhile, they did not show any anti-mycobacterial activity^107^ (Fig. 13).

### 3.11. Anthraquinones from other marine fungi

A considerable number of anthraquinones and their derivatives were isolated from other marine-derived fungi including compounds 184–208. Compounds 184–192, as well as previously discussed anthraquinone derivatives, 5, 41, 83, and 147, were reported from the mangrove endophytes, *Halorosellinia* sp. No. 1403 and *Guignardia* sp. No. 4382.^40^ Eight compounds from them, 184–191 showed weak cytotoxic activity, while 192 displayed no cytotoxicity up to a concentration of 500.0 μM.^40^ It is noteworthy that compounds 184–188 exhibited weak cytotoxicity against both tested cancer cell lines, KB and KBv200 with IC_50_ values ranging from 34.64 to 243.69 μM, whereas compounds 189–191 demonstrated a narrow spectrum of activity against only KBv200 cell line with IC_50_ values of 72.60, 185.68, and 301.47 μM, respectively. The best cytotoxicity was recorded for 1,3-dihydroxy-6-methoxy-8-methyl-anthracene-9,10-dione (187) which displayed activity against both KB and KBv200 cells lines with IC_50_ values of 38.05 and 34.64 μM, respectively.^40^

Interestingly, SZ-685C (193) was isolated as a novel anthraquinone derivative from the marine endophytic fungus *Halorosellinia* sp. No. 1403 with anticancer potential.^108–110^ It was demonstrated that SZ-685C (193) had anticancer activity against the rat pituitary adenoma (MMQ) and human non-functioning pituitary adenoma cell lines with IC_50_ values of 14.51 and 18.76 μM, respectively, while it had an IC_50_ value of 56.09 μM against the normal cell line, rat pituitary cells.^108^ Another study revealed similar results of its cytotoxic activity against the MMQ and normal rat pituitary cell lines with IC_50_ values of 13.2 and 49.1 μM, respectively.^110^ Also, it showed good cytotoxicity against both human MCF-7 and MCF-7/ADR cancer cell lines with IC_50_ values of 7.38 and 4.17 μM, respectively.^109^

Additional anthraquinone derivatives, phomopsanthraquinone (194), and 1-hydroxy-3-methoxy-6-methyl-anthraquinone (195) were isolated from the marine-derived fungus, *Phomopsis* sp. PSU-MA214, besides the previously mentioned compounds 102, 107, 108, and 136.^111^ Phomopsanthraquinone (194) demonstrated cytotoxicity against MCF-7 and KB cancer cell lines with an IC_50_ value of 27.0 μg mL^−1^ for both cell lines. Also, it exhibited moderate antibacterial activity against both MRSA and *S*. *aureus* with MIC values of 64.0 and 128.0 μg mL^−1^, respectively. In the contrast, 1-hydroxy-3-methoxy-6-methyl-anthraquinone (195) neither showed antibacterial activity nor cytotoxicity.^111^

Further three anthraquinones, tetrahydroxyanthraquinone (196), methoxy-tetrahydroxyanthraquinone (197), and 1,2,3,6,8-pentahydroxy-7-[(1 R)-1-methoxyethyl]-9,10-anthraquinone (198) along with previously mentioned noraverufanin (36), were recorded from the sponge-associated fungus *Microsphaeropsis* sp.^112^ All those anthraquinones showed a broad spectrum of protein kinases' inhibitory activity against cyclin-dependent kinase 4 in complex with its activator cyclin D1, protein kinase C, and epidermal growth factor receptor with IC_50_ values ranging from 18.5 to 54.0 μM.^112^

Moreover, the anthraquinone, lunatin (199), and the anthraquinone dimer, cytoskyrin A (200) were reported earlier from the sponge-associated fungus *Curvularia lunata* with positive antibacterial activity.^113^ Both compounds exhibited antibacterial activity against *B*. *subtilis*, *S*. *aureus*, and *E*. *coli* using the disk diffusion method at a concentration of 5.0 μg per disk. Meanwhile, they showed no antifungal activity against *C*. *albicans* up to a concentration of 10.0 μg per disk.^113^

Furthermore, rheoemodin (201), 2, 2′-bis-(7-methyl-1,4,5-trihydroxy-anthracene-9,10-dione) (202), as well as the previously discussed compounds 62, 63, and 84, were isolated earlier from another sponge-associated fungus *Talaromyces stipitatus* KUFA 0207.^82^ Rheoemodin (201) displayed no significant anti-obesity activity, whereas 2, 2′-bis-(7-methyl-1,4,5-trihydroxy-anthracene-9,10-dione) (202) was not tested for any relevant activity.^82^

Additional two anthraquinones, 7-methoxymacrosporin (203) and 7-(γ,γ)-dimethyl-allyloxy-macrosporin (204) along with the previously discussed compounds 102, 105, 107 and 108, were isolated from the mangrove fungus, *Phoma* sp. L28.^91^ 7-methoxymacrosporin (203) displayed weak antifungal activity against *F. graminearum*, *F. oxysporum*, *P. italicum*, *Rhizoctonia solani*, and *Colletotrichum gloeosporioides* with MIC values of 100.0, 100.0, 100.0, 150.0, and 200.0 μg mL^−1^, respectively. Also, 7-(γ,γ)-dimethyl-allyloxy-macrosporin (204) demonstrated weak selective antifungal activity against *F. graminearum*, *Rhizoctonia solani*, and *Colletotrichum gloeosporioides* with MIC values of 80.0, 150.0, and 200.0 μg mL^−1^, respectively.^91^ By comparing this weak antifungal activity of 203 and 204 to their parent macrosporin (108) which displayed potent antifungal activity against *F*. *oxysporum* and modest antifungal activity against *Colletotrichum musae*, *F*. *graminearum*, *P. italicum*, and *Colletotrichum gloeosporioides*,^91^ we can conclude that the structural modifications in both 203 and 204 have greatly affected their bioactivity.

Four additional bioactive anthraquinone derivatives were reported from the marine-derived fungus *Monodictys* sp. including the previously discussed compounds 14, 83, and 147 as well as monodictyquinone A (205). Compound 205 displayed promising antimicrobial activity against *B*. *subtilis*, *E*. *coli*, and *C*. *albicans* showing zones of inhibition with a diameter of 15.0, 15.0, and 11.0 mm, respectively at a concentration of 10.0 μg per disk.^55^

Two other anthraquinone derivatives, 1,3,6-trihydroxy-7-(1-hydroxyethyl) anthracene-9,10-dione (206) and phaseolorin I (207) were isolated earlier from the marine-derived fungi, *Cladosporium* sp. HNWSW-1 ref. 114 and *Diaporthe phaseolorum* FS431,^115^ respectively. Phaseolorin I (207) was inactive when it was tested for its cytotoxicity against the cell lines, MCF-7, Hep-G2, A549, and SF-268,^115^ whereas compound 206 did not demonstrate cytotoxicity against the cell lines, BEL-7042, HeLa, and K562 as well as the human papillomavirus-related endocervical adenocarcinoma SGC-7901 cell lines.^114^ However, anthraquinone 206 exhibited α-glycosidase inhibitory activity with an IC_50_ value of 49.3 μM compared to the standard agent, acarbose which had an IC_50_ value of 275.7 μM.^114^

**Fig. 14:**
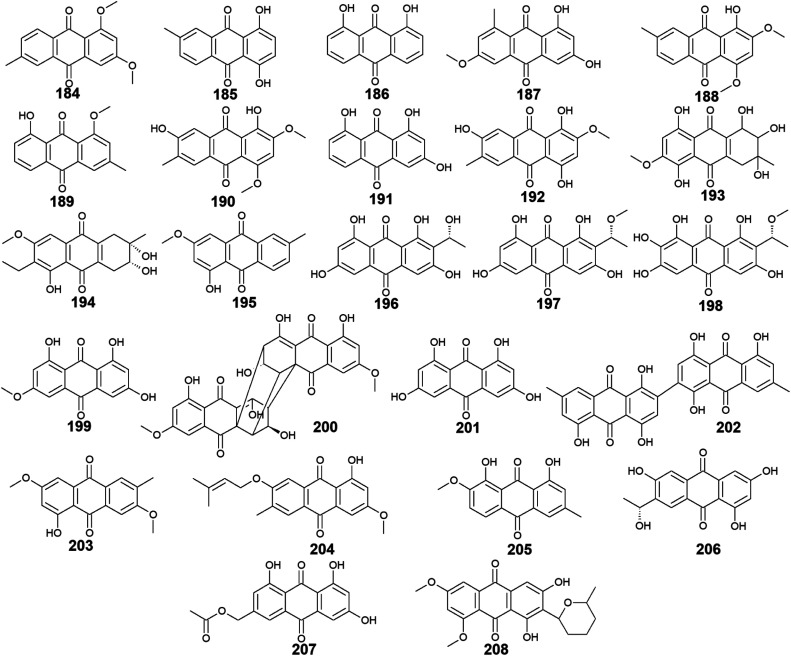
Chemical structures of compounds 184–208.

Finally, 6,8-*O*,*O*′-dimethyl-averufanin (208) which is a derivative of the bioactive anthraquinone derivative, averufanin (35) was previously reported from the unidentified marine endophytic fungus ZSUH-36 as well as the previously mentioned compounds 27, 30, 32 and 33, 40, 43, and 80.^116^ Compound 208 demonstrated weak antifungal activity against the phytopathogenic fungi, *Botrytis cinerea* and *Magnaporthe oryzae* with MIC values of 50.0 and 100.0 μM, respectively.^117^ Also, it displayed good phytotoxicity on the hypocotyls of radish seedlings at a concentration of 100.0 μM with an inhibition rate of 30.6% compared to 28.1% for the standard, glyphosate^117^ (Fig. 14).

## 4 Drug likeness and pharmacokinetics of marine anthraquinones

**Fig. 15:**
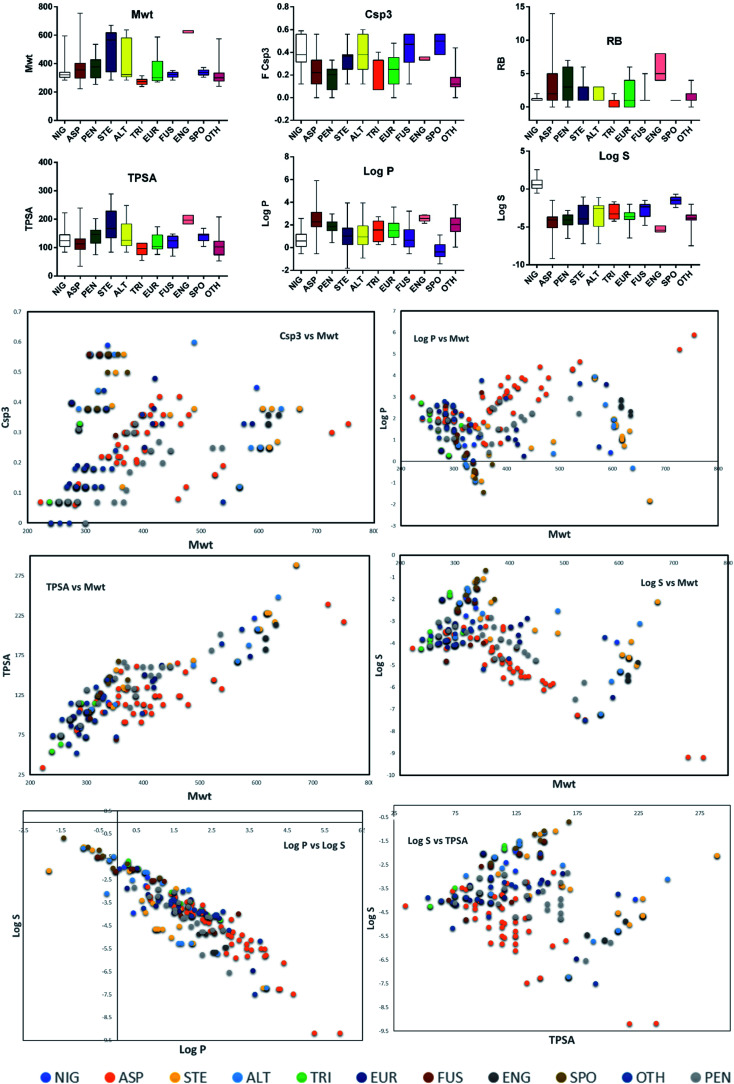
Distribution of molecular weight (*M*_wt_), fraction of sp^3^ carbons (FCsp^3^), number of rotatable bonds (RB), topological polar surface area (TPSA), lipophilicity (log *P*), solubility (log *S*) according to the species. Comparison between the values of FCsp^3^ and *M*_wt_, log *P* and *M*_wt_, TPSA and *M*_wt_, log *S* and *M*_wt_, log *P* and log *S*, and log *S* and TPSA. NIG: *Nigrospora* sp., ASP: *Aspergillus* sp., PEN: *Penicillium* sp., STE: *Stemphylium* sp., ALT: *Alternaria* sp., TRI: *Trichoderma* sp., EUR: *Eurotium* sp., FUS: *Fusarium* sp., ENG: *Engyodontium album*, SPO*: Sporendonema casei*, and OTH: other marine fungi.

**Fig. 16:**
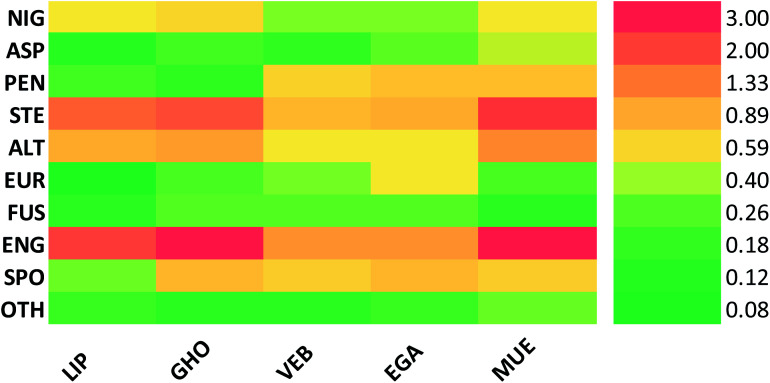
Heatmap of the compliance with rules of drug-likeness according to the classes. LIP: Lipinski, GHO: Ghoose, VEB: Veber, EGA: Agan and MUE: Muegge. NIG: *Nigrospora* sp., ASP: *Aspergillus* sp., PEN: *Penicillium* sp., STE: *Stemphylium* sp., ALT: *Alternaria* sp., TRI: *Trichoderma* sp., EUR: *Eurotium* sp., FUS: *Fusarium* sp., ENG: *Engyodontium album*, SPO*: Sporendonema casei*, and OTH: other marine fungi.

Altogether, 208 anthraquinones and their derivatives were characterized from 20 marine-derived fungal genera. These include *Nigrospora*, *Aspergillus*, *Penicillium*, *Stemphylium*, and *Alternaria*, among others. The identified anthraquinones revealed diverse biological and pharmacological activities including anticancer, antiviral, antimicrobial, antioxidant, and anti-inflammatory activities. Here, we attempted to highlight their potential as drug candidates *via* exploring their drug-likeness using several molecular descriptors including several drug-likeness rules (Muegge, Ghose, Veber, Egan, and Lipinski). Surprisingly, 133 anthraquinones satisfied all parameters of the 5 tested drug-likeness rules (7, 48, 7, 4, 10, 16, 10, 9, 2, and 20 anthraquinones from *Nigrospora*, *Aspergillus*, *Penicillium*, *Stemphylium*, *Alternaria*, *Trichoderma*, *Eurotium*, *Fusarium*, *Sporendonema casei*, and the other genera, respectively). Noteworthy, all anthraquinones identified from *Trichoderma* species fulfilled the 5 rules. On the other hand, all *Engyodontium album* derived compounds violated the 5 tested rules (Fig. 15, 16, and S†Table 1).

**Table 1:** Anthraquinones and their derivatives isolated from different species of marine-derived fungi with their sources and biological activities. MF = Molecular formula

Compound	MF	Name	Bioactivity	Source	Ref.
1	C_31_H_32_O_12_	Nigrodiquinone A	Displayed no antibacterial or antiviral activity	Zoanthid-derived fungus *Nigrospora* sp.	37
2	C_17_H_22_O_7_	4a-*epi*-9-methoxydihydrodeoxybostrycin	Antibacterial activity	Zoanthid-derived fungus *Nigrospora* sp. and sea anemone-derived fungus *Nigrospora* sp.	37 and 38
3	C_16_H_16_O_7_	10-Deoxybostrycin	Antibacterial and cytotoxic activities	Zoanthid-derived fungus *Nigrospora* sp. and sea anemone-derived fungus *Nigrospora* sp.	37 and 38
4	C_16_H_12_O_6_	3,5,8-Trihydroxy-7-methoxy-2-methyl-anthracene-9,10-dione	Antiviral activity	Zoanthid-derived fungus *Nigrospora* sp. and sea anemone-derived fungus *Nigrospora* sp.	37 and 38
5	C_16_H_12_O_5_	Austrocortirubin	Antiviral and cytotoxic activities	Zoanthid-derived fungus *Nigrospora* sp., mangrove endophytic fungi *Halorosellinia* sp. (no. 1403), and *Guignardia* sp. (no. 4382), sea anemone-derived fungus *Nigrospora* sp., and sea fan-derived fungi *Fusarium* sp. PSU-F14	37–41
6	C_16_H_16_O_6_	Nigrosporin B	Antibacterial, anti-mycobacterial, cytotoxic, and phytotoxic activities	Sea anemone-derived fungus *Nigrospora* sp. and sea fan-derived fungi *Fusarium* sp. PSU-F14	38, 39, 43 and 44
7	C_16_H_20_O_7_	1-Deoxytetrahydrobostrycin	Antibacterial and cytotoxic activities	Sea anemone-derived fungus *Nigrospora* sp., sea fan-derived fungi *Fusarium* sp. PSU-F14 and marine-derived fungus *Aspergillus* sp.	38, 39, 42
8	C_16_H_20_O_8_	Tetrahydrobostrycin	Antibacterial, antimalarial, anti-mycobacterial, and cytotoxic activities	Sea anemone-derived fungus *Nigrospora* sp., sea fan-derived fungi *Fusarium* sp. PSU-F14, and marine-derived fungus *Aspergillus* sp.	38, 39, 42 and 46
9	C_16_H_16_O_7_	4-Deoxybostrycin	Antibacterial, anti-mycobacterial, and cytotoxic activities	Sea anemone-derived fungus *Nigrospora* sp.	38, 43 and 45
10	C_16_H_16_O_8_	Bostrycin	Antibacterial, antimalarial, and cytotoxic activities	Sea anemone-derived fungus *Nigrospora* sp., sea fan-derived fungi *Fusarium* sp. PSU-F14, and marine-derived fungus *Aspergillus* sp.	38, 39, 42 and 45
11	C_25_H_16_O_9_	Aspergiolide A	Cytotoxic activity	Marine-derived fungus *A*. *glaucus*	47 and 48
12	C_26_H_18_O_9_	Aspergiolide B	Cytotoxic activity	Marine-derived fungus *A*. *glaucus*	47
13	C_16_H_12_O_5_	Physcion	Antifungal, antioxidant, and cytotoxic activities	Marine-derived fungi *Microsporum* sp., *A*. *glaucus*, and halotolerant *A*. *variecolor*, and marine algae-derived fungus *A*. *wentii* EN-48	47 and 49–52
14	C_15_H_10_O_5_	Emodin	Antibacterial, antifungal, anti-HCV protease, anti-mycobacterial, and cytotoxic activities	Sea fan-derived fungus *P*. *citrinum* PSU-F51, marine-derived fungi *T*. *aureoviride* PSU-F95, *Trichoderma* sp., *A*. *glaucus*, and halotolerant *A*. *variecolor*, marine lichen-derived fungus *Gliocladium* sp. T31, sea urchin-derived fungus *Monodictys* sp., marine mangrove fungus *Paecilomyces* sp., and marine-derived endophytic fungus *Eu*. *rubrum*	40, 47, 50, 53–61 and 63
15	C_16_H_16_O_5_	Asperflavin	Antioxidant activity	Marine-derived fungus *A*. *glaucus* and marine algae-derived endophytic fungus *Eu. Cristatum* EN-220	47, 62 and 101
16	C_16_H_16_O_5_	Isoasperflavin	Displayed no cytotoxic activity	Marine-derived fungus *A*. *glaucus*	47
17	C_16_H_12_O_5_	Questin	Antioxidant activity	Marine-derived fungi *A*. *glaucus* and halotolerant *A*. *variecolor*, and mangrove-derived fungus *P*. *citrinum* HL-5126	47, 50, 62 and 84
18	C_15_H_10_O_6_	Catenarin	Antibacterial activity	Marine-derived fungi *A*. *glaucus*, *Eu. Rubrum*, and halotolerant *A*. *variecolor*	47, 50, 63 and 103
19	C_16_H_12_O_6_	Rubrocristin	Displayed no antibacterial activity	Marine-derived fungi *A*. *glaucus*, *Eu. Rubrum*, and halotolerant *A*. *variecolor*	47, 50, 63 and 103
20	C_20_H_18_O_10_	(+)-variecolorquinone A	Cytotoxic activity	Marine-derived fungi *A*. *glaucus* and halotolerant *A*. *variecolor*, and marine algae-derived endophytic fungus *Eu. cristatum* EN-220	47, 50 and 101
21	C_32_H_26_O_8_	Physcion-10,10′-bianthrone	Was not evaluated for any relevant bioactivity	Marine-derived fungus *A*. *glaucus*	47
22	C_31_H_24_O_8_	(*trans*)-emodin-physcion bianthrone	Cytotoxic activity	Marine-derived fungus *A*. *glaucus*	47
23	C_31_H_24_O_8_	(*cis*)-emodin-physcion bianthrone	Cytotoxic activity	Marine-derived fungus *A*. *glaucus*	47
24	C_42_H_42_O_13_	6,6′-oxybis(1,3,8-trihydroxy-2-((S)-1-methoxyhexyl)anthracene-9,10-dione)	Antibacterial and cytotoxic activities	Marine-derived fungus *A*. *versicolor*	64
25	C_40_H_38_O_13_	6,6′-oxybis(1,3,8-trihydroxy-2-((S)-1-hydroxyhexyl) anthracene-9,10-dione	Antibacterial activity	Marine-derived fungus *A*. *versicolor*	64
26	C_20_H_20_O_7_	Averantin	Antibacterial, antioxidant, and cytotoxic activities	Marine-derived fungi *A*. *versicolor* and *P*. *purpurogenum* G59	64, 65, 67, 68 and 118
27	C_21_H_22_O_7_	1′-*O*-methyl-averantin	Antibacterial, antioxidant, and cytotoxic activities	Marine-derived fungi *A*. *versicolor* and *P*. *purpurogenum* G59, and the mangrove endophytic fungus (ZSUH-36)	64, 65, 67, 68 and 116
28	C_20_H_18_O_6_	Averythrin	Antioxidant and cytotoxic activities	Marine-derived fungi *A*. *versicolor* and *Aspergillus* sp. SCSIO F063	64, 66 and 67
29	C_22_H_24_O_7_	6,8-*O*,*O*′-dimethyl-averantin	Antibacterial activity	Marine-derived fungus *A*. *versicolor* EN-7	69
30	C_20_H_16_O_7_	Averufin	Antibacterial, antioxidant, antiviral, and cytotoxic activities	Marine-derived fungi *A*. *versicolor* and *A*. *niger* (MF-16), mangrove endophytic fungi ZSUH-36 and (isolate 1850), and mangrove-derived endophytic fungus *A*. *nidulans* MA-143	67, 68, 70, 71, 116 and 119
31	C_21_H_18_O_7_	6-*O*-methyl-averufin	Displayed no antimicrobial activity	Marine-derived fungus *A*. *versicolor* EN-7	69
32	C_22_H_20_O_7_	6,8-*O*,*O*′-dimethyl-averufin	Displayed no anti-neuroinflammatory activity	Marine-derived fungi *Aspergillus* sp. SF-6796 and *A*. *versicolor* EN-7, and the mangrove endophytic fungus (ZSUH-36)	69, 72 and 116
33	C_18_H_12_O_7_	Versicolorin B	Antioxidant activity	Marine-derived fungus *A*. *versicolor* and mangrove endophytic fungus ZSUH-36	67
34	C_18_H_12_O_8_	1′-Hydroxyversicolorin B	Antioxidant and cytotoxic activities	Marine-derived fungus *A*. *versicolor*	67 and 74
35	C_20_H_18_O_7_	Averufanin	Antioxidant activity and inhibitory activity of acyl-CoA: Cholesterol acyltransferase	Marine-derived fungus *A*. *versicolor* and mangrove-derived endophytic fungus *A*. *nidulans* MA-143	67 and 75
36	C_19_H_16_O_7_	Noraverufanin	Anti-HIV activity	Sponge-associated fungi *Microsphaeropsis* sp. and *A*. *versicolor* SCSIO 41016	73 and 112
37	C_20_H_16_O_8_	Nidurufin	Antibacterial, antioxidant, antiviral, and cytotoxic activities	Marine-derived fungi *A*. *versicolor*, *A*. *niger* (MF-16), and *P*. *purpurogenum* G59, and marine-derived mangrove endophytic fungus (isolate 1850)	65, 67, 68, 70 and 119
38	C_22_H_20_O_8_	6,8-*O*,*O*′-dimethyl-nidurufin	Antibacterial activity	Marine-derived fungus *A*. *versicolor* EN-7	69
39	C_18_H_16_O_8_	Versiconol	Cytotoxic activity	Marine-derived fungus *A*. *versicolor*	68
40	C_20_H_20_O_8_	6,8-*O*,*O*′-dimethyl-versiconol	Antibacterial activity	Mangrove endophytic fungus (ZSUH-36) and marine-derived fungus *A*. *versicolor* EN-7	69 and 120
41	C_16_H_12_O_5_	1-Methyl-emodin	Anti-HCV protease and cytotoxic activities	Mangrove endophytic fungi *Halorosellinia* sp. (no. 1403) and *Guignardia* sp. (no. 4382), and red sea endophytic fungus *A*. *versicolor*	40 and 76
42	C_18_H_16_O_6_	Isorhodoptilometrin-1-methyl-ether	Antibacterial and cytotoxic activities	Red sea endophytic fungus *A*. *versicolor*	76
43	C_20_H_16_O_7_	Aversin	Displayed no antimicrobial activity	Mangrove endophytic fungus (ZSUH-36) and marine-derived fungus *A*. *versicolor* EN-7	69 and 120
44	C_20_H_14_O_7_	6,8-*O*,*O*′-dimethyl-versicolorin A	Displayed no antimicrobial activity	Marine-derived fungus *A*. *versicolor* EN-7	69
45	C_16_H_12_O_6_	Evariquinone	Was not evaluated for any relevant bioactivity	Red sea endophytic fungus *A*. *versicolor*	76
46	C_17_H_14_O_6_	7-Hydroxyemodin 6,8-methyl-ether	Was not evaluated for any relevant bioactivity	Red sea endophytic fungus *A*. *versicolor*	76
47	C_15_H_10_O_3_	1-Hydroxy-2-methyl-anthraquinone	Anti-mosquito activity	Marine-derived fungus *A*. *versicolor*	59 and 77
48	C_17_H_14_O_5_	2-(Dimethoxy methyl)-1-hydroxy-9,10-anthraquinone	Antibacterial activity	Marine-derived fungus *A*. *versicolor*	59
49	C_15_H_10_O_2_	Tectoquinone	Was not evaluated for any relevant bioactivity	Marine-derived fungus *A*. *versicolor*	59
50	C_16_H_10_O_5_	Damnacanthal	Antibacterial and anti-mosquito activities	Marine-derived fungus *A*. *versicolor*	59 and 77
51	C_14_H_8_O_4_	Xanthopurpurin	Antibacterial and anti-platelets aggregation activities	Marine-derived fungus *A*. *versicolor*	59 and 78
52	C_15_H_10_O_4_	Rubiadin	Inhibitory activity on formation of advanced glycation end products	Marine-derived fungus *A*. *versicolor*	59 and 79
53	C_15_H_10_O_5_	6-Hydroxyrubiadin	Inhibitory effects on the release of β-hexosaminidase and inhibitory activity on phosphatase of regenerating liver-3	Marine-derived fungus *A*. *versicolor*	59 and 80
54	C_16_H_12_O_5_	Rubianthraquinone	Anti-inflammatory activity	Marine-derived fungus *A*. *versicolor*	59 and 121
55	C_16_H_12_O_7_	Wentiquinone C	Displayed no antioxidant activity	Marine algae-derived fungus *A*. *wentii* EN-48	49
56	C_15_H_10_O_5_	Alatinone	Was not evaluated for any relevant bioactivity	Marine-derived endophytic fungus *A*. *wentii* pt-1	81
57	C_17_H_14_O_5_	5-Hydroxy-1,3-dimethoxy-7-methyl-anthraquinone	Was not evaluated for any relevant bioactivity	Marine-derived endophytic fungus *A*. *wentii* pt-1	81
58	C_16_H_12_O_5_	1,5-Dihydroxy-3-methoxy-7-methyl-anthraquinone	Was not evaluated for any relevant bioactivity	Marine-derived endophytic fungus *A*. *wentii* pt-1	81
59	C_15_H_12_O_6_	Eurotinone	Antioxidant activity and kinase insert domain receptor inhibitory activity	Marine-derived halotolerant fungus *A*. *variecolor*	50 and 122
60	C_16_H_14_O_6_	2-*O*-methyl-eurotinone	Antioxidant activity	Marine mangrove-derived endophytic fungus *Eu. rubrum* and marine-derived halotolerant fungus *A*. *variecolor*	50 and 62
61	C_19_H_16_O_9_	(2*S*)-2,3-dihydroxypropyl 1,6,8-trihydroxy-3-methyl-9,10-dioxo-9,10-dihydro-2-anthracenecarboxylate	Was not evaluated for any relevant bioactivity	Marine-derived halotolerant fungus *A*. *variecolor*	50
62	C_16_H_12_O_6_	Questinol	Anti-inflammatory and anti-obesity activities	Marine-derived halotolerant fungus *A*. *variecolor* and marine-derived fungi *Eu. amstelodami* and *Talaromyces stipitatus* KUFA 0207	50, 82 and 83
63	C_16_H_12_O_6_	Fallacinol	Displayed no significant anti-obesity activity	Marine-derived halotolerant fungus *A*. *variecolor* and marine algae-derived fungus *Talaromyces stipitatus* KUFA 0207	50 and 82
64	C_16_H_12_O_6_	Erythroglaucin	Displayed no antibacterial activity	Marine-derived halotolerant fungus *A*. *variecolor*	50 and 63
65	C_18_H_12_O_7_	Versicolorin C	Antibacterial activity	Marine-derived mangrove endophytic fungus (isolate 1850) and mangrove-derived endophytic fungus *A*. *nidulans* MA-143	71 and 119
66	C_18_H_12_O_7_	Isoversicolorin C	Antibacterial activity	Mangrove-derived endophytic fungus *A*. *nidulans* MA-143	71
67	C_20_H_18_O_7_	Norsolorinic acid	Was not evaluated for any relevant bioactivity	Mangrove-derived endophytic fungus *A*. *nidulans* MA-143	71
68	C_21_H_22_O_7_	(1′*S*) 6-*O*-methyl-averantin	Displayed no cytotoxic activity	Marine-derived fungus *Aspergillus* sp. SCSIO F063	66
69	C_22_H_24_O_7_	(1′*S*) 6,1′-*O*,*O*′-dimethyl-averantin	Displayed no cytotoxic activity	Marine-derived fungus *Aspergillus* sp. SCSIO F063	66
70	C_24_H_28_O_7_	Averantin-1′-butyl ether	Cytotoxic activity	Marine-derived fungus *Aspergillus* sp. SCSIO F063	66
71	C_20_H_19_ClO_7_	(1′*S*)-7-chloroaverantin	Cytotoxic activity	Marine-derived fungus *Aspergillus* sp. SCSIO F063	66
72	C_21_H_21_ClO_7_	(1′*S*) 6-*O*-methyl-7-chloroaverantin	Cytotoxic activity	Marine-derived fungus *Aspergillus* sp. SCSIO F063	66
73	C_21_H_21_ClO_7_	(1′*S*) 1′-*O*-methyl-7-chloroaverantin	Cytotoxic activity	Marine-derived fungus *Aspergillus* sp. SCSIO F063	66
74	C_22_H_23_ClO_7_	(1′*S*) 6,1′-*O*,*O*′-dimethyl-7-chloroaverantin	Displayed no cytotoxic activity	Marine-derived fungus *Aspergillus* sp. SCSIO F063	66
75	C_24_H_27_ClO_7_	(1′*S*) 7-chloroaverantin-1′-butyl ether	Cytotoxic activity	Marine-derived fungus *Aspergillus* sp. SCSIO F063	66
76	C_20_H_17_ClO_6_	7-Chloroaverythrin	Displayed no cytotoxic activity	Marine-derived fungus *Aspergillus* sp. SCSIO F063	66
77	C_21_H_19_ClO_6_	6-*O*-methyl-7-chloroaverythrin	Cytotoxic activity	Marine-derived fungus *Aspergillus* sp. SCSIO F063	66
78	C_21_H_21_ClO_6_	(1′*S*) 6-*O*-methyl-7-bromoaverantin	Cytotoxic activity	Marine-derived fungus *Aspergillus* sp. SCSIO F063	66
79	C_22_H_23_BrO_7_	(1′*S*) 6,1′-*O*,*O*′-dimethyl-7-bromoaverantin	Displayed no cytotoxic activity	Marine-derived fungus *Aspergillus* sp. SCSIO F063	66
80	C_23_H_26_O_7_	6,8,1′-*O*,*O*′,*O*′′-trimethyl-averantin	Anti-inflammatory activity	Marine-derived fungus *Aspergillus* sp. SF-6796 and mangrove endophytic fungus ZSUH-36	72 and 116
81	C_28_H_24_O_10_	Penicillanthranin A	Antibacterial and cytotoxic activities	Sea fan-derived fungus *P*. *citrinum* PSU-F51	53
82	C_28_H_24_O_11_	Penicillanthranin B	Displayed no cytotoxic activity	Sea fan-derived fungus *P*. *citrinum* PSU-F51	53
83	C_15_H_10_O_4_	Chrysophanol	Anti-acetylcholinesterase, antibacterial, and cytotoxic activities	Sea fan-derived fungus *P*. *citrinum* PSU-F51, marine-derived fungi *T*. *aureoviride* PSU-F95 and *Trichoderma* sp., mangrove endophytic fungi *Halorosellinia* sp. (no. 1403) and *Guignardia* sp. (no. 4382), sea urchin-derived fungus *Monodictys* sp., and marine mangrove fungus *Paecilomyces* sp.	40, 53–55, 57 and 100
84	C_15_H_10_O_6_	ω-hydroxyemodin	Antibacterial, anti-mycobacterial, anti-obesity, and cytotoxic activities	Sea fan-derived fungus *P*. *citrinum* PSU-F51, mangrove-derived fungus *P*. *citrinum* HL-5126, marine-derived fungi *T*. *aureoviride* PSU-F95, and *Talaromyces stipitatus* KUFA 0207, and marine lichen-derived fungus *Gliocladium* sp. T31	53, 54, 56, 61, 82 and 84
85	C_18_H_13_ClO_7_	2′-Acetoxy-7-chlorocitreorosein	Antibacterial activity	Mangrove-derived fungus *P*. *citrinum* HL-5126	84
86	C_15_H_10_O_9_S	Citreorosein-3-*O*-sulphate	Was not evaluated for any relevant bioactivity	Marine-derived fungus *P*. *oxalicum* 2HL-*M*-6	85
87	C_15_H_10_O_8_S	Emodin-3-*O*-sulphate	Was not evaluated for any relevant bioactivity	Marine-derived fungus *P*. *oxalicum* 2HL-*M*-6	85
88	C_15_H_10_O_5_	Aloe-emodin	Antibacterial and antimalarial activities	Marine-derived fungus *P*. *oxalicum* 2HL-*M*-6	85–87
89	C_21_H_19_NO_8_	Emodacidamide A	Immunomodulatory activity	Marine-derived fungus *Penicillium* sp. SCSIO sof101	88
90	C_20_H_17_NO_8_	Emodacidamide B	Immunomodulatory activity	Marine-derived fungus *Penicillium* sp. SCSIO sof101	88
91	C_20_H_16_ClNO_8_	Emodacidamide C	Immunomodulatory activity	Marine-derived fungus *Penicillium* sp. SCSIO sof101	88
92	C_22_H_21_NO_8_	Emodacidamide D	Immunomodulatory activity	Marine-derived fungus *Penicillium* sp. SCSIO sof101	88
93	C_21_H_19_NO_8_	Emodacidamide E	Immunomodulatory activity	Marine-derived fungus *Penicillium* sp. SCSIO sof101	88
94	C_21_H_18_ClNO_8_	Emodacidamide F	Immunomodulatory activity	Marine-derived fungus *Penicillium* sp. SCSIO sof101	88
95	C_21_H_18_ClNO_8_	Emodacidamide G	Immunomodulatory activity	Marine-derived fungus *Penicillium* sp. SCSIO sof101	88
96	C_18_H_13_NO_8_	Emodacidamide H	Immunomodulatory activity	Marine-derived fungus *Penicillium* sp. SCSIO sof101	88
97	C_15_H_8_O_7_	Emodic acid	Inhibitory activity on tyrosine kinase proteins	Marine-derived endophytic fungus *Eu. rubrum* and marine-derived fungus *Penicillium* sp. SCSIO sof101	58, 88 and 89
98	C_15_H_9_ClO_6_	2-Chloro-1,3,8 trihydroxy-6 (hydroxymethyl)-anthracene-9,10 dione	Immunomodulatory activity	Marine-derived fungus *Penicillium* sp. SCSIO sof101	88
99	C_16_H_16_O_8_	Altersolanol A	Antibacterial and cytotoxic activities, as well as protein kinase inhibitory activity	Mangrove-derived fungus *Stemphylium* sp. 33 231 and coral-associated fungus *Stemphylium lycopersici*	90, 92 and 94
100	C_16_H_18_O_7_	Dihydroaltersolanol A	Displayed no antibacterial or cytotoxic activity	Mangrove-derived fungus *Stemphylium* sp. 33 231, deep-sea-derived fungus *Alternaria tenuissima* DFFSCS013, and soft coral-derived *Alternaria* sp. ZJ-2008003	90, 93, 99
101	C_16_H_16_O_6_	Altersolanol B	Antibacterial, anticoagulant, anti-mycobacterial, and cytotoxic activities	Mangrove-derived fungus *Stemphylium* sp. 33 231, mangrove endophytic fungus *Alternaria* sp. ZJ9-6B, coral-associated fungus *Stemphylium lycopersici* and *Alternaria* sp. ZJ-2008003, deep sea-derived fungus *Alternaria tenuissima* DFFSCS013 and marine-derived fungus *Sporendonema casei* HDN16-802	90, 92, 93, 98, 99 and 107
102	C_16_H_20_O_6_	Tetrahydroaltersolanol B	Antibacterial and antifungal activities	Mangrove-derived fungi *Phomopsis* sp.PSU-MA214, *Stemphylium* sp. 33 231, and *Phoma* sp. L28, mangrove endophytic fungus *Alternaria* sp. ZJ9-6B, and soft coral-derived *Alternaria* sp. ZJ-2008003	90, 91, 93, 98 and 111
103	C_18_H_18_O_7_	2-*O*-acetylaltersolanol B	Antibacterial activity	Mangrove-derived fungus *Stemphylium* sp. 33 231	90
104	C_16_H_16_O_7_	Altersolanol C	Antibacterial activity	Mangrove-derived fungus *Stemphylium* sp. 33 231 and soft coral-derived *Alternaria* sp. ZJ-2008003	90 and 93
105	C_16_H_20_O_7_	Altersolanol L	Antifungal and cytotoxic activities	Mangrove-derived fungi *Stemphylium* sp. 33 231 and *Phoma* sp. L28, and deep-sea derived fungus *Alternaria tenuissima* DFFSCS013 and soft coral-derived *Alternaria* sp. ZJ-2008003	90, 91, 93, 99 and 123
106	C_18_H_22_O_8_	2-*O*-acetylaltersolanol L	Displayed no antibacterial or cytotoxic activity	Mangrove-derived fungus *Stemphylium* sp. 33 231	90
107	C_16_H_20_O_8_	Ampelanol	Cytotoxic activity	Mangrove-derived fungi *Phomopsis* sp.PSU-MA214, *Stemphylium* sp. 33 231, and *Phoma* sp. L28, coral-associated fungus *Stemphylium lycopersici* and *Alternaria* sp. ZJ-2008003 and, deep-sea derived fungus *Alternaria tenuissima* DFFSCS013	90–94, 99 and 111
108	C_16_H_12_O_5_	Macrosporin	Antibacterial, antifungal, and cytotoxic activities as well as protein kinases' inhibitory activity	Mangrove-derived fungi *Phomopsis* sp.PSU-MA214, *Stemphylium* sp. 33 231, *Alternaria* sp. ZJ9-6B and *Phoma* sp. L28 and coral-associated fungus *Stemphylium lycopersici* and *Alternaria* sp. ZJ-2008003	90–94, 98, 111 and 123
109	C_16_H_12_O_8_S	Macrosporin-7-*O*-sulphate	Cytotoxic activity	Mangrove-derived fungus *Stemphylium* sp. 33 231	90 and 94
110	C_24_H_24_O_11_	Macrosporin 2-*O*-(6′-acetyl)-α-d-glucopyranoside	Brine shrimp lethality	Mangrove-derived fungus *Stemphylium* sp. 33 231	90
111	C_16_H_16_O_9_	Auxarthrol C	Antibacterial activity	Mangrove-derived fungus *Stemphylium* sp. 33 231 and coral-associated fungus *Stemphylium lycopersici*	90 and 92
112	C_32_H_26_O_13_	Alterporriol A	Displayed no antibacterial or cytotoxic activity	Mangrove-derived fungus *Stemphylium* sp. 33 231	90
113	C_32_H_26_O_13_	Alterporriol B	Antibacterial activity	Mangrove-derived fungus *Stemphylium* sp. 33 231	90
114	C_32_H_22_O_10_	Alterporriol C	Antibacterial and antiviral activities	Mangrove-derived fungus *Stemphylium* sp. 33 231 and soft coral-derived *Alternaria* sp. ZJ-2008003	90 and 93
115	C_32_H_30_O_16_	Alterporriol D	Antibacterial and cytotoxic activities	Mangrove-derived fungus *Stemphylium* sp. 33 231	90 and 94
116	C_32_H_22_O_10_	Alterporriol E	Antibacterial and cytotoxic activities	Mangrove-derived fungus *Stemphylium* sp. 33 231	90 and 94
117	C_32_H_26_O_13_	Alterporriol N	Antibacterial and anti-inflammatory activities	Marine-derived fungus *Stemphylium* sp. FJJ006 and mangrove-derived fungus *Stemphylium* sp. 33 231	90, 95 and 97
118	C_32_H_22_O_10_	Alterporriol Q	Antiviral activity	Mangrove-derived fungus *Stemphylium* sp. 33 231	90 and 93
119	C_32_H_22_O_10_	Alterporriol R	Displayed no antiviral, antibacterial or cytotoxic activity	Mangrove-derived fungus *Stemphylium* sp. 33 231	90 and 93
120	C_32_H_30_O_13_	Alterporriol T	Displayed no antibacterial activity	Mangrove-derived fungus *Stemphylium* sp. 33 231	90
121	C_32_H_30_O_12_	Alterporriol U	Antibacterial activity	Mangrove-derived fungus *Stemphylium* sp. 33 231	90
122	C_32_H_22_O_10_	Alterporriol V	Antibacterial activity	Mangrove-derived fungus *Stemphylium* sp. 33 231	90
123	C_32_H_26_O_12_	Alterporriol W	Displayed no antibacterial or cytotoxic activity	Mangrove-derived fungus *Stemphylium* sp. 33 231	90
124	C_32_H_26_O_12_	Alterporriol F	Anti-inflammatory and cytotoxic activities	Marine-derived fungus *Stemphylium* sp. FJJ006	95 and 96
125	C_32_H_26_O_13_	Alterporriol G	Antibacterial, anti-inflammatory, and cytotoxic activities as well as protein kinase inhibitory activity	Marine-derived fungus *Stemphylium* sp. FJJ006	95, 97 and 123
126	C_32_H_26_O_13_	Alterporriol Z_1_	Anti-inflammatory activity	Marine-derived fungus *Stemphylium* sp. FJJ006	95
127	C_32_H_26_O_13_	Alterporriol Z_2_	Anti-inflammatory activity	Marine-derived fungus *Stemphylium* sp. FJJ006	95
128	C_33_H_28_O_13_	Alterporriol Z_3_	Displayed no antibacterial or cytotoxic activity	Marine-derived fungus *Stemphylium* sp. FJJ006	95
129	C_22_H_22_O_10_	Macrosporin 2-*O*-α-d-glucopyranoside	Displayed no cytotoxic activity	Coral associated fungus *Stemphylium lycopersici*	92
130	C_32_H_30_O_12_	Alterporriol Y	Displayed no cytotoxic activity	Coral associated fungus *Stemphylium lycopersici*	92
131	C_32_H_26_O_11_	Alterporriol K	Cytotoxic activity	Mangrove endophytic fungus *Alternaria* sp. ZJ9-6B	98
132	C_32_H_26_O_12_	Alterporriol L	Cytotoxic activity	Mangrove endophytic fungus *Alternaria* sp. ZJ9-6B	98
133	C_32_H_26_O_12_	Alterporriol M	Was not evaluated for any relevant bioactivity	Mangrove endophytic fungus *Alternaria* sp. ZJ9-6B	98
134	C_32_H_30_O_14_	Alterporriol O	Displayed no antibacterial, antiviral, or cytotoxic activity	Soft coral derived *Alternaria* sp. ZJ-2008003	93
135	C_32_H_26_O_12_	Alterporriol P	Cytotoxic activity	Soft coral derived *Alternaria* sp. ZJ-2008003	93
136	C_16_H_20_O_6_	Tetrahydroaltersolanol C	Antiviral activity	Mangrove-derived fungus *Phomopsis* sp.PSU-MA214 and soft coral-derived fungus *Alternaria* sp. ZJ-2008003	93 and 111
137	C_16_H_20_O_6_	Tetrahydroaltersolanol D	Displayed no antibacterial, antiviral, or cytotoxic activity	Soft coral derived *Alternaria* sp. ZJ-2008003	93
138	C_16_H_20_O_6_	Tetrahydroaltersolanol E	Displayed no antibacterial, antiviral, or cytotoxic activity	Soft coral derived *Alternaria* sp. ZJ-2008003	93
139	C_18_H_22_O_7_	Tetrahydroaltersolanol F	Displayed no antibacterial, antiviral, or cytotoxic activity	Soft coral derived *Alternaria* sp. ZJ-2008003	93
140	C_25_H_28_O_10_	Anthrininone A	Inhibitory activity on protein tyrosine phosphatases and stimulatory effect on intracellular calcium levels	Deep-sea derived fungus *Alternaria tenuissima* DFFSCS013	99
141	C_16_H_12_O_6_	6-*O*-methyl-alaternin	Inhibitory activity on protein tyrosine phosphatases	Deep-sea derived fungus *Alternaria tenuissima* DFFSCS013	99
142	C_16_H_16_O_5_	(3*R*)-1-deoxyaustrocortilutein	Displayed no stimulation of intracellular calcium level	Deep-sea derived fungus *Alternaria tenuissima* DFFSCS013	99
143	C_15_H_16_O_5_	Harzianumnone A	Displayed no anti-acetylcholinesterase or DNA Topo I inhibitory activities	Coral-derived fungus *T*. *harzianum* XS-20090075	100
144	C_15_H_16_O_5_	Harzianumnone B	Displayed no anti-acetylcholinesterase or DNA Topo I inhibitory activities	Coral-derived fungus *T*. *harzianum* XS-20090075	100
145	C_15_H_10_O_4_	Phomarin	Anti-acetylcholinesterase activity	Coral-derived fungus *T*. *harzianum* XS-20090075	100
146	C_15_H_10_O_5_	ω-hydroxydigitoemodin	Anti-acetylcholinesterase activity	Coral-derived fungus *T*. *harzianum* XS-20090075	100
147	C_15_H_10_O_3_	Pachybasin	Anti-acetylcholinesterase and cytotoxic activities	Marine-derived fungus *T*. *aureoviride* PSU-F95 and mangrove endophytic fungi *Halorosellinia* sp. (no. 1403) and *Guignardia* sp. (no. 4382), and sea urchin-derived fungus *Monodictys* sp	40, 41, 54, 55 and 100
148	C_17_H_14_O_6_	(+)-2′*S*-isorhodoptilometrin	Anti-acetylcholinesterase, antibacterial, and cytotoxic activities, as well as DNA Topo I inhibitory activity	Coral-derived fungus *T*. *harzianum* XS-20090075, marine lichen-derived fungus *Gliocladium* sp. T31, and marine-derived fungus *T*. *aureoviride* PSU-F95	54, 56 and 100
149	C_15_H_10_O_4_	ω-hydroxypachybasin	Antibacterial, and cytotoxic activities, as well as DNA Topo I inhibitory activity	Marine-derived fungus *T*. *aureoviride* PSU-F95 and coral-derived fungus *T*. *harzianum* XS-20090075	54 and 100
150	C_15_H_14_O_5_	Coniothranthraquinone 1	Antibacterial activity	Marine-derived fungus *T*. *aureoviride* PSU-F95	54
151	C_15_H_14_O_6_	Trichodermaquinone	Antibacterial activity	Marine-derived fungus *T*. *aureoviride* PSU-F95	54
152	C_15_H_10_O_4_	2-Methyl-quinizarin	Was not evaluated for any relevant bioactivity	Marine-derived fungus *T*. *aureoviride* PSU-F95	54
153	C_15_H_10_O_4_	1-Hydroxy-3-methoxyanthraquinone	Was not evaluated for any relevant bioactivity	Marine-derived fungus *T*. *aureoviride* PSU-F95	54
154	C_15_H_16_O_5_	Coniothyrinone A	Antibacterial and antiangiogenetic activities	Marine-derived fungus *Trichoderma* sp. and saline-alkali plant endophytic fungus *Eu. rubrum*	60 and 103
155	C_15_H_14_O_6_	Lentisone	Antibacterial and antiangiogenetic activities	Marine-derived fungus *Trichoderma* sp	60
156	C_15_H_12_O_5_	9-Dehydroxyeurotinone	Antibacterial and cytotoxic activities	Marine-derived endophytic fungus *Eu. rubrum*	58
157	C_16_H_14_O_5_	2-*O*-Methyl-9-dehydroxyeurotinone	Antioxidant activity	Marine-derived endophytic fungus *Eu. rubrum* and marine mangrove-derived endophytic fungus *Eu. rubrum*	58 and 62
158	C_21_H_22_O_9_	2-*O*-Methyl-4-*O*-(α-d-ribofuranosyl)-9-dehydroxyeurotinone	Antioxidant activity	Marine mangrove-derived endophytic fungus *Eu. rubrum*	62
159	C_21_H_20_O_9_	3-*O*-(α-d-ribofuranosyl)-questin	Antibacterial and antioxidant activities	Marine mangrove-derived endophytic fungus *Eu. rubrum* and marine algae-derived endophytic fungus *Eu. cristatum* EN-220	62 and 101
160	C_33_H_32_O_10_	Eurorubrin	Antibacterial and antioxidant activities as well as brine shrimp lethality	Marine mangrove-derived endophytic fungus *Eu. rubrum* and marine algae-derived endophytic fungus *Eu. cristatum* EN-220	62 and 101
161	C_21_H_20_O_10_	3-*O*-(α-d-ribofuranosyl)questinol	Displayed no antibacterial activity or brine shrimp lethality	Marine algae-derived endophytic fungus *Eu. cristatum* EN-220	101
162	C_21_H_24_O_9_	Asperflavin ribofuranoside	Antibacterial and antioxidant activities	Marine algae-derived endophytic fungus *Eu. cristatum* EN-220 and marine-derived algicolous fungus *Microsporum* sp	101 and 102
163	C_15_H_14_O_5_	Rubrumol	Relaxation activity on Topo I enzyme	Saline-alkali plant endophytic fungus *Eu. rubrum*	103
164	C_16_H_16_O_6_	Nigrosporin A	Antibacterial, cytotoxic, and phytotoxic activities	Sea fan-derived fungus *Fusarium* sp. PSU-F14	39 and 44
165	C_16_H_20_O_7_	Fusaranthraquinone	Displayed no antibacterial activity	Sea fan-derived fungus *Fusarium* sp. PSU-F14	39
166	C_16_H_18_O_6_	Fusaquinon A	Cytotoxic activity	Marine-derived fungus *Fusarium* sp. ZH-210	104 and 105
167	C_16_H_20_O_8_	Fusaquinon B	Cytotoxic activity	Marine-derived fungus *Fusarium* sp. ZH-210	104
168	C_16_H_20_O_7_	Fusaquinon C	Cytotoxic activity	Marine-derived fungus *Fusarium* sp. ZH-210	104
169	C_21_H_20_O_5_	6,8-Dimethoxy-1-methyl-2-(3-oxobutyl)anthracene-9,10-dione	Cytotoxic activity	Mangrove endophytic fungus *Fusarium* sp. ZZF60	106
170	C_17_H_12_O_7_	5-Acetyl-2-methoxy-1,4,6-trihydroxy-anthraquinone	Was not evaluated for any relevant bioactivity	Marine endophytic fungus *Fusarium* sp. b77	124
171	C_33_H_28_O_12_	Engyodontochone A	Antibacterial, antifungal, and cytotoxic activities	Marine-derived fungus *Engyodontium album* strain LF069	23
172	C_33_H_28_O_12_	JBIR-99	Antibacterial, antifungal, and cytotoxic activities	Marine-derived fungus *Engyodontium album* strain LF069	23
173	C_33_H_28_O_12_	Engyodontochone B	Antibacterial, antifungal, and cytotoxic activities	Marine-derived fungus *Engyodontium album* strain LF069	23
174	C_33_H_28_O_12_	Engyodontochone C	Antibacterial and cytotoxic activities	Marine-derived fungus *Engyodontium album* strain LF069	23
175	C_33_H_30_O_13_	Engyodontochone F	Antibacterial and antifungal activities	Marine-derived fungus *Engyodontium album* strain LF069	23
176	C_33_H_30_O_13_	Engyodontochone E	Antibacterial activity	Marine-derived fungus *Engyodontium album* strain LF069	23
177	C_33_H_28_O_12_	Engyodontochone D	Was not evaluated for any relevant bioactivity	Marine-derived fungus *Engyodontium album* strain LF069	23
178	C_16_H_16_O_7_	4-Dehydroxyaltersolanol A	Antibacterial and anticoagulant activities	Marine-derived fungus *Sporendonema casei* HDN16-802	107
179	C_16_H_18_O_8_	Auxarthrol D	Antibacterial, anticoagulant, and cytotoxic activities	Marine-derived fungus *Sporendonema casei* HDN16-802	107
180	C_16_H_20_O_9_	Auxarthrol E	Anticoagulant activity	Marine-derived fungus *Sporendonema casei* HDN16-802	107
181	C_16_H_20_O_8_	Auxarthrol F	Antibacterial, anticoagulant, and cytotoxic activities	Marine-derived fungus *Sporendonema casei* HDN16-802	107
182	C_16_H_17_ClO_8_	Auxarthrol G	Antibacterial, anticoagulant, and antifungal activities	Marine-derived fungus *Sporendonema casei* HDN16-802	107
183	C_16_H_18_O_8_	Auxarthrol H	Anticoagulant activity	Marine-derived fungus *Sporendonema casei* HDN16-802	107
184	C_17_H_14_O_4_	1,3-Dimethoxy-6-methyl-anthracene-9,10-dione	Cytotoxic activity	Mangrove endophytic fungi *Halorosellinia* sp. No. 1403 and *Guignardia* sp. No. 4382	40
185	C_15_H_10_O_4_	Demethoxyaustrocortirubin	Cytotoxic activity	Mangrove endophytic fungi *Halorosellinia* sp.No. 1403 and *Guignardia* sp. No. 4382	40 and 41
186	C_14_H_8_O_4_	Dantron	Cytotoxic activity	Mangrove endophytic fungi *Halorosellinia* sp.No. 1403 and *Guignardia* sp. No. 4382	40
187	C_16_H_12_O_5_	1,3-Dihydroxy-6-methoxy-8-methyl-anthracene-9,10-dione	Cytotoxic activity	Mangrove endophytic fungi *Halorosellinia* sp.No. 1403 and *Guignardia* sp. No. 4382	40
188	C_17_H_14_O_5_	1-Hydroxy-2,4-dimethoxy-7-methyl-anthracene-9,10-dione	Cytotoxic activity	Mangrove endophytic fungi *Halorosellinia* sp.No. 1403 and *Guignardia* sp. No. 4382	40
189	C_16_H_12_O_4_	8-Hydroxy-1-methoxy-3-methyl-9,10-anthraquinone	Cytotoxic activity	Mangrove endophytic fungi *Halorosellinia* sp.No. 1403 and *Guignardia* sp. No. 4382	40
190	C_17_H_14_O_6_	1,7-Dihydroxy-2,4-dimethoxy-6-methyl-anthracene-9,10-dione	Cytotoxic activity	Mangrove endophytic fungi *Halorosellinia* sp.No. 1403 and *Guignardia* sp. No. 4382	40
191	C_14_H_8_O_5_	1,3,8-Trihydroxyanthraquinone	Cytotoxic activity	Mangrove endophytic fungi *Halorosellinia* sp.No. 1403 and *Guignardia* sp. No. 4382	40
192	C_16_H_12_O_6_	1,4,7-Trihydroxy-2-methoxy-6-methyl-9,10-anthraquinone	Displayed no cytotoxic activity	Mangrove endophytic fungi *Halorosellinia* sp.No. 1403 and *Guignardia* sp. No. 4382	40
193	C_16_H_16_O_8_	SZ-685C	Cytotoxic activity	Mangrove endophytic fungus *Halorosellinia* sp. No. 1403	108–110
194	C_18_H_20_O_6_	Phomopsanthraquinone	Antibacterial and cytotoxic activities	Mangrove-derived fungus *Phomopsis* sp. PSU-MA214	111
195	C_16_H_12_O_4_	1-Hydroxy-3-methoxy-6-methyl-anthraquinone	Displayed no antibacterial or cytotoxic activity	Mangrove-derived fungus *Phomopsis* sp. PSU-MA214	111
196	C_16_H_12_O_7_	Tetrahydroxyanthraquinone	Protein kinases' inhibitory activity	Sponge-associated fungus *Microsphaeropsis* sp	112
197	C_17_H_14_O_7_	Methoxyl-tetrahydroxyanthraquinone	Protein kinases' inhibitory activity	Sponge-associated fungus *Microsphaeropsis* sp	112
198	C_17_H_14_O_8_	1,2,3,6,8-Pentahydroxy-7-[(1R)-1-methoxyethyl]-9,10-anthraquinone	Protein kinases' inhibitory activity	Sponge-associated fungus *Microsphaeropsis* sp	112
199	C_15_H_10_O_6_	Lunatin	Antibacterial activity	Sponge-derived fungus *Curvularia lunata*	113
200	C_30_H_22_O_12_	Cytoskyrin A	Antibacterial activity	Sponge-derived fungus *Curvularia lunata*	113
201	C_14_H_8_O_6_	Rheoemodin	Displayed no significant anti-obesity activity	Marine sponge-associated fungus *Talaromyces stipitatus* KUFA 0207	82
202	C_30_H_18_O_10_	2, 2′-*Bis*-(7-methyl-1,4,5-trihydroxy-anthracene-9,10-dione)	Was not evaluated for any relevant activity	Marine sponge-associated fungus *Talaromyces stipitatus* KUFA 0207	82
203	C_17_H_14_O_5_	7-Methoxymacrosporin	Antifungal activity	Mangrove-derived fungus *Phoma* sp. L28	91
204	C_21_H_20_O_5_	7-(γ,γ)-Dimethyl-allyloxy-macrosporin	Antifungal activity	Mangrove-derived fungus *Phoma* sp. L28	91
205	C_16_H_12_O_5_	Monodictyquinone A	Antibacterial and antifungal activities	Sea urchin-derived fungus *Monodictys* sp	55
206	C_16_H_12_O_6_	1,3,6-Trihydroxy-7-(1-hydroxyethyl) anthracene-9,10-dione	Inhibitory activity against α-glycosidase	Mangrove-derived fungus *Cladosporium* sp. HNWSW-1	114
207	C_17_H_12_O_7_	Phaseolorin I	Displayed no cytotoxic activity	Deep-sea sediment-derived fungus *Diaporthe phaseolorum* FS431	115
208	C_22_H_22_O_7_	6,8-*O*,*O*′-Dimethyl-averufanin	Antifungal and phytotoxic activities, as well as brine shrimp lethality	Mangrove endophytic fungus ZSUH-36	116 and 117

Topological polar surface area (TPSA), another measure, is the sum of the surfaces of all the polar atoms present in a molecule. TPSA has a substantial effect on the potential of a compound to penetrate through the cell membranes and blood–brain barrier. Veber highlighted those compounds with TPSA ≤ 140 A^2^ tend to be well absorbed and able to reach their molecular target within the body cells. Egan stated that molecules with TPSA less than 132 A^2^ and log-P between −1 and 6 could be considered leads with high drug-likeness potential and good orally bioavailability. Muegge utilized a pharmacophore point filter based on very simple structural rules to differentiate between drug-like and nondrug-like molecules, among them TPSA not greater than 150 A^2^ as well as rotatable bonds (RB), not more than 15. All anthraquinones from *Fusarium*, *Trichoderma*, *Nigrospora* (except compound 1), *Aspergillus* (except compounds 11, 12, 20, 24, 25, 39, and 61), *Penicillium* (except compounds 81, 82, 86, and 89–96), *Stemphylium* (except compounds 110–130), *Alternaria* (except compounds 114, 131–135, and 140), *Eurotium* (except compounds 20, 160, and 161), *Sporendonema casei* (except compound 180), and the other genera (except compounds 200 and 202) had TPSA less than 150 A^2^. On the other hand, all *Engyodontium album* derived compounds had TPSA greater than 150 A^2^. All anthraquinones had RB less than 15 (Fig. 15 and ESI Table S1†).

Oral bioavailability, bioavailability score (BS), is another descriptor that indicates the possibility of a compound to be bioavailable with more than 10% in the absorption assays. Molecules obeying the Lipinski rule with BS of 0.55 are considered orally bioavailable. Interestingly, 166 anthraquinones showed a BS of 0.55. In alignment with other parameters, all *Fusarium* and *Trichoderma* derived anthraquinones showed a BS of 0.55 and all *Engyodontium album* derived compounds had a BS of 0.11. Compounds 3, 6, and 9, are of special interest as they showed a good BS of 0.56. Some other compounds, among them 10, 87, 97, and 109, showed good BS (0.56); however, violated one or more drug-likeness rules (Fig. 15 and ESI Table S1†).

Oral bioavailability relies as well on the degree of the molecular flexibility of the molecule. Candidates with an extreme degree of flexibility do not typically display acceptable bioavailability as they tend to be less planar and with very complex 3D shapes. The sp^3^ carbons fraction (Fraction Csp^3^) and the number of RB are two crucial measures for molecular flexibility. Csp^3^ is the ratio of the sp^3^ carbon atoms to the total carbons present in a given compound. It assigns the degree of carbon saturation, characterizes the space complexity, and correlates to the solubility of the compound. A Csp3 score between 0.25 and 1 is considered optimum for drug-likeness. One hundred anthraquinones, distributed in all marine fungi species, displayed a Csp^3^ score ranging between 0.28 and 0.6. The water solubility, expressed as log S, is another essential measure for drug bioavailability. Compounds with poor water solubility have poor absorption and oral bioavailability, as well as low formulation potential. Anthraquinones revealed different solubility orders as *Sporendonema casei* derived compounds were the most soluble (mean value of −1.46), followed by *Nigrospora* sp (mean value of −2.34), while *Engyodontium album* derived anthraquinones, as expected, were the most poorly soluble (mean value of −5.44) (Fig. 15 and ESI Table S1†).

Gastrointestinal (GI) absorption, blood–brain barrier (BBB) permeation, P-glycoprotein (P-gp) substrate and cytochrome P450 members inhibition potentials were also surveyed to draw insight about the pharmacokinetic behavior of the reviewed anthraquinones. Twenty anthraquinones (47, 48, 49, 51, 52, 57, 83, 147, 152, 153, 157, 169, 184, 185, 186, 188, 189, 195, 203, and 204) showed high GI absorption, passively crossed BBB and did not show any potential for P-gp substrate (ESI Table S2†). Surprisingly, compound 169 obeyed all the surveyed parameters (5 drug-likeness rules, log *P*, Csp^3^, RB, TPSA, log *S*, GI, BBB, Pgp) and the other 19 anthraquinones as well except for fraction Csp^3^. Noteworthy, all but one of the 20 anthraquinones have two benzenoid aromatic rings and two C=O groups. Also, several anthraquinones showed potential inhibition for some CYP 450 isoforms which necessitates awareness when co-administered with possible substrates of these enzymes (ESI Table S2†).

To sum up, marine fungi are a promising source of biologically active anthraquinones that obeyed all the criteria of several drug-likeness rules with promising pharmacokinetic behavior which promotes their utilization as well as further research to isolate their individual components and determine their pharmacological effects.

## Conclusions and future prospective

**Fig. 17:**
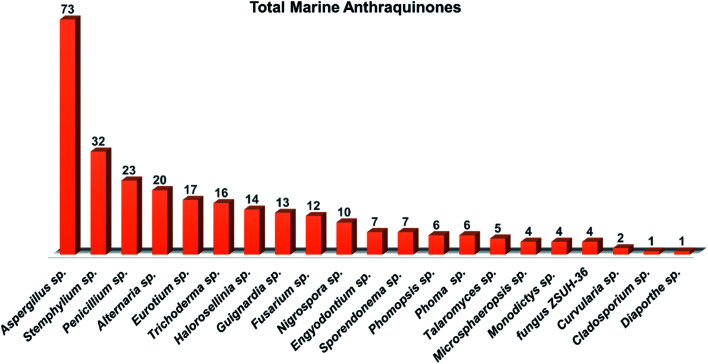
Distribution and total anthraquinones and their derivatives isolated from different species of marine-derived fungi.

**Fig. 18:**
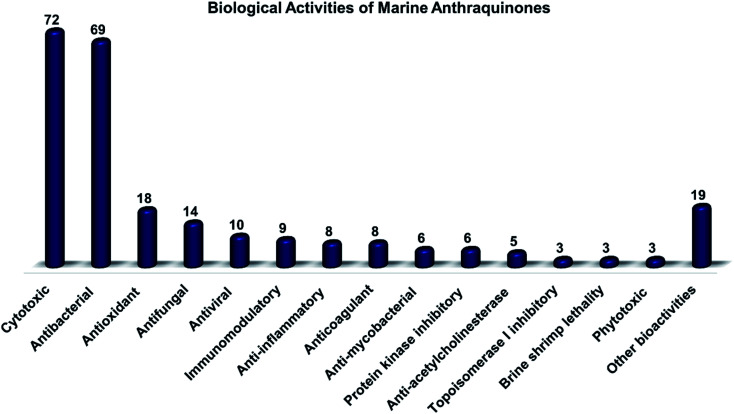
Total biological activities of various anthraquinones and their derivatives isolated from different species of marine-derived fungi.

The marine phoma is representing the most, the greatest and most diverse ecological structure on the planet. Over seven decades, marine natural products (MNPs) have owned credits and been privileged as a robust and sustainable supplier for pharmacologically active compounds that meet a huge interest in pharmaceutical and economical applications. Marine-derived fungi are valuable sources of structurally diverse MNPs due to their various habitats that range from the warm to the colder areas, and even at extreme temperatures and pressure like in hydrothermal outlets. One of the fascinating classes of fungal derived natural products is the anthraquinones. Herein, we presented a comprehensive literature review centered on marine-derived anthraquinones as a unique group of fungal polyketides over the period 2000–2020 from twenty marine fungal genera. A list of 208 anthraquinones have been reported from different marine fungi, featuring a myriad of structural and biological diversities. Investigating such extensive chemo-biological data has implied two remarkable points. First, it was clear that the marine fungi of the three genera *Aspergillus* sp., *Stemphylium* sp., and *Penicillium* sp., are the most creative fungal genera in terms of producing of anthraquinones. Secondly, the most common reported bioactivity was cytotoxicity, where a notable number of seventy-two compounds have been evaluated for their cytotoxic activity against planes of carcinoma cell lines, whilst the anthraquinones with antibacterial activity were the second on the list with sixty-nine compounds demonstrated bioactivity against a wide range of microorganisms. Meanwhile, an enormous spectrum of further biomedical potentialities exhibited by these compounds as (antioxidant, antiviral, antifungal, immunomodulatory, anti-inflammatory, …….*etc.*) have been documented. Such a massive connection between chemical spaces and bioactivities highlights the huge capacity of marine-derived fungi as an attractive biological source that is worth further exploitations with distinguished anticipations for the global pharmaceuticals industries. Additionally, recent advances in the level of sampling techniques, fermentation, synthetic biology, genetic engineering, genome mining, and total chemical synthesis, all are crucial to the success of fungal MNPs as future drug leads. Furthermore, all reported anthraquinones were extensively investigated for their *in silico* Drug-likeness and pharmacokinetics properties using SWISSADME online platform, which intriguingly highlighted a list of 20 anthraquinone containing compounds (ESI†) that could be considered as potential drug leads scaffolds (Fig. 17 and 18).

## Author contributions

Conceptualization: Amr El-Demerdash. Validation: Amr El-Demerdash. Formal analysis: Mohamed Sebak, Fatma Molham, Claudio Greco Mohamed A. Tammam, Mansour Sobeh and Amr El-Demerdash. Investigation: Mohamed Sebak, Fatma Molham, Claudio Greco Mohamed A. Tammam, Mansour Sobeh and Amr El-Demerdash. Resources: Mohamed Sebak, Fatma Molham, Claudio Greco Mohamed A. Tammam, Mansour Sobeh and Amr El-Demerdash. Data curation: Mohamed Sebak, Fatma Molham, Claudio Greco Mohamed A. Tammam, Mansour Sobeh and Amr El-Demerdash. Writing original draft: Mohamed Sebak, Fatma Molham, Claudio Greco Mohamed A. Tammam, Mansour Sobeh and Amr El-Demerdash. Writing-review & editing: Mohamed Sebak, Fatma Molham, Claudio Greco Mohamed A. Tammam, Mansour Sobeh and Amr El-Demerdash.

## Conflicts of interest

The authors declare that they have no known competing commercial interests or personal relationships that could have appeared to influence the work reported in this paper.

## Funding

Amr El-Demerdash is immensely grateful to the John Innes Centre, Norwich Research Park, United Kingdom for the postdoctoral fellowship. Claudio Greco was supported by the BBSRC (BB/V005723/1).

## List of abbreviations

*A.*
: *Aspergillus*
ACP: acyl carrier protein
AT: acyl transferase
*B*.: Bacillus
BBB: blood–brain barrier
BS: bioavailability score
*C*
: Candida
Cox-2: cyclooxygenase-2
Csp^3^: sp^3^ carbons
DPPH: 1,1-diphenyl-2-picrylhydrazyl
*E*.: Escherichia
ED_50_: median effective dose (the dose which produces a specified effect in 50% of the population in a study)
*Eu*.: Eurotium
*F*.: Fusarium
FCsp^3^: fraction of sp3 carbons
HCV: Hepatitis C virus
GI: Gastrointestinal
KS: ketosynthase
IC_50_: inhibitory concentration that causes a 50% reduction in cell viability
IL: interleukin
LD_50_: lethal dose 50 (the dose which produces death in 50% of the population in a study)
Log P: lipophilicity
Log S: solubility
*M*.: Mycobacterium
MdpF: metallo-hydrolase protein
MIC: minimum inhibitory concentration
MNP: marine natural product
MRSA: methicillin-resistant *Staphylococcus aureus*
Mwt: molecular weight
nrPKS: non-reducing polyketide synthase
*P*.: Penicillium
P-gp: P-glycoprotein
PT: product template
RB: rotatable bond
*S*.: Staphylococcus
SAT: starter unit-ACP transacylase
*Str*.: Streptococcus
*T*.: Trichoderma
Topo: topoisomerase
TPSA: topological polar surface area
*V*.: Vibrio

## Supplementary Material

RA-012-D2RA03610J-s001
