# Supplementary material for: Chemical diversity, medicinal potentialities, biosynthesis, and pharmacokinetics of anthraquinones and their congeners derived from marine fungi: a comprehensive update
Source: RSC Adv. 2022 Sep 1;12(38):24887–921. doi: 10.1039/d2ra03610j (PMC9434105; doi:10.1039/d2ra03610j)
Supplement: RA-012-D2RA03610J-s001 [file RA-012-D2RA03610J-s001.pdf]

Electronic Supplementary Material (ESI) for RSC Advances.
This journal is © The Royal Society of Chemistry 2022

**Supporting information**

# Chemical Diversity, Medicinal Potentialities, Biosynthesis and Pharmacokinetics of Anthraquinones Derived from Marine Fungi: A Comprehensive Update

Mohamed Sebak[1], Fatma Molham[1], Claudio Greco[2] Mohamed A. Tammam[3], Mansour Sobeh[4] and Amr El-Demerdash [5,6*]

[1] Microbiology and Immunology Department, Faculty of Pharmacy, Beni-Suef University, Beni-Suef 62514, Egypt

[2] Molecular Microbiology Department, The John Innes Center, Norwich Research Park, Norwich NR4 7UH, UK

[3] Department of Biochemistry, Faculty of Agriculture, Fayoum University, Fayoum 63514, Egypt

[4] AgroBioSciences Department, Mohammed VI Polytechnic University (UM6P), Ben Guerir, Morocco

[5] Organic Chemistry Division, Department of Chemistry, Faculty of Science, Mansoura University, Mansoura 35516, Egypt

[6] Department of Metabolic Biology and Biological Chemistry, The John Innes Center, Norwich Research Park, Norwich NR4 7UH, UK

* Correspondence: a_eldemerdash83@mans.edu.eg; Amr.El-Demerdash@jic.ac.uk, (A. E-D), Tel: 00447834240424

**Table S1.** Drug-likeness properties of marine-derived fungal anthraquinones. The compounds' numbers are according to the main text.

| Molecule | MW | H-bond acceptors | H-bond donors | Consensus Log P | Fraction Csp3 | RB | TPSA | ESOL Log S | ESOL Class | MR | Bioavailability Score | Lipinski Violations | Ghose Violations | Veber Violations | Egan Violations | Muegge Violations |
|---|---|---|---|---|---|---|---|---|---|---|---|---|---|---|---|---|
| **NIG** | | | | | | | | | | | | | | | | |
| **1** | 596.58 | 12 | 8 | 0.42 | 0.45 | 2 | 222.28 | -3.94 | S | 149.03 | 0.17 | 3 | 3 | 1 | 1 | 3 |
| **2** | 338.35 | 7 | 4 | 0.79 | 0.59 | 2 | 116.45 | -2.39 | S | 85.01 | 0.55 | 0 | 0 | 0 | 0 | 0 |
| **3** | 320.29 | 7 | 4 | 0.17 | 0.38 | 1 | 124.29 | -1.93 | VS | 77.92 | 0.56 | 0 | 0 | 0 | 0 | 0 |
| **4** | 300.26 | 6 | 3 | 1.99 | 0.12 | 1 | 104.06 | -4.08 | MS | 77.28 | 0.55 | 0 | 0 | 0 | 0 | 0 |
| **5** | 284.26 | 5 | 2 | 2.57 | 0.12 | 1 | 83.83 | -4.81 | MS | 75.25 | 0.55 | 0 | 0 | 0 | 0 | 0 |
| **6** | 304.29 | 6 | 3 | 0.91 | 0.38 | 1 | 104.06 | -2.54 | S | 76.76 | 0.56 | 0 | 0 | 0 | 0 | 0 |
| **7** | 324.33 | 7 | 5 | 0.4 | 0.56 | 1 | 127.45 | -2.03 | S | 80.28 | 0.55 | 0 | 0 | 0 | 0 | 0 |
| **8** | 340.33 | 8 | 6 | -0.53 | 0.56 | 1 | 147.68 | -1.51 | VS | 81.44 | 0.55 | 1 | 1 | 1 | 1 | 1 |
| **9** | 320.29 | 7 | 4 | 0.75 | 0.38 | 1 | 124.29 | -2.75 | S | 78.78 | 0.56 | 0 | 0 | 0 | 0 | 0 |
| **10** | 336.29 | 8 | 5 | -0.05 | 0.38 | 1 | 144.52 | -2.14 | S | 79.94 | 0.56 | 0 | 1 | 1 | 1 | 0 |
| **ASP** | | | | | | | | | | | | | | | | |
| 7 | 324.33 | 7 | 5 | 0.4 | 0.56 | 1 | 127.45 | -2.03 | S | 80.28 | 0.55 | 0 | 0 | 0 | 0 | 0 |
| 8 | 340.33 | 8 | 6 | -0.53 | 0.56 | 1 | 147.68 | -1.51 | VS | 81.44 | 0.55 | 1 | 1 | 1 | 1 | 1 |
| 10 | 336.29 | 8 | 5 | -0.05 | 0.38 | 1 | 144.52 | -2.14 | S | 79.94 | 0.56 | 0 | 1 | 1 | 1 | 0 |
| 11 | 460.39 | 9 | 5 | 2.88 | 0.08 | 2 | 165.5 | -5.69 | MS | 121.27 | 0.55 | 0 | 0 | 1 | 1 | 1 |
| 12 | 474.42 | 9 | 4 | 3.14 | 0.12 | 3 | 154.5 | -5.9 | MS | 125.74 | 0.55 | 0 | 0 | 1 | 1 | 1 |
| 13 | 270.24 | 5 | 3 | 1.87 | 0.07 | 0 | 94.83 | -3.67 | S | 70.78 | 0.55 | 0 | 0 | 0 | 0 | 0 |
| 14 | 284.26 | 5 | 2 | 2.27 | 0.12 | 1 | 83.83 | -3.87 | S | 75.25 | 0.55 | 0 | 0 | 0 | 0 | 0 |
| 15 | 288.3 | 5 | 3 | 1.94 | 0.31 | 1 | 86.99 | -3.36 | S | 78.35 | 0.55 | 0 | 0 | 0 | 0 | 0 |
| 16 | 288.3 | 5 | 3 | 1.92 | 0.31 | 1 | 86.99 | -3.43 | S | 78.31 | 0.55 | 0 | 0 | 0 | 0 | 0 |
| 17 | 288.3 | 5 | 3 | 1.92 | 0.31 | 1 | 86.99 | -3.43 | S | 78.31 | 0.55 | 0 | 0 | 0 | 0 | 0 |
| 18 | 286.24 | 6 | 4 | 1.63 | 0.07 | 0 | 115.06 | -3.87 | S | 72.81 | 0.55 | 0 | 0 | 0 | 0 | 0 |
| 19 | 300.26 | 6 | 3 | 1.96 | 0.12 | 1 | 104.06 | -4.08 | MS | 77.28 | 0.55 | 0 | 0 | 0 | 0 | 0 |
| 20 | 402.35 | 9 | 4 | 1.11 | 0.25 | 6 | 150.59 | -3.25 | S | 98.47 | 0.55 | 0 | 0 | 1 | 1 | 1 |
| 21 | 538.54 | 8 | 4 | 4.66 | 0.19 | 3 | 133.52 | -7.47 | PS | 147.55 | 0.55 | 1 | 2 | 0 | 1 | 1 |

| | | | | | | | | | | | | | | | | |
|---|---|---|---|---|---|---|---|---|---|---|---|---|---|---|---|---|
| 22 | 524.52 | 8 | 5 | 4.31 | 0.16 | 2 | 144.52 | -7.26 | PS | 143.08 | 0.55 | 1 | 2 | 1 | 1 | 1 |
| 23 | 524.52 | 8 | 5 | 4.27 | 0.16 | 2 | 144.52 | -7.26 | PS | 143.08 | 0.55 | 1 | 2 | 1 | 1 | 1 |
| 24 | 754.78 | 13 | 6 | 5.9 | 0.33 | 14 | 217.35 | -9.18 | PS | 201.5 | 0.17 | 3 | 4 | 2 | 2 | 5 |
| 25 | 726.72 | 13 | 8 | 5.22 | 0.3 | 12 | 239.35 | -9.17 | PS | 192.04 | 0.17 | 3 | 4 | 2 | 1 | 5 |
| 26 | 372.37 | 7 | 5 | 2.52 | 0.3 | 5 | 135.29 | -4.69 | MS | 98 | 0.55 | 0 | 0 | 0 | 1 | 0 |
| 27 | 386.4 | 7 | 4 | 2.8 | 0.33 | 6 | 124.29 | -4.69 | MS | 102.73 | 0.55 | 0 | 0 | 0 | 0 | 0 |
| 28 | 354.35 | 6 | 4 | 3.01 | 0.2 | 4 | 115.06 | -4.96 | MS | 97.16 | 0.55 | 0 | 0 | 0 | 0 | 0 |
| 29 | 400.42 | 7 | 3 | 3.22 | 0.36 | 7 | 113.29 | -5.12 | MS | 106.94 | 0.55 | 0 | 0 | 0 | 0 | 0 |
| 30 | 368.34 | 7 | 3 | 2.32 | 0.3 | 0 | 113.29 | -4.33 | MS | 93.4 | 0.55 | 0 | 0 | 0 | 0 | 0 |
| 31 | 382.36 | 7 | 2 | 2.74 | 0.33 | 1 | 102.29 | -4.54 | MS | 97.87 | 0.55 | 0 | 0 | 0 | 0 | 0 |
| 32 | 396.39 | 7 | 1 | 3.06 | 0.36 | 2 | 91.29 | -4.76 | MS | 102.34 | 0.55 | 0 | 0 | 0 | 0 | 0 |
| 33 | 340.28 | 7 | 3 | 1.7 | 0.22 | 0 | 113.29 | -3.75 | S | 83.75 | 0.55 | 0 | 0 | 0 | 0 | 0 |
| 34 | 356.28 | 8 | 4 | 0.87 | 0.22 | 0 | 133.52 | -3.22 | S | 84.91 | 0.55 | 0 | 0 | 0 | 1 | 0 |
| 36 | 356.33 | 7 | 4 | 1.84 | 0.26 | 1 | 124.29 | -3.92 | S | 91.01 | 0.55 | 0 | 0 | 0 | 0 | 0 |
| 37 | 384.34 | 8 | 4 | 1.52 | 0.3 | 0 | 133.52 | -3.8 | S | 94.56 | 0.55 | 0 | 0 | 0 | 1 | 0 |
| 38 | 412.39 | 8 | 2 | 2.26 | 0.36 | 2 | 111.52 | -4.23 | MS | 103.5 | 0.55 | 0 | 0 | 0 | 0 | 0 |
| 39 | 360.31 | 8 | 6 | 0.79 | 0.22 | 4 | 155.52 | -2.81 | S | 89.55 | 0.55 | 1 | 0 | 1 | 1 | 2 |
| 40 | 388.37 | 8 | 4 | 1.52 | 0.3 | 6 | 133.52 | -3.23 | S | 98.49 | 0.55 | 0 | 0 | 0 | 1 | 0 |
| 41 | 284.26 | 5 | 2 | 2.19 | 0.12 | 1 | 83.83 | -3.87 | S | 75.25 | 0.55 | 0 | 0 | 0 | 0 | 0 |
| 42 | 328.32 | 6 | 3 | 1.97 | 0.22 | 3 | 104.06 | -3.74 | S | 86.03 | 0.55 | 0 | 0 | 0 | 0 | 0 |
| 43 | 368.34 | 7 | 1 | 2.42 | 0.3 | 2 | 91.29 | -4.18 | MS | 92.69 | 0.55 | 0 | 0 | 0 | 0 | 0 |
| 44 | 366.32 | 7 | 1 | 2.42 | 0.2 | 2 | 91.29 | -4.36 | MS | 92.21 | 0.55 | 0 | 0 | 0 | 0 | 0 |
| 45 | 300.26 | 6 | 3 | 1.86 | 0.12 | 1 | 104.06 | -3.73 | S | 77.28 | 0.55 | 0 | 0 | 0 | 0 | 0 |
| 46 | 314.29 | 6 | 2 | 2.23 | 0.18 | 2 | 93.06 | -3.95 | S | 81.75 | 0.55 | 0 | 0 | 0 | 0 | 0 |
| 47 | 238.24 | 3 | 1 | 2.72 | 0.07 | 0 | 54.37 | -4.25 | MS | 66.74 | 0.55 | 0 | 0 | 0 | 0 | 0 |
| 48 | 298.29 | 5 | 1 | 2.26 | 0.18 | 3 | 72.83 | -3.6 | S | 78.52 | 0.55 | 0 | 0 | 0 | 0 | 0 |
| 49 | 222.24 | 2 | 0 | 3.01 | 0.07 | 0 | 34.14 | -4.22 | MS | 64.72 | 0.55 | 0 | 0 | 0 | 0 | 0 |
| 50 | 282.25 | 5 | 1 | 1.84 | 0.06 | 2 | 80.67 | -3.46 | S | 73.65 | 0.55 | 0 | 0 | 0 | 0 | 0 |
| 51 | 240.21 | 4 | 2 | 1.98 | 0 | 0 | 74.6 | -3.81 | S | 63.8 | 0.55 | 0 | 0 | 0 | 0 | 0 |
| 52 | 254.24 | 4 | 2 | 2.23 | 0.07 | 0 | 74.6 | -3.82 | S | 68.76 | 0.55 | 0 | 0 | 0 | 0 | 0 |
| 53 | 270.24 | 5 | 3 | 1.5 | 0.07 | 0 | 94.83 | -3.67 | S | 70.78 | 0.55 | 0 | 0 | 0 | 0 | 0 |

| | | | | | | | | | | | | | | | | |
|---|---|---|---|---|---|---|---|---|---|---|---|---|---|---|---|---|
| 54 | 284.26 | 5 | 2 | 1.98 | 0.12 | 1 | 83.83 | -3.53 | S | 75.25 | 0.55 | 0 | 0 | 0 | 0 | 0 |
| 55 | 316.26 | 7 | 3 | 1.52 | 0.12 | 2 | 117.2 | -3.27 | S | 82.93 | 0.55 | 0 | 0 | 0 | 0 | 0 |
| 56 | 270.24 | 5 | 3 | 2 | 0.07 | 0 | 94.83 | -4.02 | MS | 70.78 | 0.55 | 0 | 0 | 0 | 0 | 0 |
| 57 | 298.29 | 5 | 1 | 2.6 | 0.18 | 2 | 72.83 | -4.08 | MS | 79.72 | 0.55 | 0 | 0 | 0 | 0 | 0 |
| 58 | 284.26 | 5 | 2 | 2.43 | 0.12 | 1 | 83.83 | -4.23 | MS | 75.25 | 0.55 | 0 | 0 | 0 | 0 | 0 |
| 59 | 288.25 | 6 | 4 | 1.95 | 0.13 | 0 | 107.22 | -3.83 | S | 73.91 | 0.55 | 0 | 0 | 0 | 0 | 0 |
| 60 | 302.28 | 6 | 3 | 2.34 | 0.19 | 1 | 96.22 | -4.04 | MS | 78.38 | 0.55 | 0 | 0 | 0 | 0 | 0 |
| 61 | 388.32 | 9 | 5 | 0.87 | 0.21 | 5 | 161.59 | -3.38 | S | 94 | 0.55 | 0 | 0 | 1 | 1 | 1 |
| 62 | 300.26 | 6 | 3 | 1.42 | 0.12 | 2 | 104.06 | -3.1 | S | 76.41 | 0.55 | 0 | 0 | 0 | 0 | 0 |
| 63 | 300.26 | 6 | 3 | 1.5 | 0.12 | 2 | 104.06 | -3.1 | S | 76.41 | 0.55 | 0 | 0 | 0 | 0 | 0 |
| 64 | 300.26 | 6 | 3 | 1.97 | 0.12 | 1 | 104.06 | -4.08 | MS | 77.28 | 0.55 | 0 | 0 | 0 | 0 | 0 |
| 65 | 340.28 | 7 | 3 | 1.7 | 0.22 | 0 | 113.29 | -3.75 | S | 83.75 | 0.55 | 0 | 0 | 0 | 0 | 0 |
| 66 | 340.28 | 7 | 3 | 1.6 | 0.22 | 0 | 113.29 | -3.75 | S | 83.75 | 0.55 | 0 | 0 | 0 | 0 | 0 |
| 67 | 370.35 | 7 | 4 | 2.46 | 0.25 | 5 | 132.13 | -4.74 | MS | 97.26 | 0.55 | 0 | 0 | 0 | 1 | 0 |
| 68 | 386.4 | 7 | 4 | 2.94 | 0.33 | 6 | 124.29 | -4.9 | MS | 102.47 | 0.55 | 0 | 0 | 0 | 0 | 0 |
| 69 | 400.42 | 7 | 3 | 3.3 | 0.36 | 7 | 113.29 | -4.9 | MS | 107.2 | 0.55 | 0 | 0 | 0 | 0 | 0 |
| 70 | 428.47 | 7 | 4 | 3.9 | 0.42 | 9 | 124.29 | -5.51 | MS | 117.15 | 0.55 | 0 | 0 | 0 | 0 | 1 |
| 71 | 406.81 | 7 | 5 | 3.02 | 0.3 | 5 | 135.29 | -5.29 | MS | 103.01 | 0.55 | 0 | 0 | 0 | 1 | 0 |
| 72 | 420.84 | 7 | 4 | 3.42 | 0.33 | 6 | 124.29 | -5.5 | MS | 107.48 | 0.55 | 0 | 0 | 0 | 0 | 0 |
| 73 | 420.84 | 7 | 4 | 3.42 | 0.33 | 6 | 124.29 | -5.29 | MS | 107.74 | 0.55 | 0 | 0 | 0 | 0 | 0 |
| 74 | 434.87 | 7 | 3 | 3.81 | 0.36 | 7 | 113.29 | -5.5 | MS | 112.21 | 0.55 | 0 | 0 | 0 | 0 | 0 |
| 75 | 462.92 | 7 | 4 | 4.41 | 0.42 | 9 | 124.29 | -6.11 | PS | 122.16 | 0.55 | 0 | 0 | 0 | 0 | 1 |
| 76 | 388.8 | 6 | 4 | 3.55 | 0.2 | 4 | 115.06 | -5.56 | MS | 102.17 | 0.55 | 0 | 0 | 0 | 0 | 1 |
| 77 | 402.82 | 6 | 3 | 3.94 | 0.24 | 5 | 104.06 | -5.78 | MS | 106.64 | 0.55 | 0 | 0 | 0 | 0 | 1 |
| 78 | 465.29 | 7 | 4 | 3.44 | 0.33 | 6 | 124.29 | -5.82 | MS | 110.17 | 0.55 | 0 | 0 | 0 | 0 | 1 |
| 79 | 479.32 | 7 | 3 | 3.9 | 0.36 | 7 | 113.29 | -5.82 | MS | 114.9 | 0.55 | 0 | 0 | 0 | 0 | 1 |
| 80 | 414.45 | 7 | 2 | 3.54 | 0.39 | 8 | 102.29 | -5.12 | MS | 111.67 | 0.55 | 0 | 0 | 0 | 0 | 0 |
| **PEN** | | | | | | | | | | | | | | | | |
| 13 | 270.24 | 5 | 3 | 1.87 | 0.07 | 0 | 94.83 | -3.67 | S | 70.78 | 0.55 | 0 | 0 | 0 | 0 | 0 |

| | | | | | | | | | | | | | | | | |
|---|---|---|---|---|---|---|---|---|---|---|---|---|---|---|---|---|
| 17 | 288.3 | 5 | 3 | 1.92 | 0.31 | 1 | 86.99 | -3.43 | S | 78.31 | 0.55 | 0 | 0 | 0 | 0 | 0 |
| 26 | 372.37 | 7 | 5 | 2.52 | 0.3 | 5 | 135.29 | -4.69 | MS | 98 | 0.55 | 0 | 0 | 0 | 1 | 0 |
| 27 | 386.4 | 7 | 4 | 2.8 | 0.33 | 6 | 124.29 | -4.69 | MS | 102.73 | 0.55 | 0 | 0 | 0 | 0 | 0 |
| 37 | 384.34 | 8 | 4 | 1.52 | 0.3 | 0 | 133.52 | -3.8 | S | 94.56 | 0.55 | 0 | 0 | 0 | 1 | 0 |
| 81 | 520.48 | 10 | 6 | 2.96 | 0.25 | 2 | 181.82 | -6.52 | PS | 134.57 | 0.11 | 2 | 2 | 1 | 1 | 3 |
| 82 | 536.48 | 11 | 7 | 2.25 | 0.25 | 3 | 202.05 | -5.76 | MS | 135.73 | 0.11 | 3 | 2 | 1 | 1 | 3 |
| 83 | 254.24 | 4 | 2 | 2.38 | 0.07 | 0 | 74.6 | -4.11 | MS | 68.76 | 0.55 | 0 | 0 | 0 | 0 | 0 |
| 84 | 286.24 | 6 | 4 | 1.08 | 0.07 | 1 | 115.06 | -2.9 | S | 71.95 | 0.55 | 0 | 0 | 0 | 0 | 0 |
| 85 | 376.74 | 7 | 2 | 2.46 | 0.17 | 4 | 110.13 | -4.14 | MS | 91.16 | 0.55 | 0 | 0 | 0 | 0 | 0 |
| 86 | 366.3 | 9 | 4 | 0.44 | 0.07 | 3 | 166.81 | -2.82 | S | 82.14 | 0.11 | 0 | 0 | 1 | 1 | 1 |
| 87 | 350.3 | 8 | 3 | 1.29 | 0.07 | 2 | 146.58 | -3.59 | S | 80.98 | 0.56 | 0 | 0 | 1 | 1 | 0 |
| 88 | 270.24 | 5 | 3 | 1.5 | 0.07 | 1 | 94.83 | -3.04 | S | 69.92 | 0.55 | 0 | 0 | 0 | 0 | 0 |
| 89 | 413.38 | 8 | 4 | 1.79 | 0.24 | 6 | 150.23 | -4.14 | MS | 104.13 | 0.55 | 0 | 0 | 1 | 1 | 1 |
| 90 | 399.35 | 8 | 5 | 1.52 | 0.2 | 5 | 161.23 | -3.92 | S | 99.81 | 0.11 | 0 | 0 | 1 | 1 | 1 |
| 91 | 433.8 | 8 | 5 | 1.95 | 0.2 | 5 | 161.23 | -4.52 | MS | 104.82 | 0.11 | 0 | 0 | 1 | 1 | 1 |
| 92 | 427.4 | 8 | 4 | 2.11 | 0.27 | 7 | 150.23 | -4.37 | MS | 108.94 | 0.55 | 0 | 0 | 1 | 1 | 1 |
| 93 | 413.38 | 8 | 5 | 1.73 | 0.24 | 6 | 161.23 | -4.16 | MS | 104.62 | 0.11 | 0 | 0 | 1 | 1 | 1 |
| 94 | 447.82 | 8 | 5 | 2.27 | 0.24 | 6 | 161.23 | -4.76 | MS | 109.63 | 0.11 | 0 | 0 | 1 | 1 | 1 |
| 95 | 447.82 | 8 | 5 | 2.23 | 0.24 | 6 | 161.23 | -4.76 | MS | 109.63 | 0.11 | 0 | 0 | 1 | 1 | 1 |
| 96 | 371.3 | 8 | 5 | 0.84 | 0.11 | 4 | 161.23 | -3.23 | S | 90.2 | 0.11 | 0 | 0 | 1 | 1 | 1 |
| 97 | 300.22 | 7 | 4 | 1.07 | 0 | 1 | 132.13 | -3.22 | S | 72.78 | 0.56 | 0 | 0 | 0 | 1 | 0 |
| 98 | 320.68 | 6 | 4 | 1.62 | 0.07 | 1 | 115.06 | -3.49 | S | 76.96 | 0.55 | 0 | 0 | 0 | 0 | 0 |

| **STE** | | | | | | | | | | | | | | | | |
|---|---|---|---|---|---|---|---|---|---|---|---|---|---|---|---|---|
| 99 | 336.29 | 8 | 5 | -0.67 | 0.38 | 1 | 144.52 | -1.2 | VS | 79.36 | 0.55 | 0 | 1 | 1 | 1 | 0 |
| 100 | 322.31 | 7 | 5 | -0.11 | 0.44 | 1 | 127.45 | -1.49 | VS | 78.94 | 0.55 | 0 | 1 | 0 | 0 | 0 |
| 101 | 304.29 | 6 | 3 | 1.11 | 0.38 | 1 | 104.06 | -2.42 | S | 77.04 | 0.55 | 0 | 0 | 0 | 0 | 0 |
| 102 | 308.33 | 6 | 4 | 0.72 | 0.56 | 1 | 107.22 | -2.17 | S | 78.25 | 0.55 | 0 | 0 | 0 | 0 | 0 |
| 103 | 346.33 | 7 | 2 | 1.55 | 0.39 | 3 | 110.13 | -2.88 | S | 86.78 | 0.55 | 0 | 0 | 0 | 0 | 0 |

| | | | | | | | | | | | | | | | | |
|---|---|---|---|---|---|---|---|---|---|---|---|---|---|---|---|---|
| 104 | 320.29 | 7 | 4 | 0.24 | 0.38 | 1 | 124.29 | -1.81 | VS | 78.2 | 0.55 | 0 | 0 | 0 | 0 | 0 |
| 105 | 324.33 | 7 | 5 | -0.22 | 0.56 | 1 | 127.45 | -1.64 | VS | 79.42 | 0.55 | 0 | 1 | 0 | 0 | 0 |
| 106 | 366.36 | 8 | 4 | 0.31 | 0.56 | 3 | 133.52 | -2.12 | S | 89.15 | 0.55 | 0 | 0 | 0 | 1 | 0 |
| 107 | 340.33 | 8 | 6 | -0.91 | 0.56 | 1 | 147.68 | -1.12 | VS | 80.58 | 0.55 | 1 | 1 | 1 | 1 | 1 |
| 108 | 284.26 | 5 | 2 | 2.22 | 0.12 | 1 | 83.83 | -3.87 | S | 75.25 | 0.55 | 0 | 0 | 0 | 0 | 0 |
| 109 | 364.33 | 8 | 2 | 1.65 | 0.12 | 3 | 135.58 | -3.8 | S | 85.45 | 0.56 | 0 | 0 | 0 | 1 | 0 |
| 110 | 488.44 | 11 | 4 | 0.93 | 0.38 | 6 | 169.05 | -3.52 | S | 117.11 | 0.55 | 1 | 1 | 1 | 1 | 2 |
| 111 | 352.29 | 9 | 5 | -0.9 | 0.5 | 1 | 157.05 | -1.06 | VS | 78.89 | 0.55 | 0 | 1 | 1 | 1 | 1 |
| 112 | 618.54 | 13 | 7 | 1.08 | 0.25 | 3 | 228.35 | -4.65 | MS | 153.61 | 0.17 | 3 | 3 | 1 | 1 | 4 |
| 113 | 618.54 | 13 | 7 | 1.08 | 0.25 | 3 | 228.35 | -4.65 | MS | 153.61 | 0.17 | 3 | 3 | 1 | 1 | 4 |
| 114 | 566.51 | 10 | 4 | 3.95 | 0.12 | 3 | 167.66 | -7.22 | PS | 149.5 | 0.55 | 1 | 2 | 1 | 1 | 2 |
| 115 | 670.57 | 16 | 10 | -1.82 | 0.38 | 3 | 289.04 | -2.11 | S | 157.72 | 0.17 | 3 | 4 | 1 | 1 | 5 |
| 116 | 670.57 | 16 | 10 | -1.82 | 0.38 | 3 | 289.04 | -2.11 | S | 157.72 | 0.17 | 3 | 4 | 1 | 1 | 5 |
| 117 | 618.54 | 13 | 7 | 1.02 | 0.25 | 3 | 228.35 | -4.65 | MS | 153.61 | 0.17 | 3 | 3 | 1 | 1 | 4 |
| 118 | 566.51 | 10 | 4 | 3.94 | 0.12 | 3 | 167.66 | -7.22 | PS | 149.5 | 0.55 | 1 | 2 | 1 | 1 | 2 |
| 119 | 566.51 | 10 | 4 | 3.95 | 0.12 | 3 | 167.66 | -7.22 | PS | 149.5 | 0.55 | 1 | 2 | 1 | 1 | 2 |
| 120 | 622.57 | 13 | 7 | 0.74 | 0.38 | 3 | 228.35 | -3.92 | S | 154.24 | 0.17 | 3 | 3 | 1 | 1 | 4 |
| 121 | 606.57 | 12 | 6 | 1.64 | 0.38 | 3 | 208.12 | -4.52 | MS | 153.08 | 0.17 | 3 | 3 | 1 | 1 | 4 |
| 122 | 566.51 | 10 | 4 | 3.86 | 0.12 | 3 | 167.66 | -7.22 | PS | 149.5 | 0.55 | 1 | 2 | 1 | 1 | 2 |
| 123 | 602.54 | 12 | 6 | 2 | 0.25 | 3 | 208.12 | -5.27 | MS | 152.45 | 0.17 | 3 | 2 | 1 | 1 | 4 |
| 124 | 602.54 | 12 | 6 | 1.98 | 0.25 | 3 | 208.12 | -5.27 | MS | 152.45 | 0.17 | 3 | 2 | 1 | 1 | 4 |
| 125 | 618.54 | 13 | 7 | 1.19 | 0.25 | 3 | 228.35 | -4.65 | MS | 153.61 | 0.17 | 3 | 3 | 1 | 1 | 4 |
| 126 | 618.54 | 13 | 7 | 1 | 0.25 | 3 | 228.35 | -4.65 | MS | 153.61 | 0.17 | 3 | 3 | 1 | 1 | 4 |
| 127 | 618.54 | 13 | 7 | 1 | 0.25 | 3 | 228.35 | -4.65 | MS | 153.61 | 0.17 | 3 | 3 | 1 | 1 | 4 |
| 128 | 632.57 | 13 | 6 | 1.43 | 0.27 | 4 | 217.35 | -5.01 | MS | 158.34 | 0.17 | 3 | 3 | 1 | 1 | 4 |
| 129 | 446.4 | 10 | 5 | 0.64 | 0.36 | 4 | 162.98 | -3.4 | S | 107.38 | 0.55 | 0 | 0 | 1 | 1 | 1 |
| 130 | 606.57 | 12 | 6 | 1.74 | 0.38 | 3 | 208.12 | -4.52 | MS | 153.08 | 0.17 | 3 | 3 | 1 | 1 | 4 |

**ALT**

| | | | | | | | | | | | | | | | | |
|---|---|---|---|---|---|---|---|---|---|---|---|---|---|---|---|---|
| 100 | 322.31 | 7 | 5 | -0.11 | 0.44 | 1 | 127.45 | -1.49 | VS | 78.94 | 0.55 | 0 | 1 | 0 | 0 | 0 |
| 101 | 304.29 | 6 | 3 | 1.11 | 0.38 | 1 | 104.06 | -2.42 | S | 77.04 | 0.55 | 0 | 0 | 0 | 0 | 0 |
| 102 | 308.33 | 6 | 4 | 0.72 | 0.56 | 1 | 107.22 | -2.17 | S | 78.25 | 0.55 | 0 | 0 | 0 | 0 | 0 |
| 104 | 320.29 | 7 | 4 | 0.24 | 0.38 | 1 | 124.29 | -1.81 | VS | 78.2 | 0.55 | 0 | 0 | 0 | 0 | 0 |
| 105 | 324.33 | 7 | 5 | -0.22 | 0.56 | 1 | 127.45 | -1.64 | VS | 79.42 | 0.55 | 0 | 1 | 0 | 0 | 0 |
| 107 | 340.33 | 8 | 6 | -0.91 | 0.56 | 1 | 147.68 | -1.12 | VS | 80.58 | 0.55 | 1 | 1 | 1 | 1 | 1 |
| 108 | 284.26 | 5 | 2 | 2.22 | 0.12 | 1 | 83.83 | -3.87 | S | 75.25 | 0.55 | 0 | 0 | 0 | 0 | 0 |
| 114 | 566.51 | 10 | 4 | 3.95 | 0.12 | 3 | 167.66 | -7.22 | PS | 149.5 | 0.55 | 1 | 2 | 1 | 1 | 2 |
| 131 | 586.54 | 11 | 5 | 2.66 | 0.25 | 3 | 187.89 | -5.71 | MS | 151.29 | 0.17 | 2 | 2 | 1 | 1 | 2 |
| 132 | 602.54 | 12 | 6 | 1.7 | 0.25 | 3 | 208.12 | -5.27 | MS | 152.45 | 0.17 | 3 | 2 | 1 | 1 | 4 |
| 133 | 602.54 | 12 | 6 | 1.96 | 0.25 | 3 | 208.12 | -5.27 | MS | 152.45 | 0.17 | 3 | 2 | 1 | 1 | 4 |
| 134 | 638.57 | 14 | 8 | -0.29 | 0.38 | 3 | 248.58 | -3.09 | S | 154.29 | 0.17 | 3 | 4 | 1 | 1 | 4 |
| 135 | 602.54 | 12 | 6 | 1.62 | 0.25 | 3 | 208.12 | -5.27 | MS | 152.45 | 0.17 | 3 | 2 | 1 | 1 | 4 |
| 136 | 308.33 | 6 | 4 | 0.78 | 0.56 | 1 | 107.22 | -2.17 | S | 78.25 | 0.55 | 0 | 0 | 0 | 0 | 0 |
| 137 | 308.33 | 6 | 4 | 0.72 | 0.56 | 1 | 107.22 | -2.17 | S | 78.25 | 0.55 | 0 | 0 | 0 | 0 | 0 |
| 138 | 308.33 | 6 | 4 | 0.73 | 0.56 | 1 | 107.22 | -2.17 | S | 78.25 | 0.55 | 0 | 0 | 0 | 0 | 0 |
| 139 | 350.36 | 7 | 3 | 1.18 | 0.56 | 3 | 113.29 | -2.63 | S | 87.99 | 0.55 | 0 | 0 | 0 | 0 | 0 |
| 140 | 488.48 | 10 | 5 | 0.39 | 0.6 | 1 | 162.98 | -2.51 | S | 118.47 | 0.11 | 0 | 1 | 1 | 1 | 1 |
| 141 | 300.26 | 6 | 3 | 1.92 | 0.12 | 1 | 104.06 | -3.73 | S | 77.28 | 0.55 | 0 | 0 | 0 | 0 | 0 |
| 142 | 288.3 | 5 | 2 | 1.88 | 0.38 | 1 | 83.83 | -2.94 | S | 75.88 | 0.55 | 0 | 0 | 0 | 0 | 0 |

| **TRI** | | | | | | | | | | | | | | | | |
|---|---|---|---|---|---|---|---|---|---|---|---|---|---|---|---|---|
| 13 | 270.24 | 5 | 3 | 1.87 | 0.07 | 0 | 94.83 | -3.67 | S | 70.78 | 0.55 | 0 | 0 | 0 | 0 | 0 |
| 83 | 254.24 | 4 | 2 | 2.38 | 0.07 | 0 | 74.6 | -4.11 | MS | 68.76 | 0.55 | 0 | 0 | 0 | 0 | 0 |
| 84 | 286.24 | 6 | 4 | 1.08 | 0.07 | 1 | 115.06 | -2.9 | S | 71.95 | 0.55 | 0 | 0 | 0 | 0 | 0 |
| 143 | 276.28 | 5 | 4 | 0.55 | 0.4 | 0 | 97.99 | -2.01 | S | 71.41 | 0.55 | 0 | 0 | 0 | 0 | 0 |
| 144 | 276.28 | 5 | 4 | 0.54 | 0.4 | 0 | 97.99 | -2.01 | S | 71.41 | 0.55 | 0 | 0 | 0 | 0 | 0 |
| 145 | 254.24 | 4 | 2 | 2.22 | 0.07 | 0 | 74.6 | -3.82 | S | 68.76 | 0.55 | 0 | 0 | 0 | 0 | 0 |

| | | | | | | | | | | | | | | | | |
|---|---|---|---|---|---|---|---|---|---|---|---|---|---|---|---|---|
| 146 | 270.24 | 5 | 3 | 1.43 | 0.07 | 1 | 94.83 | -3.04 | S | 69.92 | 0.55 | 0 | 0 | 0 | 0 | 0 |
| 147 | 238.24 | 3 | 1 | 2.73 | 0.07 | 0 | 54.37 | -4.25 | MS | 66.74 | 0.55 | 0 | 0 | 0 | 0 | 0 |
| 148 | 314.29 | 6 | 4 | 1.68 | 0.18 | 2 | 115.06 | -3.53 | S | 81.56 | 0.55 | 0 | 0 | 0 | 0 | 0 |
| 149 | 254.24 | 4 | 2 | 1.94 | 0.07 | 1 | 74.6 | -3.47 | S | 67.9 | 0.55 | 0 | 0 | 0 | 0 | 0 |
| 150 | 290.27 | 6 | 4 | 0.28 | 0.33 | 0 | 115.06 | -1.84 | VS | 71.83 | 0.55 | 0 | 0 | 0 | 0 | 0 |
| 151 | 290.27 | 6 | 4 | 0.29 | 0.33 | 1 | 115.06 | -1.68 | VS | 71.83 | 0.55 | 0 | 0 | 0 | 0 | 0 |
| 152 | 254.24 | 4 | 2 | 2.4 | 0.07 | 0 | 74.6 | -4.16 | MS | 68.76 | 0.55 | 0 | 0 | 0 | 0 | 0 |
| 153 | 254.24 | 4 | 1 | 2.39 | 0.07 | 1 | 63.6 | -4.02 | MS | 68.26 | 0.55 | 0 | 0 | 0 | 0 | 0 |
| 154 | 276.28 | 5 | 4 | 0.52 | 0.4 | 0 | 97.99 | -2.01 | S | 71.41 | 0.55 | 0 | 0 | 0 | 0 | 0 |
| 155 | 290.27 | 6 | 4 | 0.28 | 0.33 | 0 | 115.06 | -1.84 | VS | 71.83 | 0.55 | 0 | 0 | 0 | 0 | 0 |

| **EUR** | | | | | | | | | | | | | | | | |
|---|---|---|---|---|---|---|---|---|---|---|---|---|---|---|---|---|
| 13 | 270.24 | 5 | 3 | 1.87 | 0.07 | 0 | 94.83 | -3.67 | S | 70.78 | 0.55 | 0 | 0 | 0 | 0 | 0 |
| 15 | 288.3 | 5 | 3 | 1.94 | 0.31 | 1 | 86.99 | -3.36 | S | 78.35 | 0.55 | 0 | 0 | 0 | 0 | 0 |
| 18 | 286.24 | 6 | 4 | 1.63 | 0.07 | 0 | 115.06 | -3.87 | S | 72.81 | 0.55 | 0 | 0 | 0 | 0 | 0 |
| 19 | 300.26 | 6 | 3 | 1.96 | 0.12 | 1 | 104.06 | -4.08 | MS | 77.28 | 0.55 | 0 | 0 | 0 | 0 | 0 |
| 20 | 402.35 | 9 | 4 | 1.11 | 0.25 | 6 | 150.59 | -3.25 | S | 98.47 | 0.55 | 0 | 0 | 1 | 1 | 1 |
| 60 | 302.28 | 6 | 3 | 2.34 | 0.19 | 1 | 96.22 | -4.04 | MS | 78.38 | 0.55 | 0 | 0 | 0 | 0 | 0 |
| 63 | 300.26 | 6 | 3 | 1.5 | 0.12 | 2 | 104.06 | -3.1 | S | 76.41 | 0.55 | 0 | 0 | 0 | 0 | 0 |
| 97 | 300.22 | 7 | 4 | 1.07 | 0 | 1 | 132.13 | -3.22 | S | 72.78 | 0.56 | 0 | 0 | 0 | 1 | 0 |
| 154 | 276.28 | 5 | 4 | 0.52 | 0.4 | 0 | 97.99 | -2.01 | S | 71.41 | 0.55 | 0 | 0 | 0 | 0 | 0 |
| 156 | 272.25 | 5 | 3 | 2.34 | 0.13 | 0 | 86.99 | -3.98 | S | 71.89 | 0.55 | 0 | 0 | 0 | 0 | 0 |
| 157 | 286.28 | 5 | 2 | 2.7 | 0.19 | 1 | 75.99 | -4.18 | MS | 76.36 | 0.55 | 0 | 0 | 0 | 0 | 0 |
| 158 | 418.39 | 9 | 4 | 1.49 | 0.38 | 4 | 134.91 | -3.93 | S | 102.51 | 0.55 | 0 | 0 | 0 | 1 | 0 |
| 159 | 416.38 | 9 | 4 | 1.02 | 0.33 | 4 | 142.75 | -3.63 | S | 101.41 | 0.55 | 0 | 0 | 1 | 1 | 0 |
| 160 | 588.6 | 10 | 6 | 3.59 | 0.33 | 4 | 173.98 | -6.46 | PS | 159.7 | 0.17 | 2 | 3 | 1 | 1 | 2 |
| 161 | 432.38 | 10 | 5 | 0.25 | 0.33 | 5 | 162.98 | -2.86 | S | 102.57 | 0.55 | 0 | 1 | 1 | 1 | 1 |
| 162 | 420.41 | 9 | 5 | 0.81 | 0.48 | 4 | 145.91 | -3.13 | S | 104.5 | 0.55 | 0 | 0 | 1 | 1 | 0 |

| | | | | | | | | | | | | | | | | |
|---|---|---|---|---|---|---|---|---|---|---|---|---|---|---|---|---|
| 163 | 276.28 | 5 | 4 | 0.51 | 0.4 | 0 | 97.99 | -2.01 | S | 71.41 | 0.55 | 0 | 0 | 0 | 0 | 0 |

| | | | | | | | | | | | | | | | | |
|---|---|---|---|---|---|---|---|---|---|---|---|---|---|---|---|---|
| **FUS** | | | | | | | | | | | | | | | | |
| 5 | 284.26 | 5 | 2 | 2.57 | 0.12 | 1 | 83.83 | -4.81 | MS | 75.25 | 0.55 | 0 | 0 | 0 | 0 | 0 |
| 6 | 304.29 | 6 | 3 | 0.91 | 0.38 | 1 | 104.06 | -2.54 | S | 76.76 | 0.56 | 0 | 0 | 0 | 0 | 0 |
| 7 | 324.33 | 7 | 5 | 0.4 | 0.56 | 1 | 127.45 | -2.03 | S | 80.28 | 0.55 | 0 | 0 | 0 | 0 | 0 |
| 8 | 340.33 | 8 | 6 | -0.53 | 0.56 | 1 | 147.68 | -1.51 | VS | 81.44 | 0.55 | 1 | 1 | 1 | 1 | 1 |
| 10 | 336.29 | 8 | 5 | -0.05 | 0.38 | 1 | 144.52 | -2.14 | S | 79.94 | 0.56 | 0 | 1 | 1 | 1 | 0 |
| 164 | 304.29 | 6 | 3 | 0.97 | 0.38 | 1 | 104.06 | -2.54 | S | 76.76 | 0.56 | 0 | 0 | 0 | 0 | 0 |
| 165 | 324.33 | 7 | 5 | 0.37 | 0.56 | 1 | 127.45 | -2.03 | S | 80.28 | 0.55 | 0 | 0 | 0 | 0 | 0 |
| 166 | 306.31 | 6 | 3 | 1.24 | 0.56 | 1 | 96.22 | -2.56 | S | 76.93 | 0.55 | 0 | 0 | 0 | 0 | 0 |
| 167 | 340.33 | 8 | 6 | -0.43 | 0.56 | 1 | 147.68 | -1.51 | VS | 81.44 | 0.55 | 1 | 1 | 1 | 1 | 1 |
| 168 | 324.33 | 7 | 5 | 0.36 | 0.56 | 1 | 127.45 | -2.03 | S | 80.28 | 0.55 | 0 | 0 | 0 | 0 | 0 |
| 169 | 352.38 | 5 | 0 | 3.21 | 0.29 | 5 | 69.67 | -3.98 | S | 97.29 | 0.55 | 0 | 0 | 0 | 0 | 0 |
| 170 | 328.27 | 7 | 3 | 1.68 | 0.12 | 2 | 121.13 | -4.07 | MS | 82.5 | 0.55 | 0 | 0 | 0 | 0 | 0 |

| | | | | | | | | | | | | | | | | |
|---|---|---|---|---|---|---|---|---|---|---|---|---|---|---|---|---|
| **ENG** | | | | | | | | | | | | | | | | |
| 171 | 616.57 | 12 | 5 | 2.53 | 0.33 | 4 | 197.12 | -5.66 | MS | 153.75 | 0.11 | 2 | 3 | 1 | 1 | 3 |
| 172 | 616.57 | 12 | 5 | 2.57 | 0.33 | 4 | 197.12 | -5.66 | MS | 153.75 | 0.11 | 2 | 3 | 1 | 1 | 3 |
| 173 | 616.57 | 12 | 5 | 2.61 | 0.33 | 4 | 197.12 | -5.66 | MS | 153.75 | 0.11 | 2 | 3 | 1 | 1 | 3 |
| 174 | 616.57 | 12 | 3 | 2.85 | 0.36 | 5 | 182.96 | -5.44 | MS | 152.77 | 0.11 | 2 | 3 | 1 | 1 | 3 |
| 175 | 634.58 | 13 | 5 | 2.15 | 0.36 | 8 | 214.19 | -4.86 | MS | 156.54 | 0.11 | 2 | 3 | 1 | 1 | 3 |
| 176 | 634.58 | 13 | 5 | 2.34 | 0.36 | 8 | 214.19 | -4.86 | MS | 156.54 | 0.11 | 2 | 3 | 1 | 1 | 3 |
| 177 | 616.57 | 12 | 3 | 2.88 | 0.36 | 5 | 182.96 | -5.44 | MS | 152.77 | 0.11 | 2 | 3 | 1 | 1 | 3 |

| | | | | | | | | | | | | | | | | |
|---|---|---|---|---|---|---|---|---|---|---|---|---|---|---|---|---|
| **SPO** | | | | | | | | | | | | | | | | |
| 101 | 304.29 | 6 | 3 | 1.11 | 0.38 | 1 | 104.06 | -2.42 | S | 77.04 | 0.55 | 0 | 0 | 0 | 0 | 0 |
| 178 | 320.29 | 7 | 4 | 0.26 | 0.38 | 1 | 124.29 | -1.81 | VS | 78.2 | 0.55 | 0 | 0 | 0 | 0 | 0 |
| 179 | 338.31 | 8 | 5 | -0.39 | 0.5 | 1 | 144.52 | -1.46 | VS | 79.88 | 0.55 | 0 | 1 | 1 | 1 | 0 |
| 180 | 356.32 | 9 | 7 | -1.43 | 0.56 | 1 | 167.91 | -0.68 | VS | 81.82 | 0.55 | 1 | 1 | 1 | 1 | 3 |
| 181 | 340.33 | 8 | 6 | -0.79 | 0.56 | 1 | 147.68 | -1.07 | VS | 80.62 | 0.55 | 1 | 1 | 1 | 1 | 1 |
| 182 | 372.75 | 8 | 5 | -0.12 | 0.5 | 1 | 144.52 | -1.99 | VS | 84.71 | 0.55 | 0 | 1 | 1 | 1 | 0 |
| 183 | 338.31 | 8 | 5 | -0.49 | 0.56 | 1 | 139.98 | -1.21 | VS | 78.46 | 0.55 | 0 | 1 | 0 | 1 | 0 |

| | | | | | | | | | | | | | | | | |
|---|---|---|---|---|---|---|---|---|---|---|---|---|---|---|---|---|
| OTH | | | | | | | | | | | | | | | | |
| 184 | 282.29 | 4 | 0 | 2.8 | 0.18 | 2 | 52.6 | -3.88 | S | 77.7 | 0.55 | 0 | 0 | 0 | 0 | 0 |
| 185 | 254.24 | 4 | 2 | 2.41 | 0.07 | 0 | 74.6 | -4.16 | MS | 68.76 | 0.55 | 0 | 0 | 0 | 0 | 0 |
| 186 | 240.21 | 4 | 2 | 2.04 | 0 | 0 | 74.6 | -3.81 | S | 63.8 | 0.55 | 0 | 0 | 0 | 0 | 0 |
| 187 | 284.26 | 5 | 2 | 2.2 | 0.12 | 1 | 83.83 | -3.87 | S | 75.25 | 0.55 | 0 | 0 | 0 | 0 | 0 |
| 188 | 298.29 | 5 | 1 | 2.56 | 0.18 | 2 | 72.83 | -4.08 | MS | 79.72 | 0.55 | 0 | 0 | 0 | 0 | 0 |
| 189 | 268.26 | 4 | 1 | 2.6 | 0.12 | 1 | 63.6 | -4.02 | MS | 73.23 | 0.55 | 0 | 0 | 0 | 0 | 0 |
| 190 | 314.29 | 6 | 2 | 2.15 | 0.18 | 2 | 93.06 | -3.95 | S | 81.75 | 0.55 | 0 | 0 | 0 | 0 | 0 |
| 191 | 256.21 | 5 | 3 | 1.62 | 0 | 0 | 94.83 | -3.67 | S | 65.82 | 0.55 | 0 | 0 | 0 | 0 | 0 |
| 192 | 300.26 | 6 | 3 | 1.96 | 0.12 | 1 | 104.06 | -4.08 | MS | 77.28 | 0.55 | 0 | 0 | 0 | 0 | 0 |
| 193 | 336.29 | 8 | 5 | 0.05 | 0.38 | 1 | 144.52 | -2.02 | S | 80.23 | 0.55 | 0 | 0 | 1 | 1 | 0 |
| 194 | 332.35 | 6 | 3 | 1.73 | 0.44 | 2 | 104.06 | -3.01 | S | 86.81 | 0.55 | 0 | 0 | 0 | 0 | 0 |
| 195 | 268.26 | 4 | 1 | 2.63 | 0.12 | 1 | 63.6 | -4.02 | MS | 73.23 | 0.55 | 0 | 0 | 0 | 0 | 0 |
| 196 | 316.26 | 7 | 5 | 1.08 | 0.12 | 1 | 135.29 | -3.42 | S | 78.78 | 0.55 | 0 | 0 | 0 | 1 | 0 |
| 197 | 330.29 | 7 | 4 | 1.42 | 0.18 | 2 | 124.29 | -3.42 | S | 83.51 | 0.55 | 0 | 0 | 0 | 0 | 0 |
| 198 | 346.29 | 8 | 5 | 1.13 | 0.18 | 2 | 144.52 | -3.27 | S | 85.53 | 0.55 | 0 | 0 | 1 | 1 | 0 |
| 199 | 286.24 | 6 | 3 | 1.53 | 0.07 | 1 | 104.06 | -3.43 | S | 72.31 | 0.55 | 0 | 0 | 0 | 0 | 0 |
| 200 | 574.49 | 12 | 6 | 0.69 | 0.33 | 2 | 208.12 | -3.73 | S | 138.35 | 0.11 | 3 | 2 | 1 | 1 | 4 |
| 201 | 272.21 | 6 | 4 | 1.11 | 0 | 0 | 115.06 | -3.23 | S | 67.84 | 0.55 | 0 | 0 | 0 | 0 | 0 |
| 202 | 538.46 | 10 | 6 | 3.64 | 0.07 | 1 | 189.66 | -7.49 | PS | 140.56 | 0.17 | 2 | 2 | 1 | 1 | 3 |

| | | | | | | | | | | | | | | | |
|---|---|---|---|---|---|---|---|---|---|---|---|---|---|---|---|
| 203 | 298.29 | 5 | 1 | 2.63 | 0.18 | 2 | 72.83 | -4.08 | MS | 79.72 | 0.55 | 0 | 0 | 0 | 0 | 0 |
| 204 | 352.38 | 5 | 1 | 3.78 | 0.24 | 4 | 72.83 | -5.17 | MS | 98.48 | 0.55 | 0 | 0 | 0 | 0 | 0 |
| 205 | 284.26 | 5 | 2 | 2.25 | 0.12 | 1 | 83.83 | -3.87 | S | 75.25 | 0.55 | 0 | 0 | 0 | 0 | 0 |
| 206 | 300.26 | 6 | 4 | 1.4 | 0.12 | 1 | 115.06 | -3.56 | S | 76.75 | 0.55 | 0 | 0 | 0 | 0 | 0 |
| 207 | 328.27 | 7 | 3 | 1.48 | 0.12 | 3 | 121.13 | -3.33 | S | 81.68 | 0.55 | 0 | 0 | 0 | 0 | 0 |
| 208 | 398.41 | 7 | 2 | 2.95 | 0.36 | 3 | 102.29 | -4.69 | MS | 104.75 | 0.55 | 0 | 0 | 0 | 0 | 0 |

S: Soluble
PS: Poorly Soluble
MS: Moderately soluble
VS: Very Soluble

**Table S2.** Pharmacokinetics behavior of anthraquinones. Compounds number according to the main text.

| | | | | CYP1A2 inhibition | | | | |
|---|---|---|---|---|---|---|---|---|
| **Molecule** | **GI absorption** | **BBB permeant** | **Pgp substrate** | **CYP1A2** | **CYP2C19** | **CYP2C9** | **CYP2D6** | **CYP3A4** |
| **NIG** | | | | | | | | |
| **1** | Low | No | Yes | No | No | No | No | No |
| **2** | High | No | Yes | No | No | No | No | No |
| **3** | High | No | No | No | No | No | No | No |
| **4** | High | No | No | Yes | No | Yes | No | Yes |
| **5** | High | No | No | Yes | No | Yes | No | Yes |
| **6** | High | No | No | No | No | No | No | No |
| **7** | High | No | Yes | No | No | No | No | No |
| **8** | Low | No | No | No | No | No | No | No |
| **9** | High | No | Yes | No | No | No | No | No |
| **10** | Low | No | No | No | No | No | No | No |
| **ASP** | | | | | | | | |
| **7** | High | No | Yes | No | No | No | No | No |
| **8** | Low | No | No | No | No | No | No | No |
| **10** | Low | No | No | No | No | No | No | No |
| **11** | Low | No | No | No | No | Yes | No | No |
| **12** | Low | No | No | No | No | Yes | No | No |
| **13** | High | No | No | Yes | No | No | No | Yes |
| **14** | High | No | No | Yes | No | Yes | No | Yes |
| **15** | High | No | Yes | Yes | No | No | No | No |
| **16** | High | No | Yes | No | No | No | No | No |
| **17** | High | No | Yes | No | No | No | No | No |
| **18** | High | No | No | No | No | No | No | Yes |
| **19** | High | No | No | Yes | No | Yes | No | Yes |
| **20** | Low | No | Yes | No | No | No | No | No |
| **21** | Low | No | No | No | No | Yes | No | No |

| | | | | | | | | |
|---|---|---|---|---|---|---|---|---|
| **22** | Low | No | No | No | No | Yes | No | No |
| **23** | Low | No | No | No | No | Yes | No | No |
| **24** | Low | No | Yes | No | No | No | No | No |
| **25** | Low | No | No | No | No | No | No | No |
| **26** | High | No | No | No | No | Yes | Yes | Yes |
| **27** | High | No | No | No | No | Yes | Yes | Yes |
| **28** | High | No | No | Yes | No | Yes | Yes | Yes |
| **29** | High | No | No | No | No | Yes | No | Yes |
| **30** | High | No | Yes | No | No | Yes | Yes | Yes |
| **31** | High | No | Yes | No | No | Yes | Yes | Yes |
| **32** | High | No | No | No | Yes | Yes | Yes | Yes |
| **33** | High | No | No | Yes | No | Yes | No | Yes |
| **34** | High | No | No | No | No | No | No | No |
| **36** | High | No | Yes | No | No | Yes | No | Yes |
| **37** | High | No | Yes | No | No | No | No | No |
| **38** | High | No | No | No | No | Yes | Yes | Yes |
| **39** | Low | No | Yes | No | No | No | No | No |
| **40** | High | No | Yes | No | No | No | No | No |
| **41** | High | No | No | Yes | No | No | No | Yes |
| **42** | High | No | No | No | No | No | No | Yes |
| **43** | High | No | No | No | Yes | Yes | Yes | Yes |
| **44** | High | No | No | Yes | No | Yes | Yes | Yes |
| **45** | High | No | No | Yes | No | Yes | No | Yes |
| **46** | High | No | No | Yes | No | Yes | No | Yes |
| **47** | High | Yes | No | Yes | Yes | No | No | Yes |
| **48** | High | Yes | No | Yes | Yes | Yes | No | Yes |
| **49** | High | Yes | No | Yes | Yes | No | No | No |
| **50** | High | No | No | Yes | No | No | No | Yes |
| **51** | High | Yes | No | Yes | No | No | No | Yes |
| **52** | High | Yes | No | Yes | No | No | No | Yes |
| **53** | High | No | Yes | No | No | No | No | Yes |

| | | | | | | | | |
|---|---|---|---|---|---|---|---|---|
| **54** | High | No | No | Yes | No | No | No | Yes |
| **55** | High | No | No | Yes | No | No | No | Yes |
| **56** | High | No | No | Yes | No | No | No | Yes |
| **57** | High | Yes | No | Yes | Yes | Yes | No | Yes |
| **58** | High | No | No | Yes | No | Yes | No | Yes |
| **59** | High | No | No | Yes | No | No | No | Yes |
| **60** | High | No | No | Yes | No | No | No | Yes |
| **61** | Low | No | Yes | No | No | No | No | No |
| **62** | High | No | No | No | No | No | No | Yes |
| **63** | High | No | No | Yes | No | No | No | Yes |
| **64** | High | No | No | Yes | No | Yes | No | Yes |
| **65** | High | No | No | Yes | No | Yes | No | Yes |
| **66** | High | No | No | Yes | No | No | No | Yes |
| **67** | High | No | No | Yes | No | Yes | No | Yes |
| **68** | High | No | No | No | No | Yes | Yes | Yes |
| **69** | High | No | No | No | No | Yes | No | Yes |
| **70** | Low | No | No | No | Yes | Yes | No | Yes |
| **71** | Low | No | No | Yes | No | Yes | Yes | Yes |
| **72** | High | No | No | Yes | Yes | Yes | No | Yes |
| **73** | High | No | No | Yes | Yes | Yes | No | Yes |
| **74** | High | No | No | Yes | Yes | Yes | No | Yes |
| **75** | Low | No | No | No | Yes | Yes | No | Yes |
| **76** | High | No | No | Yes | No | Yes | No | Yes |
| **77** | High | No | No | Yes | No | Yes | No | Yes |
| **78** | High | No | No | Yes | Yes | Yes | No | Yes |
| **79** | High | No | No | Yes | Yes | Yes | No | Yes |
| **80** | High | No | No | No | Yes | Yes | No | Yes |
| **PEN** | | | | | | | | |
| **13** | High | No | No | Yes | No | No | No | Yes |

| | | | | | | | | |
|---|---|---|---|---|---|---|---|---|
| **17** | High | No | Yes | No | No | No | No | No |
| **26** | High | No | No | No | No | Yes | Yes | Yes |
| **27** | High | No | No | No | No | Yes | Yes | Yes |
| **37** | High | No | Yes | No | No | No | No | No |
| **81** | Low | No | No | No | No | Yes | No | No |
| **82** | Low | No | No | No | No | Yes | No | No |
| **83** | High | Yes | No | Yes | No | No | No | Yes |
| **84** | High | No | No | No | No | No | No | Yes |
| **85** | High | No | No | Yes | No | Yes | No | Yes |
| **86** | Low | No | No | No | No | No | No | No |
| **87** | Low | No | No | No | No | No | No | No |
| **88** | High | No | No | Yes | No | No | No | Yes |
| **89** | Low | No | Yes | No | No | Yes | No | No |
| **90** | Low | No | Yes | No | No | No | No | No |
| **91** | Low | No | Yes | No | No | Yes | No | Yes |
| **92** | Low | No | Yes | No | No | Yes | No | Yes |
| **93** | Low | No | Yes | No | No | No | No | No |
| **94** | Low | No | Yes | No | No | Yes | No | Yes |
| **95** | Low | No | Yes | No | No | Yes | No | Yes |
| **96** | Low | No | Yes | No | No | No | No | No |
| **97** | High | No | No | No | No | No | No | No |
| **98** | High | No | No | No | No | No | No | Yes |
| **STE** | | | | | | | | |
| **99** | Low | No | No | No | No | No | No | No |
| **100** | High | No | No | No | No | No | No | No |
| **101** | High | No | Yes | No | No | No | No | No |
| **102** | High | No | Yes | No | No | No | No | No |
| **103** | High | No | No | No | No | No | No | No |

| | | | | | | | | |
|---|---|---|---|---|---|---|---|---|
| **104** | High | No | No | No | No | No | No | No |
| **105** | High | No | No | No | No | No | No | No |
| **106** | High | No | Yes | No | No | No | No | No |
| **107** | Low | No | No | No | No | No | No | No |
| **108** | High | No | No | Yes | No | Yes | No | Yes |
| **109** | High | No | No | No | No | No | No | No |
| **110** | Low | No | Yes | No | No | No | No | No |
| **111** | Low | No | No | No | No | No | No | No |
| **112** | Low | No | No | No | No | Yes | No | No |
| **113** | Low | No | No | No | No | Yes | No | No |
| **114** | Low | No | No | No | Yes | Yes | No | No |
| **115** | Low | No | Yes | No | No | No | No | No |
| **116** | Low | No | Yes | No | No | No | No | No |
| **117** | Low | No | No | No | No | Yes | No | No |
| **118** | Low | No | No | No | Yes | Yes | No | No |
| **119** | Low | No | No | No | Yes | Yes | No | No |
| **120** | Low | No | No | No | Yes | No | No | No |
| **121** | Low | No | No | No | Yes | Yes | No | No |
| **122** | Low | No | No | No | Yes | Yes | No | No |
| **123** | Low | No | No | No | No | Yes | No | No |
| **124** | Low | No | No | No | No | Yes | No | No |
| **125** | Low | No | No | No | No | Yes | No | No |
| **126** | Low | No | No | No | No | Yes | No | No |
| **127** | Low | No | No | No | No | Yes | No | No |
| **128** | Low | No | No | No | No | Yes | No | No |
| **129** | Low | No | Yes | No | No | No | No | No |
| **130** | Low | No | No | No | Yes | Yes | No | No |

**ALT**

| | | | | | | | | |
|---|---|---|---|---|---|---|---|---|
| **100** | High | No | No | No | No | No | No | No |
| **101** | High | No | Yes | No | No | No | No | No |
| **102** | High | No | Yes | No | No | No | No | No |
| **104** | High | No | No | No | No | No | No | No |
| **105** | High | No | No | No | No | No | No | No |
| **107** | Low | No | No | No | No | No | No | No |
| **108** | High | No | No | Yes | No | Yes | No | Yes |
| **114** | Low | No | No | No | Yes | Yes | No | No |
| **131** | Low | No | No | No | No | Yes | No | No |
| **132** | Low | No | No | No | No | Yes | No | No |
| **133** | Low | No | No | No | No | Yes | No | No |
| **134** | Low | No | Yes | No | No | No | No | No |
| **135** | Low | No | No | No | No | Yes | No | No |
| **136** | High | No | Yes | No | No | No | No | No |
| **137** | High | No | Yes | No | No | No | No | No |
| **138** | High | No | Yes | No | No | No | No | No |
| **139** | High | No | Yes | No | No | No | No | No |
| **140** | Low | No | Yes | No | Yes | No | No | No |
| **141** | High | No | No | Yes | No | Yes | No | Yes |
| **142** | High | No | No | Yes | No | No | No | No |

| **TRI** | | | | | | | | |
|---|---|---|---|---|---|---|---|---|
| **13** | High | No | No | Yes | No | No | No | Yes |
| **83** | High | Yes | No | Yes | No | No | No | Yes |
| **84** | High | No | No | No | No | No | No | Yes |
| **143** | High | No | No | No | No | No | No | No |
| **144** | High | No | No | No | No | No | No | No |
| **145** | High | Yes | No | Yes | No | No | No | Yes |

| | | | | | | | | |
|---|---|---|---|---|---|---|---|---|
| **146** | High | No | No | Yes | No | No | No | Yes |
| **147** | High | Yes | No | Yes | Yes | No | No | Yes |
| **148** | High | No | No | No | No | No | No | No |
| **149** | High | No | No | Yes | No | No | No | Yes |
| **150** | High | No | No | No | No | No | No | No |
| **151** | High | No | No | No | No | No | No | No |
| **152** | High | Yes | No | Yes | No | No | No | Yes |
| **153** | High | Yes | No | Yes | No | Yes | No | Yes |
| **154** | High | No | No | No | No | No | No | No |
| **155** | High | No | No | No | No | No | No | No |

| **EUR** | | | | | | | | |
|---|---|---|---|---|---|---|---|---|
| **13** | High | No | No | Yes | No | No | No | Yes |
| **15** | High | No | Yes | Yes | No | No | No | No |
| **18** | High | No | No | No | No | No | No | Yes |
| **19** | High | No | No | Yes | No | Yes | No | Yes |
| **20** | Low | No | Yes | No | No | No | No | No |
| **60** | High | No | No | Yes | No | No | No | Yes |
| **63** | High | No | No | Yes | No | No | No | Yes |
| **97** | High | No | No | No | No | No | No | No |
| **154** | High | No | No | No | No | No | No | No |
| **156** | High | No | No | Yes | No | No | No | Yes |
| **157** | High | Yes | No | Yes | No | Yes | Yes | Yes |
| **158** | High | No | Yes | No | No | No | No | No |
| **159** | Low | No | Yes | No | No | No | No | No |
| **160** | Low | No | No | No | No | Yes | No | No |
| **161** | Low | No | Yes | No | No | No | No | No |
| **162** | Low | No | Yes | No | No | No | No | No |

| | | | | | | | | |
|---|---|---|---|---|---|---|---|---|
| **163** | High | No | No | No | No | No | No | No |
| **FUS** | | | | | | | | |
| **5** | High | No | No | Yes | No | Yes | No | Yes |
| **6** | High | No | No | No | No | No | No | No |
| **7** | High | No | Yes | No | No | No | No | No |
| **8** | Low | No | No | No | No | No | No | No |
| **10** | Low | No | No | No | No | No | No | No |
| **164** | High | No | No | No | No | No | No | No |
| **165** | High | No | Yes | No | No | No | No | No |
| **166** | High | No | Yes | No | No | No | No | No |
| **167** | Low | No | No | No | No | No | No | No |
| **168** | High | No | Yes | No | No | No | No | No |
| **169** | High | Yes | No | Yes | Yes | Yes | No | Yes |
| **170** | High | No | No | No | No | Yes | No | Yes |
| **ENG** | | | | | | | | |
| **171** | Low | No | Yes | No | No | Yes | No | No |
| **172** | Low | No | Yes | No | No | Yes | No | No |
| **173** | Low | No | Yes | No | No | Yes | No | No |
| **174** | Low | No | Yes | No | No | No | No | No |
| **175** | Low | No | No | No | No | No | No | No |
| **176** | Low | No | No | No | No | No | No | No |
| **177** | Low | No | Yes | No | No | No | No | No |

| | | | | | | | | |
|---|---|---|---|---|---|---|---|---|
| **SPO** | | | | | | | | |
| **101** | High | No | Yes | No | No | No | No | No |
| **178** | High | No | No | No | No | No | No | No |
| **179** | Low | No | No | No | No | No | No | No |
| **180** | Low | No | No | No | No | No | No | No |
| **181** | Low | No | No | No | No | No | No | No |
| **182** | Low | No | No | No | No | No | No | No |
| **183** | Low | No | No | No | No | No | No | No |

| | | | | | | | | |
|---|---|---|---|---|---|---|---|---|
| **OTH** | | | | | | | | |
| **184** | High | Yes | No | Yes | Yes | Yes | No | Yes |
| **185** | High | Yes | No | Yes | No | No | No | Yes |
| **186** | High | Yes | No | Yes | No | No | No | Yes |
| **187** | High | No | No | Yes | No | No | No | Yes |
| **188** | High | Yes | No | Yes | Yes | Yes | No | Yes |
| **189** | High | Yes | No | Yes | Yes | Yes | No | Yes |
| **190** | High | No | No | Yes | No | Yes | No | Yes |
| **191** | High | No | No | No | No | No | No | Yes |
| **192** | High | No | No | Yes | No | Yes | No | Yes |
| **193** | Low | No | No | No | No | No | No | No |
| **194** | High | No | Yes | No | No | No | No | No |
| **195** | High | Yes | No | Yes | Yes | Yes | No | Yes |
| **196** | High | No | No | No | No | No | No | No |
| **197** | High | No | Yes | No | No | No | No | Yes |
| **198** | Low | No | Yes | No | No | No | No | Yes |
| **199** | High | No | No | No | No | No | No | Yes |
| **200** | Low | No | Yes | No | Yes | No | No | No |
| **201** | High | No | No | No | No | No | No | Yes |
| **202** | Low | No | No | No | Yes | Yes | No | No |

| | | | | | | | | |
|---|---|---|---|---|---|---|---|---|
| **203** | High | Yes | No | Yes | Yes | Yes | No | Yes |
| **204** | High | Yes | No | Yes | Yes | Yes | No | Yes |
| **205** | High | No | No | Yes | No | Yes | No | Yes |
| **206** | High | No | No | No | No | No | No | No |
| **207** | High | No | No | No | No | No | No | No |
| **208** | High | No | Yes | No | No | Yes | No | Yes |

**Figure S1**: List of 20 anthraquinones-containing compounds with promising drug likeness properties

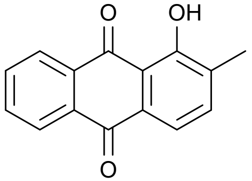

**47**

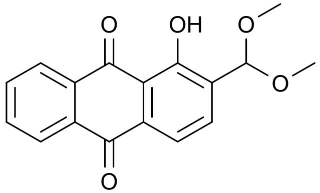

**48**

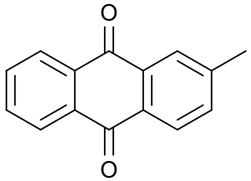

**49**

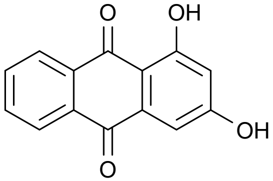

**51**

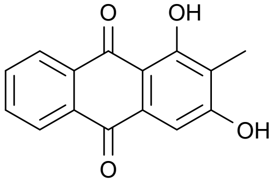

**52**

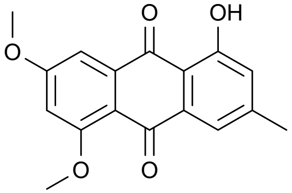

**57**

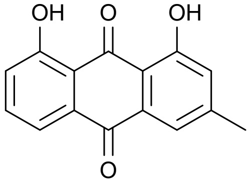

**83**

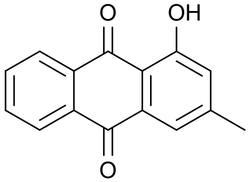

**147**

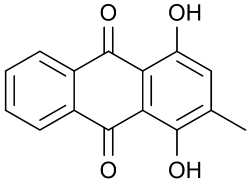

**152**

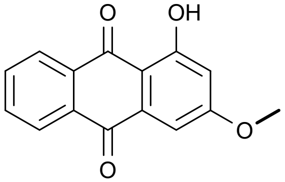

**153**

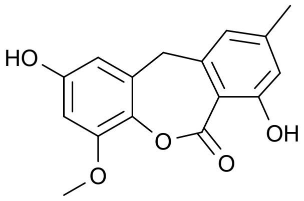

**157**

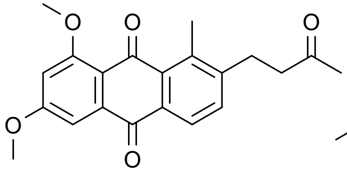

**169**

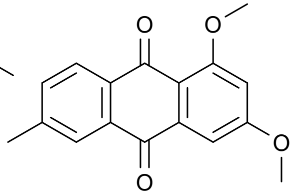

**184**

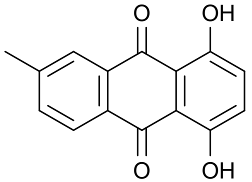

**185**

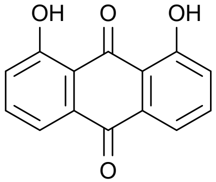

**186**

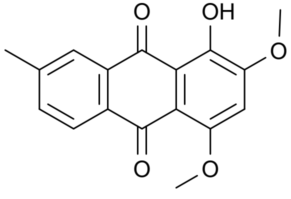

**188**

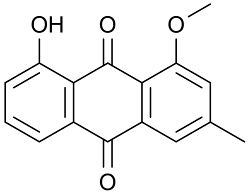

**189**

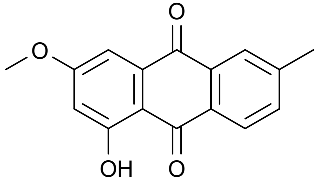

**195**

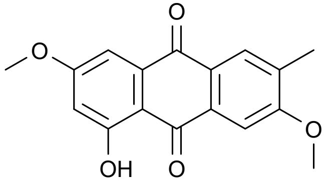

**203**

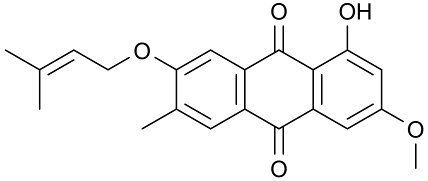

**204**
